# Composition-engineered intrinsic stability in 2D perovskite optoelectronic synapses for neuromorphic vision

**DOI:** 10.1093/nsr/nwag453

**Published:** 2026-07-22

**Authors:** Honglai Li, Wei Li, Herong Sheng, Changhao Liu, Wenjun Yang, Jinpeng Zhao, Shida Feng, Biyuan Zheng, Chenglin He, Jiahuan Ren, Liang Ma, Tingzhao Fu, Chun Zhao, Yuxiao Fang, Weihao Zheng, Dong Li, Zhihong Zhu, Anlian Pan

**Affiliations:** College of Physics Science and Technology, Hebei University, Baoding 071002, China; College of Physics Science and Technology, Hebei University, Baoding 071002, China; School of Advanced Technology, Xi’an Jiaotong-Liverpool University, Suzhou 215123, China; Department of Electrical Engineering and Electronics, University of Liverpool, Liverpool L69 3BX, UK; College of Physics Science and Technology, Hebei University, Baoding 071002, China; College of Physics Science and Technology, Hebei University, Baoding 071002, China; College of Physics and Electronic Engineering, Hengyang Normal University, Hengyang 421008, China; College of Physics Science and Technology, Hebei University, Baoding 071002, China; College of Advanced Interdisciplinary Studies & Hunan Provincial Key Laboratory of Novel Nano-Optoelectronic Information Materials and Devices, National University of Defense Technology, Changsha 410073, China; College of Advanced Interdisciplinary Studies & Hunan Provincial Key Laboratory of Novel Nano-Optoelectronic Information Materials and Devices, National University of Defense Technology, Changsha 410073, China; College of Physics Science and Technology, Hebei University, Baoding 071002, China; College of Physics and Electronic Engineering, Hengyang Normal University, Hengyang 421008, China; College of Advanced Interdisciplinary Studies & Hunan Provincial Key Laboratory of Novel Nano-Optoelectronic Information Materials and Devices, National University of Defense Technology, Changsha 410073, China; School of Advanced Technology, Xi’an Jiaotong-Liverpool University, Suzhou 215123, China; School of Advanced Technology, Xi’an Jiaotong-Liverpool University, Suzhou 215123, China; College of Advanced Interdisciplinary Studies & Hunan Provincial Key Laboratory of Novel Nano-Optoelectronic Information Materials and Devices, National University of Defense Technology, Changsha 410073, China; Hunan Institute of Optoelectronic Integration, College of Materials Science and Engineering, Hunan University, Changsha 410082, China; College of Advanced Interdisciplinary Studies & Hunan Provincial Key Laboratory of Novel Nano-Optoelectronic Information Materials and Devices, National University of Defense Technology, Changsha 410073, China; Hunan Institute of Optoelectronic Integration, College of Materials Science and Engineering, Hunan University, Changsha 410082, China; School of Physics and Electronics, Hunan Normal University, Changsha 410081, China

**Keywords:** stability, perovskite, synapse, neuromorphic vision

## Abstract

Operational instability remains a key obstacle for halide perovskites in neuromorphic optoelectronics, where sustained and reproducible photoresponses are required for in-sensor computing. Here, we report vapor-phase growth of composition-tunable two-dimensional (2D) inorganic perovskites, Cs*_x_*Rb_1−_*_x_*PbBr_3_ (0 ≤ *x* ≤ 1), and show that A-site cation alloying provides an effective route to simultaneously improve optical performance and structural robustness. By mapping the full Cs–Rb compositional space, we identify a narrow intermediate regime (*x* ≈ 0.8) where lattice strain is minimized and defect formation is suppressed. Perovskites in this regime exhibit substantially enhanced resistance to moisture, thermal stress and ion migration compared with either end member. These stability gains translate directly to device operation, enabling broadband photodetectors with fast response, high reproducibility and long-term durability. Using the optimized Cs_0.81_Rb_0.19_PbBr_3_ composition, we further demonstrate optoelectronic synaptic devices that reproduce key synaptic functions, including paired-pulse facilitation and light-programmable synaptic weights. Integrated into a spiking neural network, the devices enable real-time fire detection with 85% accuracy while maintaining stable performance over 7 days. This work establishes composition-engineered 2D inorganic perovskites as a robust platform for neuromorphic vision and energy-efficient in-sensor computing.

## Teaser

A-site alloying in 2D Cs*_x_*Rb_1__−_*_x_*PbBr3 perovskites minimizes the lattice strain, and enables stable optoelectronic synapses for fire detection with 85% accuracy and <1% drift over a week.

## INTRODUCTION

Neuromorphic optoelectronic systems aim to mimic biological perception by integrating sensing, memory and computation into a single hardware framework [1–7]. Achieving this functionality requires semiconductor materials that combine strong light–matter interaction, tunable optoelectronic properties and long-term operational stability under continuous optical and electrical stimuli [8,9]. Two-dimensional (2D) semiconductors are attractive in this context because their reduced dimensionality can mitigate defect formation and facilitate heterogeneous integration [10,11]. Yet, identifying material platforms that deliver both high performance and reliable stability for neuromorphic vision remains challenging.

Metal halide perovskites have emerged as promising candidates due to their high absorption coefficients [12], defect tolerance [13] and compositionally tunable bandgaps [14], enabling photodetectors, light-emitting devices and lasers [15–20]. All-inorganic perovskites are particularly appealing because they avoid volatile organic components and typically show improved thermal and environmental stability compared with hybrid analogues [21,22]. However, even fully inorganic perovskites are still vulnerable to phase instability, lattice distortion and ion migration, especially under prolonged illumination, elevated temperature and ambient exposure [23,24]. These degradation pathways fundamentally limit their suitability for neuromorphic applications, which demand persistent and reproducible photoresponses over extended operation.

A-site cation engineering has proven effective for improving perovskite stability and tuning the electronic structure [25]. Partial substitution of Cs^+^ with smaller alkali cations such as Rb^+^, K^+^ or Na^+^ can relieve lattice strain, suppress unfavorable phase transitions and reduce defect densities [26–28]. Although A-site alloying has improved stability and device performance in bulk and thin-film perovskites [29], a systematic understanding of how continuous compositional tuning governs the structural robustness and optoelectronic functionality of 2D inorganic perovskites—and how these effects translate into neuromorphic device performance—remains limited.

Here, we demonstrate vapor-phase growth of fully composition-tunable 2D Cs*_x_*Rb_1−_*_x_*PbBr_3_ perovskites (0 ≤ *x* ≤ 1), enabling precise control over A-site cation ratios across the entire alloy range (the term ‘2D’ herein describes the nanosheet morphology, not crystallographic layered RP/DJ-phase structure). By correlating composition with crystal structure, optical properties and environmental stability, we identify an optimal intermediate alloy, Cs_0.81_Rb_0.19_PbBr_3_, that exhibits strong resistance to moisture, thermal stress and ion migration. Building on this stability-optimized platform, we realize broadband photodetectors and optoelectronic synapses that reproduce key synaptic behaviors. Finally, integration into a spiking neural network (SNN) enables real-time fire detection with 85% accuracy and negligible degradation over 1 week. These results highlight compositionally engineered 2D inorganic perovskites as a viable materials basis for robust neuromorphic vision and energy-efficient in-sensor computing.

## RESULTS AND DISCUSSION

Single-crystalline Cs*_x_*Rb_1−_*_x_*PbBr_3_ (0 ≤ *x* ≤ 1) nanosheets were directly grown on freshly exfoliated mica (001) substrates by vapor-phase deposition, yielding large-area 2D crystals with uniform compositions across the full alloy range (Fig. 1a–c). To accommodate the distinct volatility and reaction kinetics of different A-site ratios, the growth temperature was adjusted from 350 to 500°C (see Experimental section in the Supplementary data). As illustrated in Fig. 1a, the alloyed perovskite adopts a quasi-rectangular 2D lattice consisting of corner-sharing [PbBr_6_]^4−^ octahedra extending along the *a*- and *c*-axes, while Cs^+^ and Rb^+^ occupy the cuboctahedral A-sites. Replacing Cs^+^ (1.67 Å) with the smaller Rb^+^ (1.48 Å) is expected to contract the lattice and induce subtle octahedral tilting, leading to a gradual structural distortion of the 2D framework [30–32]. Across all compositions, the nanosheets exhibit well-defined rectangular morphologies with lateral sizes up to ∼128 μm and thickness down to a few nanometers, consistent with strongly anisotropic layer-by-layer growth (Fig. 1c). Atomic force microscopy (AFM) and scanning electron microscopy analyses further reveal smooth surfaces and excellent thickness uniformity, with the thinnest nanosheets reaching ∼7.61 nm (Fig. 1d, and Figs S1 and S2). Elemental mapping confirms that both end members (CsPbBr_3_ and RbPbBr_3_) and intermediate alloys maintain homogeneous elemental distributions without detectable compositional fluctuation (Figs S4 and S5), indicating reliable stoichiometric control during vapor growth. X-ray diffraction (XRD) patterns show a systematic evolution of lattice parameters with increasing Rb incorporation (Fig. 1e). Both CsPbBr_3_ and RbPbBr_3_ nanosheets match their reference patterns and exhibit a monoclinic phase at room temperature. Diffraction peaks at ∼15.4°, 21.7° and 30.6° can be indexed to the (001), (011) and (002) planes, respectively, while the pronounced (200) reflection suggests preferential in-plane crystal growth. Notably, all Bragg peaks shift monotonically toward higher angles as the Rb fraction increases, consistent with lattice contraction induced by substitution of larger Cs^+^ ions with smaller Rb^+^ ions [33]. No additional peaks or splitting are observed throughout the entire composition range, indicating single-phase solid-solution formation without phase segregation. X-ray photoelectron spectroscopy (XPS) provides further evidence of successful A-site alloying (Fig. 1f) [34]. The relative intensities of Cs 3*d* and Rb 3*d* signals evolve monotonically with composition, accompanied by small binding-energy shifts that reflect changes in the local A-site chemical environment. In contrast, the Pb 4*f* and Br 3*d* spectra remain nearly unchanged (Fig. S3), suggesting that alloying primarily modifies the A-site sublattice while preserving the integrity of the [PbBr_6_]^4−^ framework.

**Figure 1.**
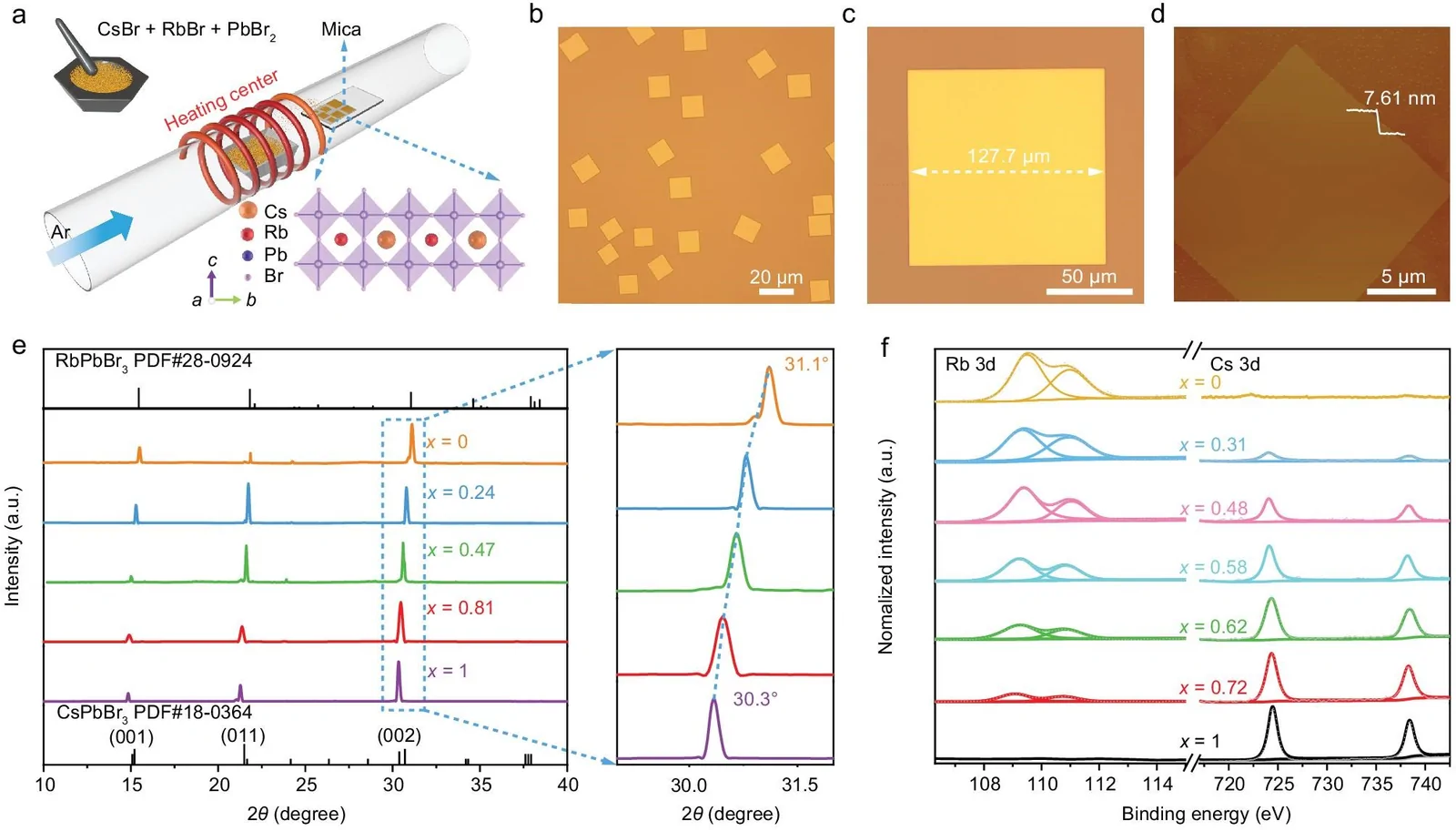
(a) Growth illustration of Cs*_x_*Rb_1−_*_x_*PbBr_3_ (0 ≤ *x* ≤ 1) nanosheets. (b) Typical optical image of the as-grown Cs*_x_*Rb_1−_*_x_*PbBr_3_ (0 ≤ *x* ≤ 1) nanosheets on mica substrate. (c) Optical microscopy (OM) image of a representative Cs*_x_*Rb_1−_*_x_*PbBr_3_ (0 ≤ *x* ≤ 1) nanosheet with a lateral size ≈ 127.7 μm. (d) AFM image and thickness information of a Cs*_x_*Rb_1−_*_x_*PbBr_3_ (0 ≤ *x* ≤ 1) nanosheet. (e) XRD patterns of Cs*_x_*Rb_1−_*_x_*PbBr_3_ (*x* = 1, 0.81, 0.47, 0.24, 0). (f) Cs 3*d* and Rb 3*d* core level XPS spectra.

Atomic-resolution high-angle annular dark-field scanning transmission electron microscopy (HAADF-STEM) was used to directly examine the structural quality and compositional uniformity of the alloyed nanosheets (Fig. 2). Low-magnification HAADF-STEM imaging shows atomically flat surfaces and well-defined edges, consistent with high-quality single-crystalline growth (Fig. 2a). The corresponding selected area electron diffraction (SAED) pattern exhibits sharp diffraction spots (Fig. 2b and Fig. S6), confirming long-range crystallographic order. For the Cs_0.45_Rb_0.55_PbBr_3_ alloy, lattice spacings measured along the (100) and (001) directions are 0.576 and 0.583 nm, respectively, both smaller than those of pristine CsPbBr_3_, consistent with the expected lattice contraction upon Rb incorporation. Energy dispersive X-ray spectroscopy (EDS) mapping acquired from the region marked in Fig. 2a shows a uniform distribution of Cs, Rb, Pb and Br across the nanosheet (Fig. 2c), and the corresponding spectrum confirms the intended stoichiometry (Fig. 2d).

**Figure 2.**
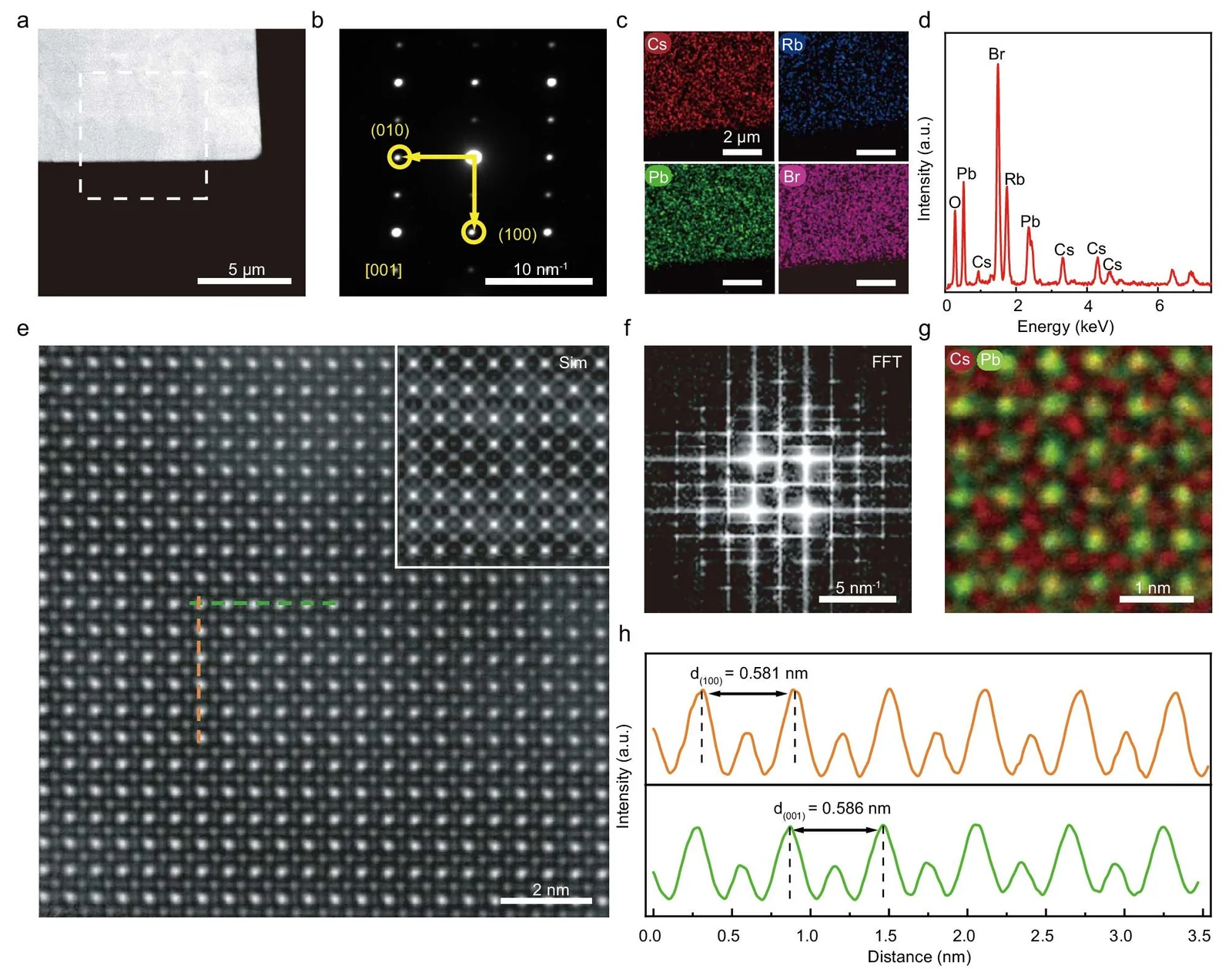
(a and b) Low-magnification HAADF-STEM and SAED images of a representative Cs*_x_*Rb_1−_*_x_*PbBr_3_ nanosheet. (c) EDS mapping of the elements Cs, Rb, Pb and Br in the white box of panel (a). (d) EDS spectrum of the Cs*_x_*Rb_1−_*_x_*PbBr_3_ nanosheet. (e) Atomic-resolution HAADF-STEM image of a typical Cs*_x_*Rb_1−_*_x_*PbBr_3_ nanosheet. Inset: simulative image of the crystal. (f) FFT diffraction spots of panel (e). (g) Atomic-resolution EDS elemental mapping of Cs and Pb. (h) Intensity profile along the dashed lines in panel (e).

Figure 2e shows an atomic-resolution HAADF-STEM image of Cs_0.81_Rb_0.19_PbBr_3_ viewed along the (001) zone axis. The contrast is dominated by the projected [PbBr_6_]^4−^ octahedral framework, while the A-site sublattice exhibits intensity variations consistent with mixed Cs/Rb occupancy. Importantly, no contrast modulation or domain-like features are observed, suggesting random alloying rather than nanoscale phase separation. The experimental image agrees well with the simulated structure (inset), supporting the structural assignment. Fast Fourier transform (FFT) analysis reveals well-defined diffraction spots with high symmetry (Fig. 2f), further indicating single crystallinity. Atomic-resolution EDS mapping resolves the spatial distribution of Cs and Pb (Fig. 2g), and the measured lattice spacings along the (100) and (001) directions are 0.581 and 0.586 nm, respectively (Fig. 2h), consistent with the composition-dependent lattice evolution. Similar results were obtained for Cs_0.72_Rb_0.28_PbBr_3_ (Fig. S7), confirming that phase purity and alloy homogeneity are preserved across different Cs/Rb ratios. Together, these results establish that continuous A-site alloying enables systematic lattice modulation in 2D Cs*_x_*Rb_1−_*_x_*PbBr_3_ nanosheets without compromising crystalline integrity.

The optical properties of Cs*_x_*Rb_1−_*_x_*PbBr_3_ (0 ≤ *x* ≤ 1) nanosheets can be continuously tuned through A-site composition. Increasing the Rb fraction results in a monotonic blue shift of the photoluminescence (PL) peak from 525 nm for CsPbBr_3_ (*x* = 1) to 447 nm for RbPbBr_3_ (*x* = 0), covering the full blue-to-green spectral range (Fig. 3a). All spectra remain narrow and symmetric, indicating that alloying does not introduce significant inhomogeneous broadening or strong defect-related emission. Bandgap arrangement of the alloys (Fig. S8) shows that substituting Cs with Rb increases the bandgap by widening the separation between the conduction-band minimum and valence-band maximum, consistent with the experimentally observed PL evolution [35]. This agreement indicates that A-site alloying provides a direct and reliable route to bandgap engineering in 2D inorganic perovskites. Under 400 nm femtosecond pulsed excitation, all compositions exhibit stimulated emission with composition-tunable lasing wavelengths (Fig. 3b and Fig. S9). The emission intensity as a function of excitation power shows the characteristic threshold behavior expected for lasing (Fig. S10). Rate-equation analysis was applied to quantify the lasing dynamics (see Note S1 of the Supplementary data) [36–40]. The extracted lasing thresholds and spontaneous emission coupling factors (*β*) remain comparable across the Cs–Rb range (Fig. 3c), suggesting that optical gain and cavity quality are largely maintained despite the composition-driven lattice contraction.

**Figure 3.**
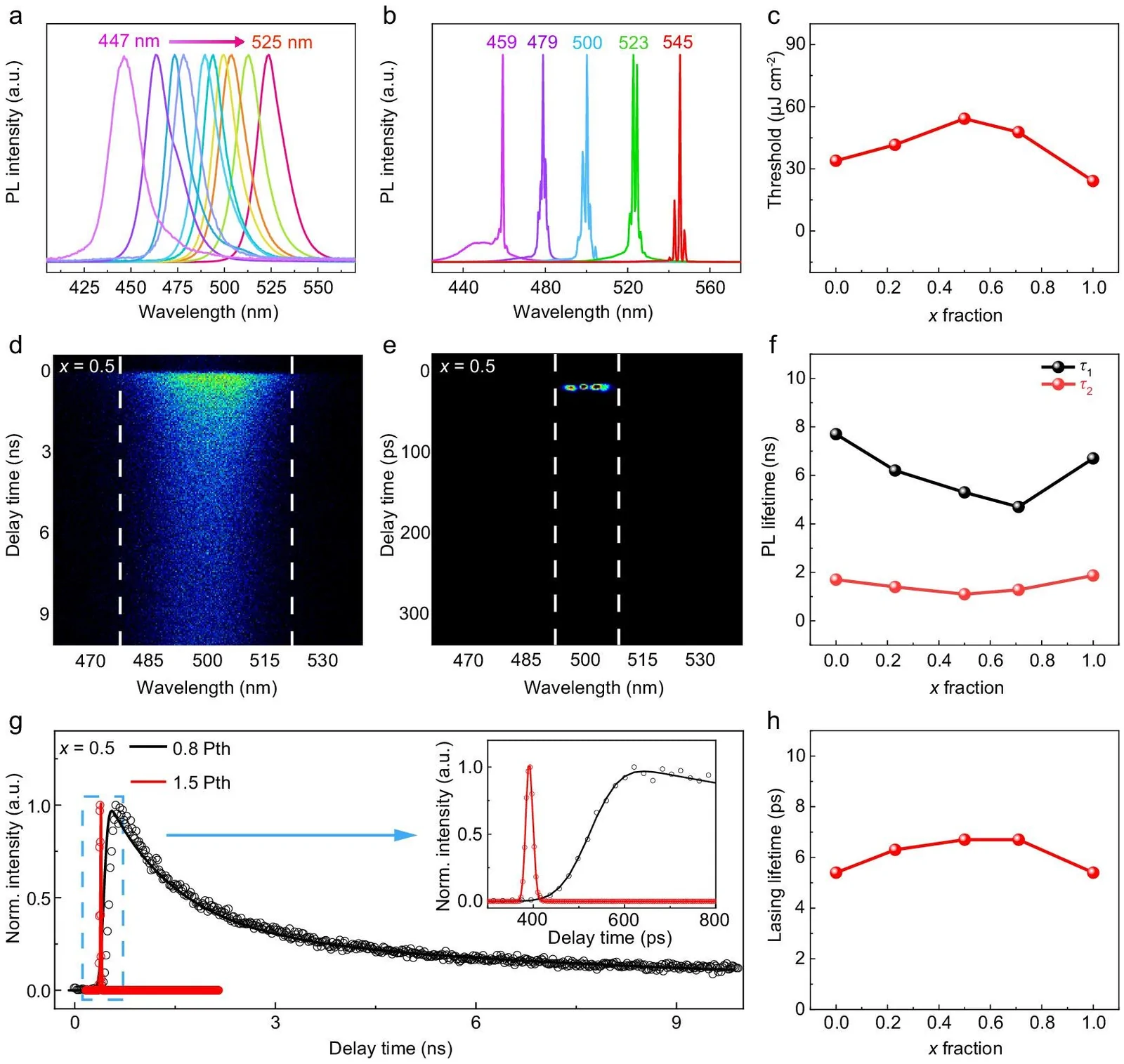
(a) PL spectra of Cs*_x_*Rb_1−_*_x_*PbBr_3_ (0 ≤ *x* ≤ 1) nanosheets. (b) The emission spectra of Cs*_x_*Rb_1−_*_x_*PbBr_3_ (0 ≤ *x* ≤ 1) nanosheets. (c) Lasing thresholds extracted from the selected spectra in panel (b). Streak-camera images of a Cs_0.5_Rb_0.5_PbBr_3_ nanosheet recorded under excitation power of panels (d) 0.8 P_th_ (threshold excitation power) and (e) 1.5 P_th_. (f) Deconvolution fitting results of TRPL curves of five compositions. (g) Typical PL-decay curve obtained at two different excitation powers (0.8, 1.5 P_th_). (h) Fitting parameters for the lasing decay curves of the representative alloyed compositions.

Time-resolved photoluminescence (TRPL) measurements further reveal that carrier recombination dynamics are only weakly dependent on composition. Below threshold, all nanosheets exhibit nanosecond-scale decay dominated by spontaneous recombination, whereas above threshold the lifetime collapses into the picosecond regime due to stimulated emission. Figure 3d and e show representative streak-camera images of a Cs_0.5_Rb_0.5_PbBr_3_ nanosheet recorded at 0.8 and 1.5 P_th_, respectively, with similar behavior observed for other compositions (Figs S11–S14). The transition from spontaneous to stimulated emission is accompanied by a sharp acceleration of decay kinetics. Figure 3g plots the excited-state decay curves of Cs_0.5_Rb_0.5_PbBr_3_ nanosheet under 0.8 and 1.5 P_th_ excitation intensities, respectively. Further, the two time-resolved fluorescence curves underwent exponential decay fitting, including deconvolution of the instrumental response curve. The PL decay curves were fitted using a multi-exponential model accounting for instrumental response, as follows [41]:


(1) \begin{eqnarray*}N\left( t \right) \propto I\left( t \right)\ = \ \sum {A}_i {\rm exp}\left( { - \frac{t}{{{\tau }_i}}} \right).\end{eqnarray*}

Here, *N*(*t*) is the total carrier density at delay time *t*, which is proportional to the fluorescence intensity *I*(*t*). A*_i_* (*i* = 1, 2 and 3) are the weight percentages, representing the carrier density contribution of different lifetime components. *τ_i_* (*i* = 1, 2 and 3) are the decay time constants. The fitting results are summarized in Table S1. For all compositions, the effective lifetime decreases from ∼5.3 ns below threshold to ∼6.7 ps above threshold. Importantly, the extracted spontaneous and stimulated recombination components show minimal variation with composition (Fig. 3f–h), indicating that A-site alloying primarily tunes the band structure while introducing little additional non-radiative loss.

To assess device-level performance, individual photodetectors were fabricated by transferring Au electrodes onto single Cs*_x_*Rb_1−_*_x_*PbBr_3_ nanosheets, defining a channel length of 5 μm (Fig. 4a). Under 405 nm illumination, the photocurrent shows a pronounced dependence on A-site composition (Fig. 4b). As the Rb fraction increases, the photocurrent decreases from 41.6 nA for CsPbBr_3_ to 0.31 nA for RbPbBr_3_. This trend is consistent with the stronger lattice contraction and strain introduced by Rb incorporation, which can enhance carrier scattering and reduce transport efficiency. Responsivity (*R*) and external quantum efficiency (EQE) were extracted using Equations (2) and (3):


(2) \begin{eqnarray*}R = \ \frac{{{I}_{{\mathrm{ph}}} - {I}_{\mathrm{d}}}}{{P \times S}},\end{eqnarray*}

where *I*_ph_, *I*_d_, *P* and *S* represent the steady-state photocurrent (extracted from the saturated plateau of the photoresponse curve), dark current, optical irradiance and active area (100 μm^2^), respectively [42]. EQE (%) is positively correlated with responsivity (*R*) and is defined by the equation


(3) \begin{eqnarray*}E = \frac{{R\ \times \ h\ \times \ c}}{{e\ \times \ \lambda }},\end{eqnarray*}

where *h, c, e* and *λ* denote Planck’s constant (*h* ≈ 6.63 × 10^−34^ J s), the speed of light (*c* ≈ 3 × 10^8^ m s^−1^), the electron charge (*e* ≈ 1.602 × 10^−19^ C) and the wavelength of incident light, respectively [43]. As summarized in Fig. 4c, both *R* and EQE increase monotonically with the Cs content. At an illumination intensity of 3.936 mW cm^−2^, *R* increases from 0.23 A W^−1^ (RbPbBr_3_) to 10.37 A W^−1^ (CsPbBr_3_), while EQE rises from 0.71% to 31.78%. Spectral response measurements reveal broadband sensitivity spanning 375–520 nm. For the Cs_0.48_Rb_0.52_PbBr_3_ device, the responsivity reaches a maximum of 9.41 A W^−1^ at ∼460 nm (Fig. 4d and e), consistent with its optical bandgap. The peak sensitivity wavelength can be continuously tuned with composition (inset of Fig. 4e), reflecting the bandgap modulation enabled by A-site alloying. In addition to its spectral tunability, the Cs_0.48_Rb_0.52_PbBr_3_ photodetector exhibits fast transient response, with a rise time (defined as the time required for the current to increase from 10% to 90% of its steady-state value) of ∼0.2 ms and a fall time (from 90% to 10%) of ∼1.615 ms (Fig. 4f). The device maintains stable switching behavior up to modulation frequencies of 100 Hz without significant distortion (Fig. 4g), and shows reproducible photocurrent responses under periodic illumination at different wavelengths (Fig. 4h). Comparable trends were observed for other compositions (Figs S15–S23).

**Figure 4.**
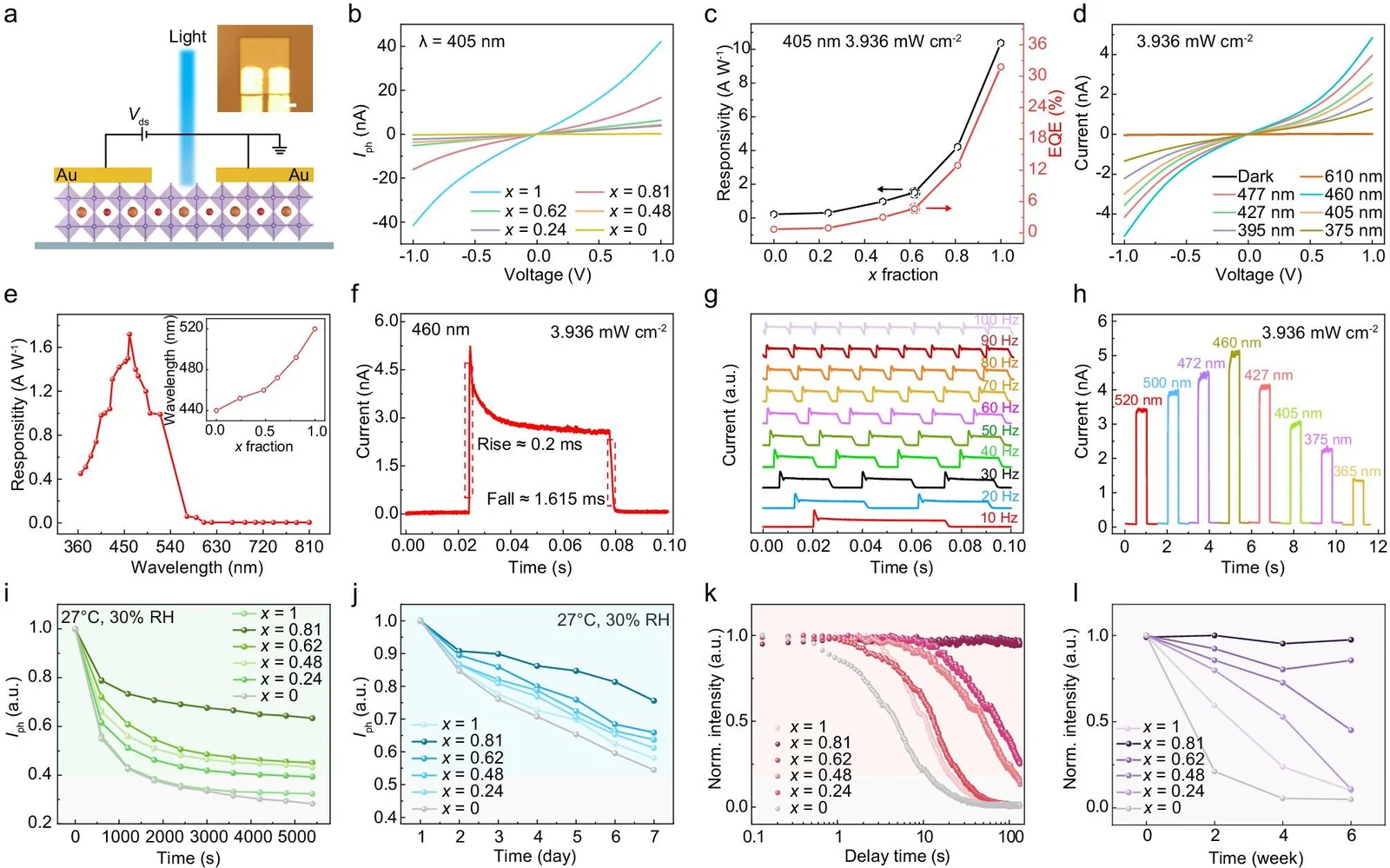
(a) Schematic illustration of an individual nanosheet-based photodetector. Inset: OM image of a typical device (scale bar: 10 μm). (b) Composition-dependent *I*–*V* characteristics measured under 405 nm illumination (3.936 mW cm^−2^). (c) *R* and EQE as a function of fraction (*x*). (d) Wavelength-dependent *I*–*V* curves of the Cs_0.48_Rb_0.52_PbBr_3_ device. (e) Wavelength-dependent *R* at 1 V bias. Inset: the peak sensitivity wavelength of the alloy as a function of the atomic percentage of component *x*. (f) Rise and fall time of the Cs_0.48_Rb_0.52_PbBr_3_ PD measured under 1 V. (g) Variable frequency-dependent transient photoresponse curves of Cs_0.48_Rb_0.52_PbBr_3_ PD with frequency at 1 V. (h) The *I*–*T* curves under periodic illumination at different wavelengths with a 1 V bias. (i) Time stability of devices at same humidity level of Cs*_x_*Rb_1−_*_x_*PbBr_3_ (0 ≤ *x* ≤ 1) nanosheets PDs. (j) Long-term environmental stability of the photodetector devices (monitored via photocurrent *I*_ph_) stored in air (27°C, 30% RH) for 7 days. (k) Normalized PL intensity evolution of the alloyed nanosheets as a function of delay time. (l) Long-term optical stability of Cs*_x_*Rb_1−_*_x_*PbBr_3_ (0 ≤ *x* ≤ 1) nanosheets, characterized by the evolution of normalized PL intensity over 6 weeks.

While high responsivity is important, neuromorphic vision systems place an equally stringent requirement on operational stability. We therefore systematically evaluated environmental, thermal and optical stability across the entire Cs–Rb compositional range. Notably, a pronounced stability maximum emerges in a narrow intermediate regime near *x* ≈ 0.8. Under continuous illumination at 30% relative humidity (RH), devices based on intermediate alloys retain photocurrent far more effectively than either end member (Fig. 4i and Fig. S24). Among all compositions, Cs_0.81_Rb_0.19_PbBr_3_ shows the highest photocurrent retention, followed by *x* = 0.62, 0.48 and 0.24, whereas CsPbBr_3_ and RbPbBr_3_ degrade most rapidly. This stability hierarchy remains consistent during long-term storage in ambient air (27°C, 30% RH) (Fig. 4j) and under thermal stress up to 380 K (Figs S25 and S26), indicating that the enhanced durability is intrinsic to the optimized alloy composition rather than a measurement artifact. Optical stability was further evaluated by monitoring PL intensity. To isolate intrinsic lattice stability from moisture- and oxygen-induced degradation, nanosheets were encapsulated with a polymethyl methacrylate (PMMA) layer [44,45] during PL measurements (Fig. 4k and l). Under continuous excitation and prolonged storage, Cs_0.81_Rb_0.19_PbBr_3_ exhibits minimal PL quenching, whereas both end members show pronounced intensity loss. The consistent trends observed in electrical stability (photocurrent retention) and optical stability (PL retention) suggest that moderate Rb incorporation effectively suppresses defect formation and ion migration while avoiding the excessive lattice distortion present at high Rb fractions. This Rb-dependent stability arises from the synergistic effects of lattice tuning and defect passivation. The smaller Rb^+^ ion adjusts the Goldschmidt tolerance factor from ∼0.81 (pristine CsPbBr_3_) to the optimal 0.9–1.0 range at 10%–20% doping, effectively suppressing lattice distortion and halide ion migration. Low-dose Rb also passivates undercoordinated Pb^2+^ and halide vacancies at grain boundaries, blocking defect-mediated degradation. Conversely, excessive Rb (>20%) causes severe lattice mismatch, RbPbBr_3_ impurity phase formation and phase separation, disrupting structural integrity and reducing long-term stability. Together, these results define a narrow and well-resolved compositional window where lattice strain is optimally balanced, yielding improved structural robustness without compromising optoelectronic function. This intrinsic stability advantage provides a key material basis for the neuromorphic devices discussed below. For neuromorphic synaptic devices, long-term stability, repeatable plasticity and reliable conductance modulation are essential. Although CsPbBr_3_ offers higher photoresponsivity, it suffers from severe degradation under prolonged illumination, retaining only 30% of its photocurrent after 5400 s. In contrast, Cs_0.81_Rb_0.19_PbBr_3_ achieves an optimal balance between optoelectronic performance and structural durability. Its responsivity is sufficient to realize all necessary synaptic functions, while the greatly enhanced stability ensures reliable and repeatable operation. Thus, this composition is a rational choice based on the core requirements of neuromorphic optoelectronics. For subsequent synaptic measurements, the same PMMA encapsulation strategy was employed to minimize direct exposure to ambient oxygen and moisture.

Reliable neuromorphic operation requires synaptic plasticity that remains reproducible under repeated stimulation and external perturbations. Guided by the compositional stability analysis above, Cs_0.81_Rb_0.19_PbBr_3_ was selected as the representative platform for optoelectronic synapse demonstrations. The resulting photosynaptic device integrates light sensing and memory within a single architecture (Fig. 5a), enabling direct conversion of optical stimuli into synaptic weight modulation. To enhance synaptic plasticity, a 20 nm HfO_2_ charge-trapping layer was introduced, enabling persistent modulation of the channel conductance through photogenerated carrier trapping [46]. To clarify the distinct roles of the HfO_2_ charge-trapping layer and the perovskite layer, we fabricated control devices without the HfO_2_ layer. As shown in Figs S28 and S29, these control devices exhibit virtually no typical synaptic behaviors, confirming that the HfO_2_ layer is the primary origin of synaptic plasticity and thus enables high synaptic fidelity. Under paired optical pulses, the device exhibits a clear paired-pulse facilitation (PPF) effect, a hallmark of short-term synaptic plasticity (Fig. 5b, and Figs S30 and S31). The PPF index decreases monotonically with increasing pulse interval and can be well fitted by a double-exponential decay, indicating the coexistence of fast and slow relaxation processes. The temporal evolution of the PPF index follows [47,48]:


(4) \begin{eqnarray*}{\mathrm{PPF}} = {C}_1 {\rm exp}\left( { - \frac{{{\mathrm{\Delta }}t}}{{{\tau }_1}}} \right)\ + \ {C}_2 {\rm exp}\left( { - \frac{{{\mathrm{\Delta }}t}}{{{\tau }_2}}} \right),\end{eqnarray*}

where *C*_1_ and *C*_2_ denote the initial facilitation amplitudes, and *τ*_1_ and *τ*_2_ correspond to fast and slow relaxation time constants, respectively, demonstrating the potential of PPF in visual systems. These dynamics are attributed to delayed recombination and gradual release of carriers trapped at surface and interfacial states. Beyond short-term plasticity, the synaptic response can be continuously tuned by the temporal profile of the optical stimulus. Increasing either pulse width or pulse frequency produces a monotonic increase in excitatory postsynaptic current (EPSC) (Fig. 5c and d, and Fig. S32), consistent with cumulative carrier trapping and incomplete relaxation between successive pulses. Under the operating conditions of a bias voltage of 0.01 V, a pulse width of 0.1 s and an optical power density of 0.75 mW cm^−2^ (Fig. S33), the minimum energy consumption was calculated to be 19 fJ according to the equation (*E* = *I* × *V* × *T*). This value indicates an ultralow single-event energy consumption for our device, which is significantly lower than or comparable to those of previously reported perovskite memristors (Table S3). Such pulse-dependent behavior closely resembles learning processes in biological synapses.

**Figure 5.**
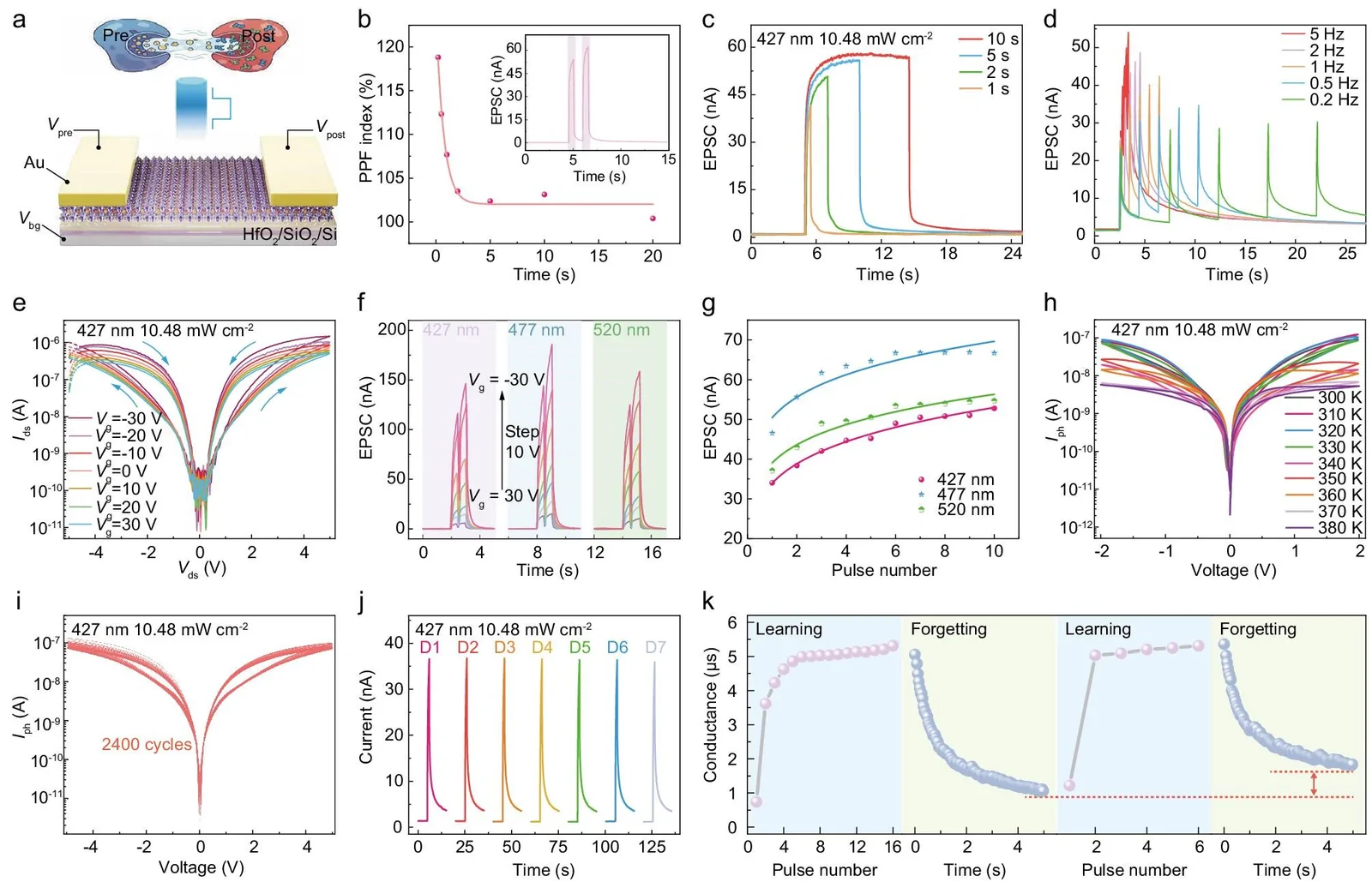
(a) Schematic illustration of the photosynapse device. (b) PPF ratio as a function of pulse interval for 427 nm optical pulses (10.48 mW cm^−2^, 0.5 s width); the solid curve represents a double-exponential fit. (c) Pulse width and (d) pulse frequency. (e) *I*–*V* curves under different back-gate voltages. (f) Photoresponses under different back-gate voltages (0.5 s width). (g) Synaptic responses to optical stimuli with different wavelengths and pulse numbers. (h) Switching behavior under elevated temperatures. (i) Endurance performance over repeated optical stimulation cycles. (j) Photoresponse stability of device coated with PMMA after storage in ambient air for 7 days (0.5 s width). (k) Learning-forgetting-relearning behavior emulated by the device.

Electrical gating provides an additional handle to modulate synaptic weight. As the back-gate voltage is swept toward more negative values, both the switching window and EPSC amplitude increase systematically (Fig. 5e and f, and Fig. S34), enabling programmable adjustment of synaptic response without altering the optical stimulus conditions [49]. Wavelength-dependent measurements further show that synaptic potentiation correlates strongly with the intrinsic absorption profile of the alloy (Fig. 5g). Optical excitation at 477 nm produces the strongest EPSC response, followed by 520 and 427 nm illumination, consistent with the spectral responsivity of the photodetector devices. The EPSC also increases monotonically with pulse number, enabling gradual and analog-like weight updates that are essential for neuromorphic learning.

Importantly, the synaptic behavior remains robust under thermal stress. Although the peak current decreases gradually with increasing temperature, the switching characteristics and plasticity behavior are preserved up to 380 K (Fig. 5h). This thermal tolerance is consistent with the enhanced lattice stability and suppressed ion migration observed for Cs_0.81_Rb_0.19_PbBr_3_. Device reliability was further evaluated through endurance and shelf-life tests. After >2400 optical stimulation cycles, the device shows negligible degradation in switching behavior (Fig. 5i), exhibits highly repeatable EPSC responses under 375-pulse continuous stimulation (Fig. S35) and retains stable synaptic responses after storage in ambient air for seven days (Fig. 5j). Finally, the device is capable of emulating higher-order learning behavior, including a learning-forgetting-relearning process (Fig. 5k), where fewer stimuli are required to restore synaptic weights after partial memory decay. The reproducibility of these behaviors highlights that intrinsic materials’ stability directly translates into synaptic fidelity, providing a practical foundation for robust neuromorphic optoelectronic hardware. Collectively, the device exhibits stable and tunable synaptic plasticity across a wide range of optical conditions, including pulse-width dependence, frequency dependence, power dependence (Fig. S36), wavelength dependence and both short-term and long-term plasticity. Building on these device-level characteristics, we next integrate the optoelectronic synapse into neural network models to evaluate system-level in-sensor computing performance.

To examine whether the stability advantages at the materials and device levels can translate into reliable system-level computation, we implemented an SNN for visual classification using experimentally measured characteristics of the Cs_0.81_Rb_0.19_PbBr_3_ photosynaptic devices (Fig. 6a). Rather than pursuing the highest possible algorithmic accuracy, our goal is to assess whether stable perovskite-based synapses can support consistent spiking computation without requiring frequent recalibration. The network adopts a Residual Network (ResNet)-inspired SNN architecture, where convolutional layers extract spatial features and leaky integrate-and-fire (LIF) neurons encode temporal information through spike trains. By means of simulation, the electrical conductance of the memristor is mapped to the weights of the network [50]. We first evaluated the trainability of the device-informed SNN on the FashionMNIST dataset (Fig. S37). As shown in Fig. 6b, both training and inference accuracies increase rapidly during early epochs and converge to stable values close to 90%, with only a small gap between training and test performance. The corresponding confusion matrix (Fig. 6c) exhibits strong diagonal dominance, indicating reliable discrimination among classes. Similar convergence behavior is observed for the MNIST dataset (Figs S38 and S39), suggesting that the network generalizes well and does not suffer from noticeable overfitting. In addition, firing statistics of LIF neurons remain well regulated across layers throughout training (Fig. 6d, and Figs S41, S42 and S44), avoiding both excessive spiking activity and neuronal silence. These results indicate that the ResNet-SNN architecture provides stable temporal dynamics compatible with device-level implementation. It should be emphasized that the fire-detection task presented here serves as a proof-of-concept system-level validation of the compatibility between the stable Cs_0.81_Rb_0.19_PbBr_3_ photosynaptic device and the ResNet-SNN computational framework, rather than a fully deployed fire-detection system. The objective is to evaluate whether experimentally measured synaptic dynamics can support stable neuromorphic inference. Practical deployment would require further development of large-scale device arrays, peripheral circuits, real-time image acquisition, extended environmental reliability tests and system-level power evaluation.

**Figure 6.**
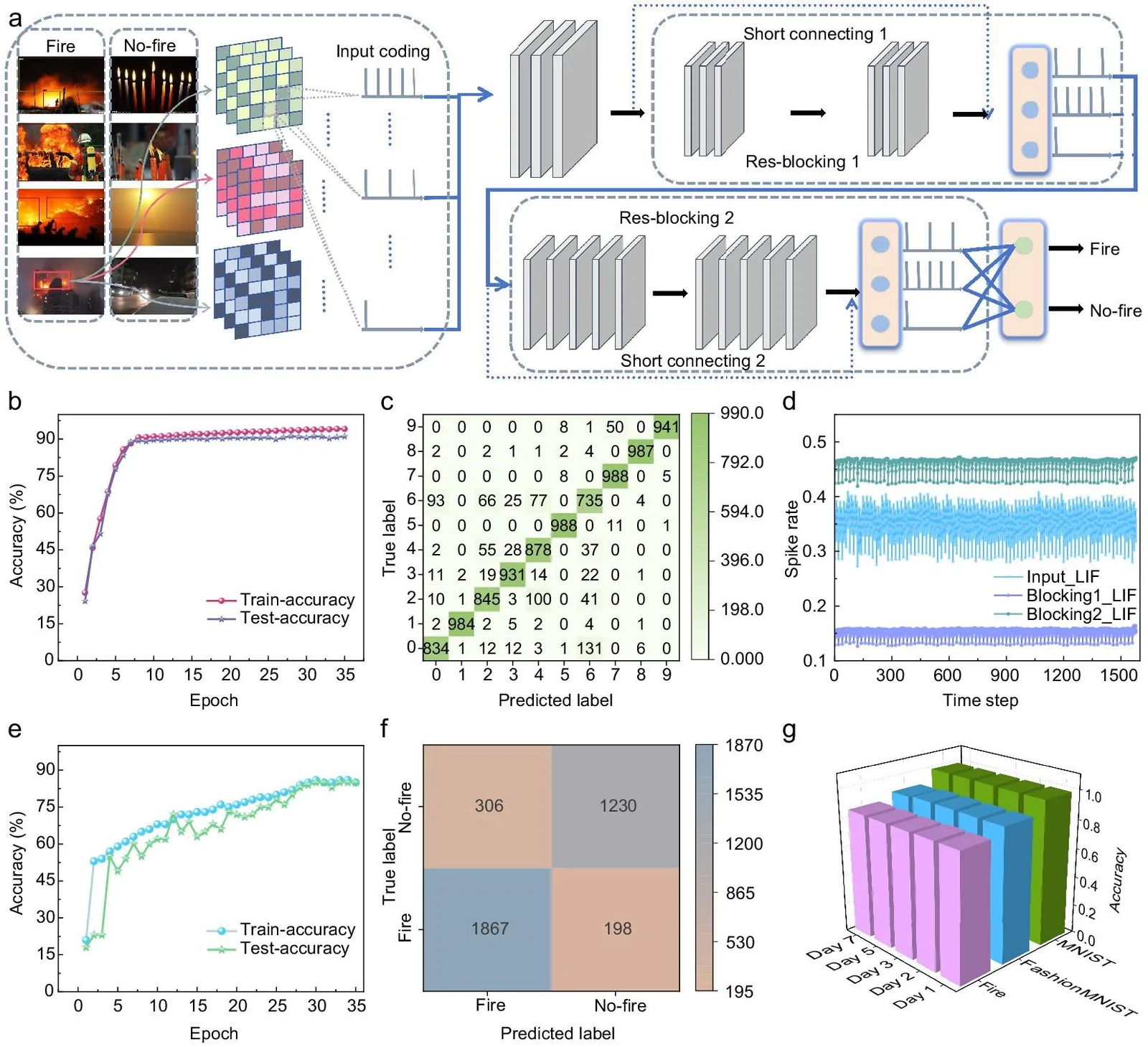
(a) Architecture of the ResNet-SNN used for image classification. (b) Training and inference accuracy on the FashionMNIST dataset. (c) Confusion matrix after final training on FashionMNIST. (d) Firing frequency distribution of LIF neurons. (e) Training and inference accuracy on the fire dataset. (f) Confusion matrix for fire detection. (g) Evolution of inference accuracy over extended operation time.

Having established network convergence on benchmark datasets, we next evaluated performance on a more application-relevant fire detection task based on RGB image sequences. Compared with digit datasets, fire scenes exhibit substantially higher complexity due to background clutter, illumination variation and diverse flame morphologies. Despite these challenges, the device-informed SNN reaches an inference accuracy of ∼85% after ∼30 epochs (Fig. 6e). The confusion matrix (Fig. 6f) shows that 90.41% of fire images (2065 samples) are correctly classified, while 19.93% of non-fire images (1536 samples) are misclassified as fire, demonstrating balanced classification behavior under realistic visual complexity.

Long-term inference stability is particularly critical for safety-related deployment. To evaluate temporal robustness, we monitored fire-recognition accuracy over an extended period. As shown in Fig. 6g, the inference accuracy varies by <1% over 1 week, consistent with the suppressed conductance drift observed in Cs_0.81_Rb_0.19_PbBr_3_ synaptic devices (Fig. S46). This result indicates that the stability engineered at the materials level can directly propagate to system-level neuromorphic computation, enabling consistent inference performance without retraining or recalibration. Beyond the demonstrated fire-detection task, the proposed stability-driven photosynaptic platform may be extended to broader application scenarios requiring reliable and energy-efficient visual perception. For autonomous driving systems, stable perovskite photosynapses could be used for event-driven hazard perception, early fire or smoke warning and low-latency optical signal preprocessing under complex illumination conditions. For robot vision, the integration of light sensing, memory and synaptic weight modulation within a single device could enable adaptive environmental recognition and edge visual intelligence with reduced data-transfer overhead. The long-term stability demonstrated in this work is particularly important for these application scenarios, because practical neuromorphic vision systems require not only high instantaneous accuracy but also stable inference without frequent recalibration or retraining.

## CONCLUSIONS

In summary, we demonstrate that compositional engineering of 2D inorganic perovskites provides a practical route toward stable neuromorphic optoelectronic systems. By continuously tuning the A-site composition in Cs*_x_*Rb_1−_*_x_*PbBr_3_ nanosheets, we identify a narrow intermediate regime in which lattice strain is minimized and defect-related degradation is suppressed. The optimized Cs_0.81_Rb_0.19_PbBr_3_ composition exhibits enhanced resistance to moisture, thermal stress and ion migration, enabling stable photodetection and reproducible synaptic plasticity. Beyond device demonstrations, we further show that experimentally measured synaptic responses can be directly incorporated into SNNs to support system-level neuromorphic vision. The resulting ResNet-SNN achieves stable training convergence and enables real-time fire detection with ∼85% accuracy, while maintaining performance fluctuations below 1% over 1 week of operation. These results establish a clear materials-device-algorithm connection, highlighting that intrinsic material stability is a key requirement for reliable in-sensor computing. More broadly, this work suggests that stability-driven compositional design offers an effective strategy to bridge emerging perovskite materials with practical neuromorphic hardware, where long-term operational consistency is as important as instantaneous performance.

## Supplementary Material

nwag453_Supplemental_File
