# Supplementary material for: Composition-engineered intrinsic stability in 2D perovskite optoelectronic synapses for neuromorphic vision
Source: Natl Sci Rev. 2026 Jul 22;13(19):nwag453. doi: 10.1093/nsr/nwag453 (PMC13717458; doi:10.1093/nsr/nwag453)
Supplement: Supplementary file 1 [file nwag453_supplemental_file.pdf]

Supporting Information

Composition-engineered intrinsic stability in 2D perovskite opto-electronic synapses for neuromorphic vision

Honglai Li[1,†,*], Wei Li[1,†], Herong Sheng[4,6,†], Changhao Liu[1], Wenjun Yang[1], Jinpeng Zhao[5], Shida Feng[1], Biyuan Zheng[3], Chenglin He[3], Jiahuan Ren[1], Liang Ma[5], Tingzhao Fu[3], Chun Zhao[4], Yuxiao Fang[4,*], Weihao Zheng[3,*], Dong Li[2], Zhihong Zhu[3] and An-lian Pan[2,7,*]

[1]College of Physics Science and Technology, Hebei University, Baoding 071002, China;

[2]Hunan Institute of Optoelectronic Integration, College of Materials Science and Engineering, Hunan University, Changsha 410082, China;

[3]College of Advanced Interdisciplinary Studies & Hunan Provincial Key Laboratory of Novel Nano-Optoelectronic Information Materials and Devices, National University of Defense Technology, Changsha 410073, China;

[4]School of Advanced Technology, Xi'an Jiaotong-Liverpool University, Suzhou 215123, China;

[5]College of Physics and Electronic Engineering, Hengyang Normal University, Hengyang 421008, China;

[6]Department of Electrical Engineering and Electronics, University of Liverpool, Liverpool L69 3BX, UK and [7]School of Physics and Electronics, Hunan Normal University, Changsha 410081, China

***Corresponding authors**. E-mails: lihonglai@hnu.edu.cn; yuxiao.fang02@xjtlu.edu.cn; zhengweihao@nudt.edu.cn; anlian.pan@hnu.edu.cn

[†]Equally contributed to this work.

Materials and Methods

Growth of 2D $Cs_xRb_{1-x}PbBr_3$ Nanosheets

Large-scale 2D $Cs_xRb_{1-x}PbBr_3$ ($0 \leq x \leq 1$) single-crystalline nanosheets were synthesized via a simple vapor-phase deposition in a horizontal tube furnace. Stoichiometric amounts of CsBr (Aladdin, 99.99%), RbBr (Aladdin, 99.99%), and $PbBr_2$ (Aladdin, 99.99%) powders were mixed at target molar ratios (x = 0, 0.2, 0.3, 0.4, 0.6, 0.7, 0.8, 1) and thoroughly ground to ensure microscopic homogeneity. The precursor powder was loaded into a clean quartz boat and positioned at the center of the heating zone, while a freshly cleaved white mica substrate was placed 1-2 cm downstream from the boat. Before growth, the quartz tube (1 m in length, 1 inch in diameter) was evacuated to 0.1 Torr and backfilled with high-purity Ar gas three times to eliminate residual air. Subsequently, the furnace was ramped from ambient temperature to the target growth temperature 500 °C for $CsPbBr_3$ and 350 °C for $RbPbBr_3$) over 20 min under an Ar flow rate of 30-80 SCCM (30 SCCM for $CsPbBr_3$ and 100 SCCM for $RbPbBr_3$). The growth was maintained for 15-60 min (15 min for $CsPbBr_3$ and 60 min for $RbPbBr_3$) at a stable pressure of approximately 30 Torr. Detailed growth parameters for 2D $CsxRb_{1-x}PbBr_3$ nanosheets with different compositions, including specific temperatures, flow rates, and durations, are summarized in Table S2. Finally, the system was naturally cooled to room temperature, and the mica substrate was retrieved after the system returned to atmospheric pressure.

Transfer method of $Cs_xRb_{1-x}PbBr_3$ Nanosheets

To preserve the structural integrity and accommodate the environmental sensitivity of the $Cs_xRb_{1-x}PbBr_3$ nanosheets, a polymethylmethacrylate (PMMA)-assisted dry transfer method was employed. To ensure mechanical stability during handling, a PMMA film was spin-coated onto the nanosheet-decorated mica substrate at 4000 rpm, followed by baking at 100°C for 3 min on a hot plate. The PMMA/nanosheets composite was then delaminated from the mica surface using thermal release tape (TRT). To release the PMMA/nanosheet membrane, the TRT was heated at 100°C for 5 min, triggering the tape's self-release mechanism. The resulting film was subsequently placed onto the target substrate. Finally, the sacrificial PMMA layer was removed by drop-casting chlorobenzene (Aladdin, 99.5%) onto the film. After 5 min, the dissolved PMMA and residual solvent were removed by a gentle nitrogen stream, successfully yielding clean $Cs_xRb_{1-x}PbBr_3$ nanosheets on the target surface.

Structure and Composition Characterization

The morphology and dimensions of the $Cs_xRb_{1-x}PbBr_3$ nanosheets were characterized using OM (Olympus BX51M) and SEM (Zeiss Sigma-HD). Elemental mapping and compositional analyses were performed via EDS integrated with the SEM. AFM (Oxford Instruments, MFP-3D) was employed to determine the thickness and surface roughness. The crystal structure and phase purity were investigated by XRD (Bruker AXS, D8 ADVANCE), while the chemical states were scrutinized using XPS (Thermo Fisher Scientific, ESCALAB 250Xi). Atomic-resolution STEM and EDS mapping were performed on a

double aberration-corrected FEI Titan Cubed G2 60-300 at 300 kV (convergence angle: 21.4 mrad; probe current: 60 pA; integration time: 8 s).

Optical Measurements

Steady-state PL spectra and TRPL measurements of $Cs_xRb_{1-x}PbBr_3$ nanosheets were performed using a confocal microscope system (WITec, alpha-300). A mode-locked Ti: sapphire laser (Tsunami) operating at 800 nm (pulse width: 80 fs, repetition rate: 80 MHz) was coupled to a β-barium borate (BBO) crystal to generate second-harmonic radiation at 400 nm, which served as the excitation source. For lasing experiments, the same confocal microscope platform was utilized: the 800 nm laser was amplified via a regenerative amplifier (Spitfire Ace 100, 1 kHz) and subsequently frequency-doubled using a BBO crystal to produce 400 nm excitation light (1 kHz repetition rate). An Olympus 20× objective lens focused the excitation beam into a uniform spot (diameter: 200 μm) on the sample surface. PL and lasing signals were collected and analyzed using a spectrometer. TRPL measurements were carried out by redirecting the emission signals to a streak camera (C10910, Hamamatsu) via silver (Ag) mirrors.

Preparation of HfO_2 Thin Films

SiO_2/Si wafers were employed as substrates. The substrates were ultrasonically cleaned in anhydrous ethanol (99.9%) for 30 min at ambient temperature, followed by drying with a high-purity nitrogen stream to ensure a contaminant-free surface. A 20-nm-thick HfO_2 dielectric film was subsequently deposited via atomic layer deposition (ALD, MNT-S200-L3). The deposition was performed at 200°C using a pulse-and-purge cycle protocol, with a calibrated growth rate of 0.122 nm per cycle.

Device Fabrication

Au metal electrodes 80 nm thick were deposited on a sacrificial SiO_2/Si substrate first by standard photolithography and then by a high vacuum thermal evaporation process. The Au electrodes were then placed in a hexamethyldisilazane (Energy Chemical, 99%) solution, heated to 90°C and held for 60 min to increase adhesion to the Au and subsequently homogenized PMMA adhesive. Subsequently, PMMA was spin-coated onto the Au electrodes for the purpose of lifting them off. Using TRT, the Au/PMMA composite was delaminated from the sacrificial SiO_2/Si substrate. This stack was then precisely aligned and transferred onto the $Cs_xRb_{1-x}PbBr_3$ nanosheets (supported by mica or HfO_2/SiO_2/Si substrates) using a high-precision transfer platform. The assembly was heated to 100°C for 10 min to trigger the TRT release and ensure robust contact between the electrodes and the nanosheets. Finally, the device was immersed in chlorobenzene for 5 min to dissolve the sacrificial PMMA layer, leaving the gold electrodes successfully transferred onto the material surface with strong interfacial contact.

Optoelectronic Device Characterization

Comprehensive photoelectric tests were conducted using a B1500A semiconductor parameter analyzer coupled with a high-precision probe station in a shielded environment. Optical modulation was provided

by Ultra-Multi-Wavelength LED Sources (UMW-60) with calibrated power densities. All I-V measurements used a voltage sweep rate of 0.1 V/s. Transient photoresponse and synaptic tests used a data sampling interval of 10 μs for fast dynamics and 1 s for long-term stability monitoring. Temperature-dependent measurements were performed using a temperature-controlled probe stage with an accuracy of ±0.5K.

Design of SNNs

The input images are RGB images containing fire and non-fire scenes. To ensure a fair evaluation of the model, the dataset was divided into training and testing subsets at a ratio of 8:2, while maintaining a consistent fire/non-fire class distribution in both subsets. The training set was used exclusively for network parameter optimization, whereas the testing set remained independent throughout the entire training process and was used only for final performance evaluation. To improve the generalization capability of the model, data augmentation was applied only to the training images, including random horizontal flipping, random rotation, and brightness/contrast perturbation. No data augmentation was applied to the testing images, except for resizing and normalization. In SNNs, the output of the convolutional layer is converted into spike sequences. Ultimately, the spike frequency of neurons is used for classification. During training, input images pass through the convolutional layers and spike neuron layers of the ResNet network, extracting features and transmitting information layer by layer. LIF neurons determine whether to fire based on the cumulative discharge from the previous layer's output, which reaches the membrane potential threshold. ResNet consists of an input layer, convolutional layers, residual modules, and fully connected layers. Input images originate from real fire datasets. Key features are extracted through cascaded convolutional layers, while residual modules prevent gradient vanishing during training via residual connections. Finally, the extracted features are mapped to two categories via fully connected layers.

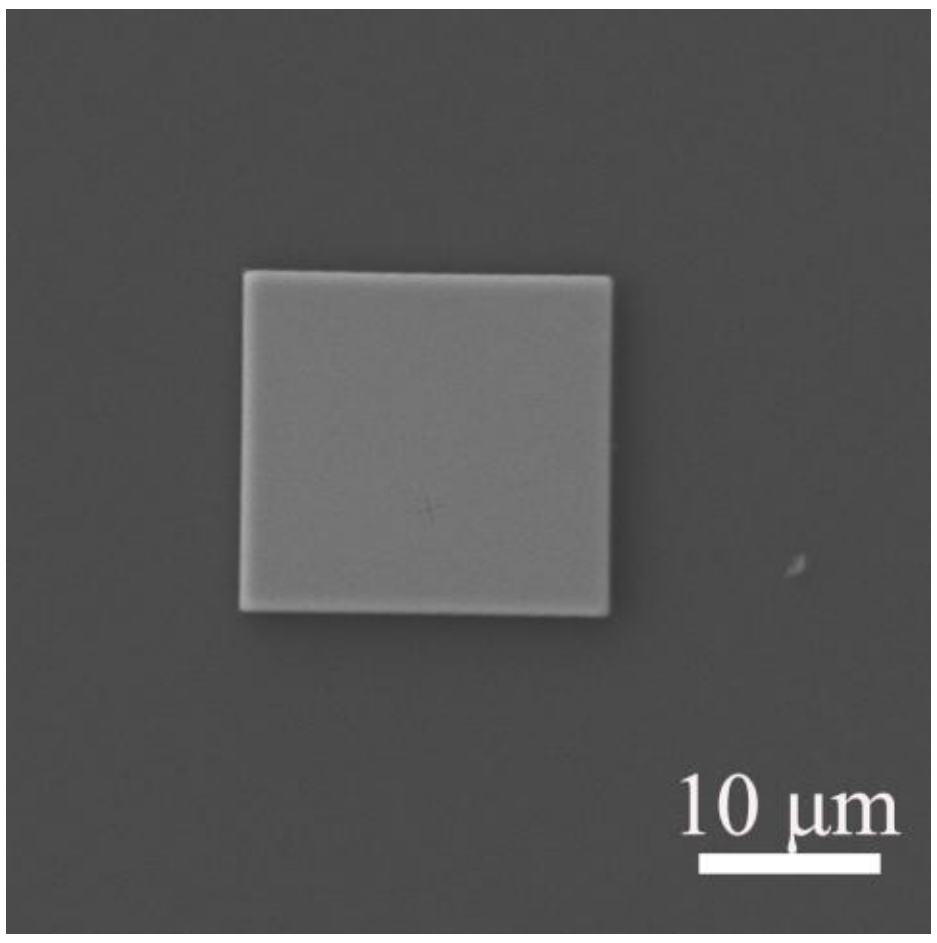

Figure S1. SEM image of a $Cs_xRb_{1-x}PbBr_3$ ($0 \le x \le 1$) nanosheet.

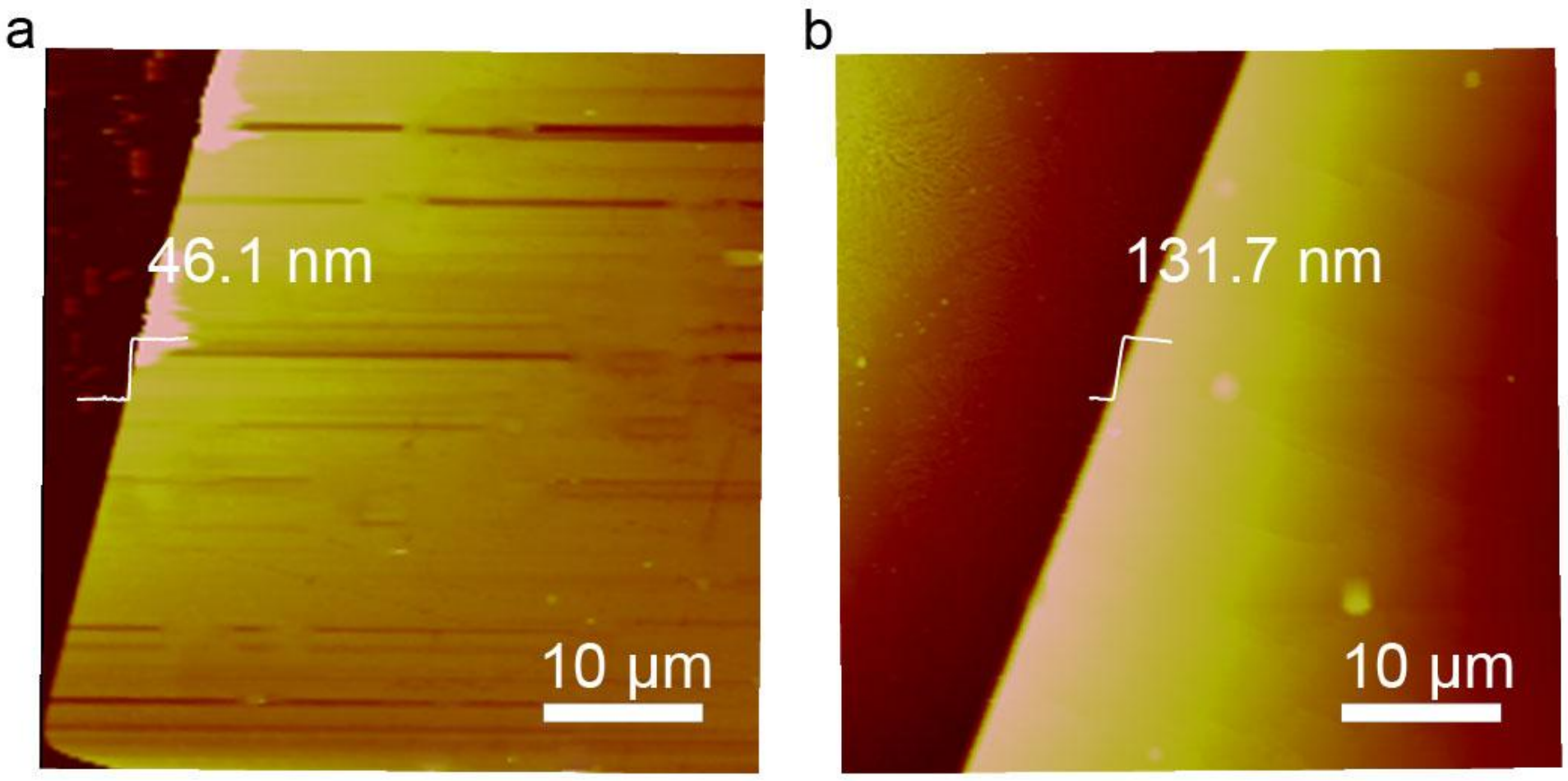

Figure S2. AFM morphologies of $Cs_xRb_{1-x}PbBr_3$ ($0 \leq x \leq 1$) nanosheets with various thicknesses.

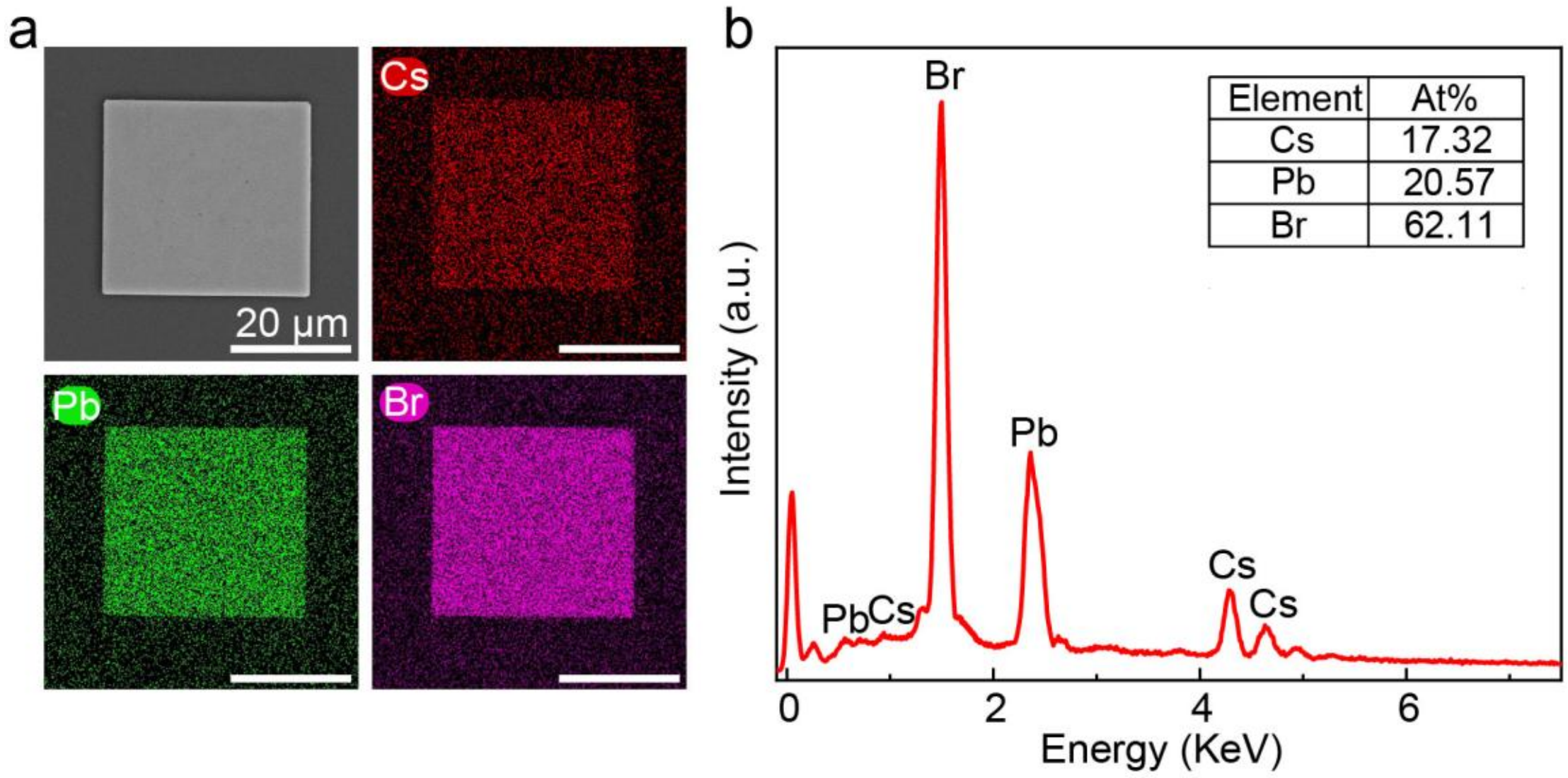

Figure S3. (a) SEM image and the corresponding EDS mapping of $CsPbBr_3$ nanosheet. (b) The corresponding EDS spectrum.

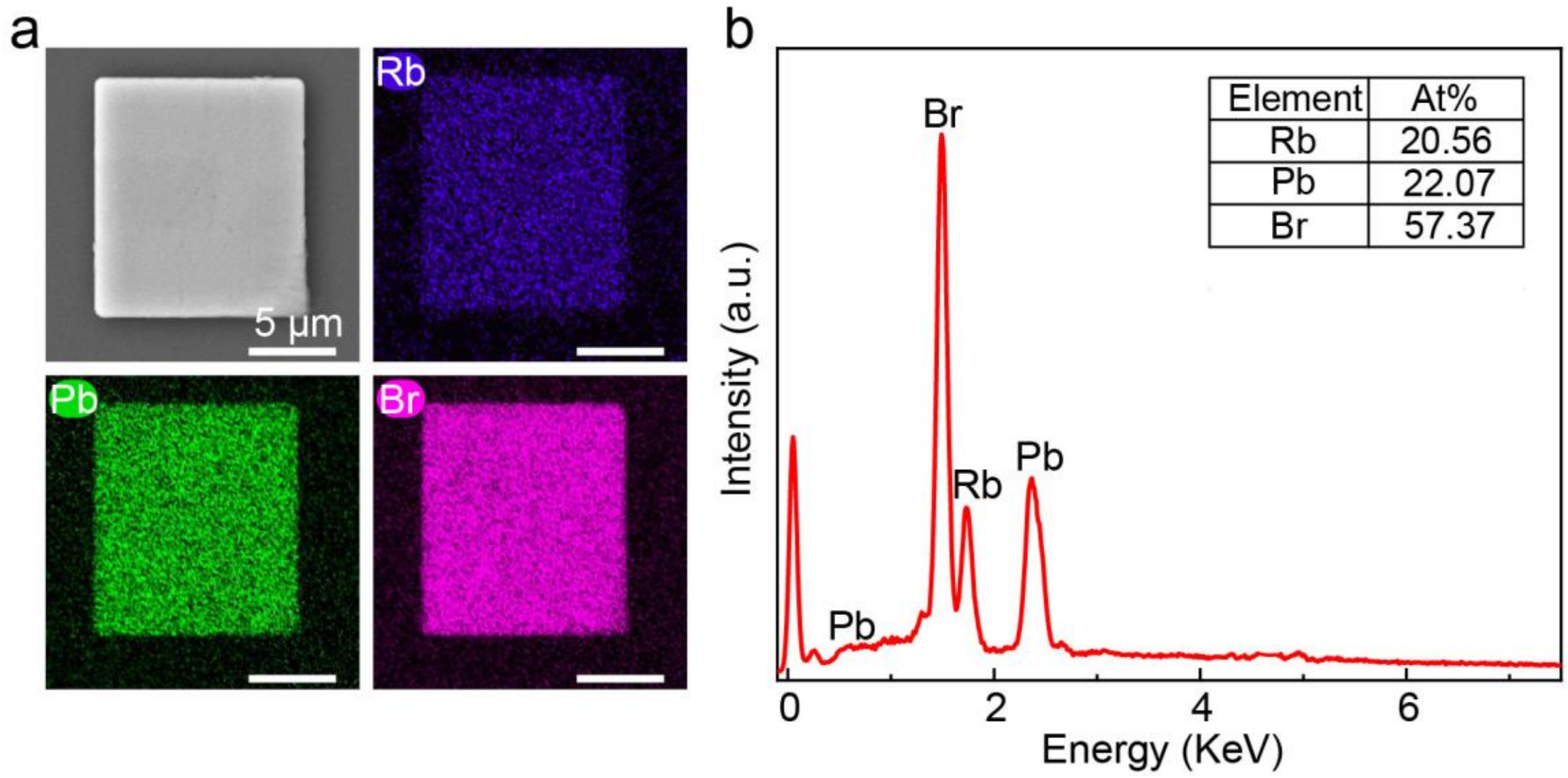

Figure S4. (a) SEM image and the corresponding EDS mapping of $RbPbBr_3$ nanosheet. (b) The corresponding EDS spectrum.

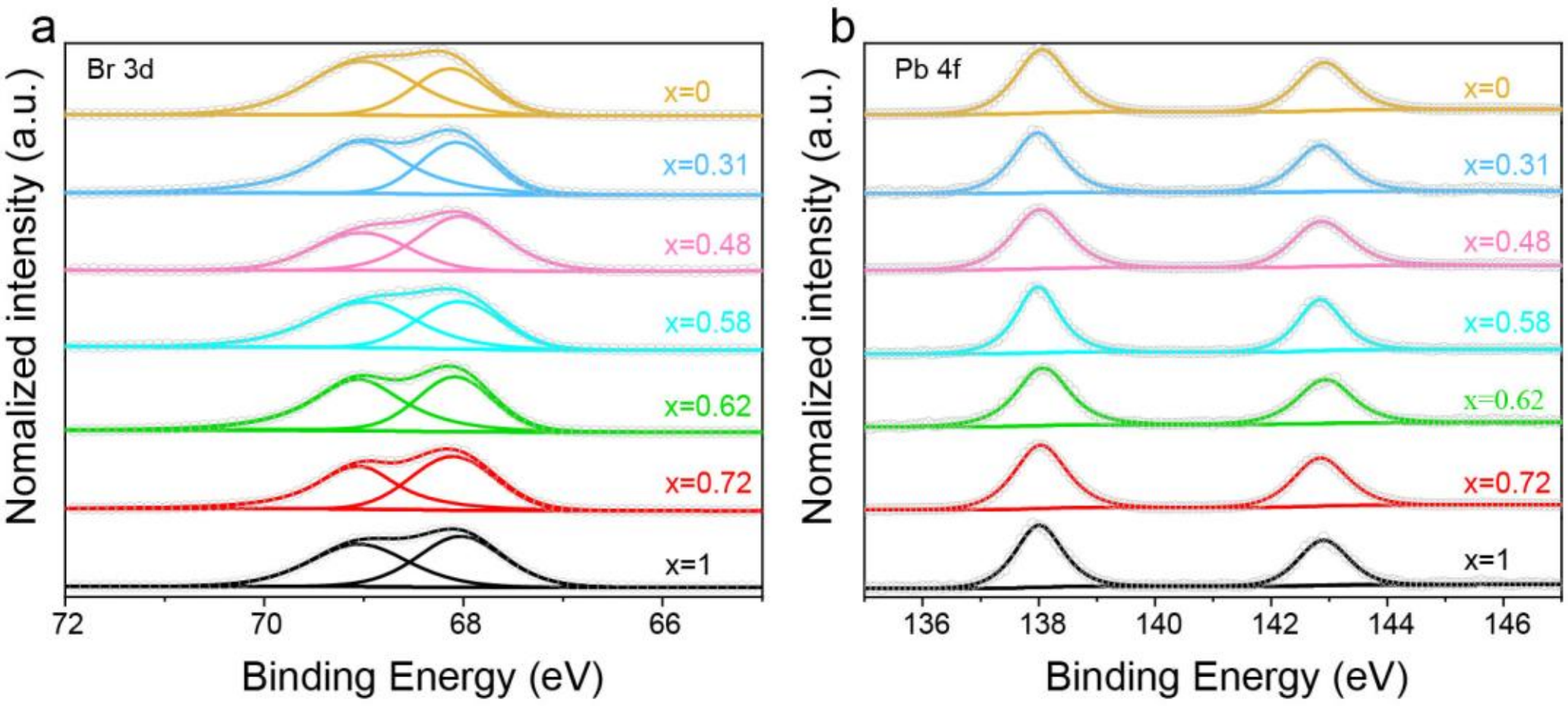

Figure S5. Br 3d and Pb 4f core level XPS spectra of $Cs_xRb_{1-x}PbBr_3$ ($0 \leq x \leq 1$) nanosheets with different Cs contents.

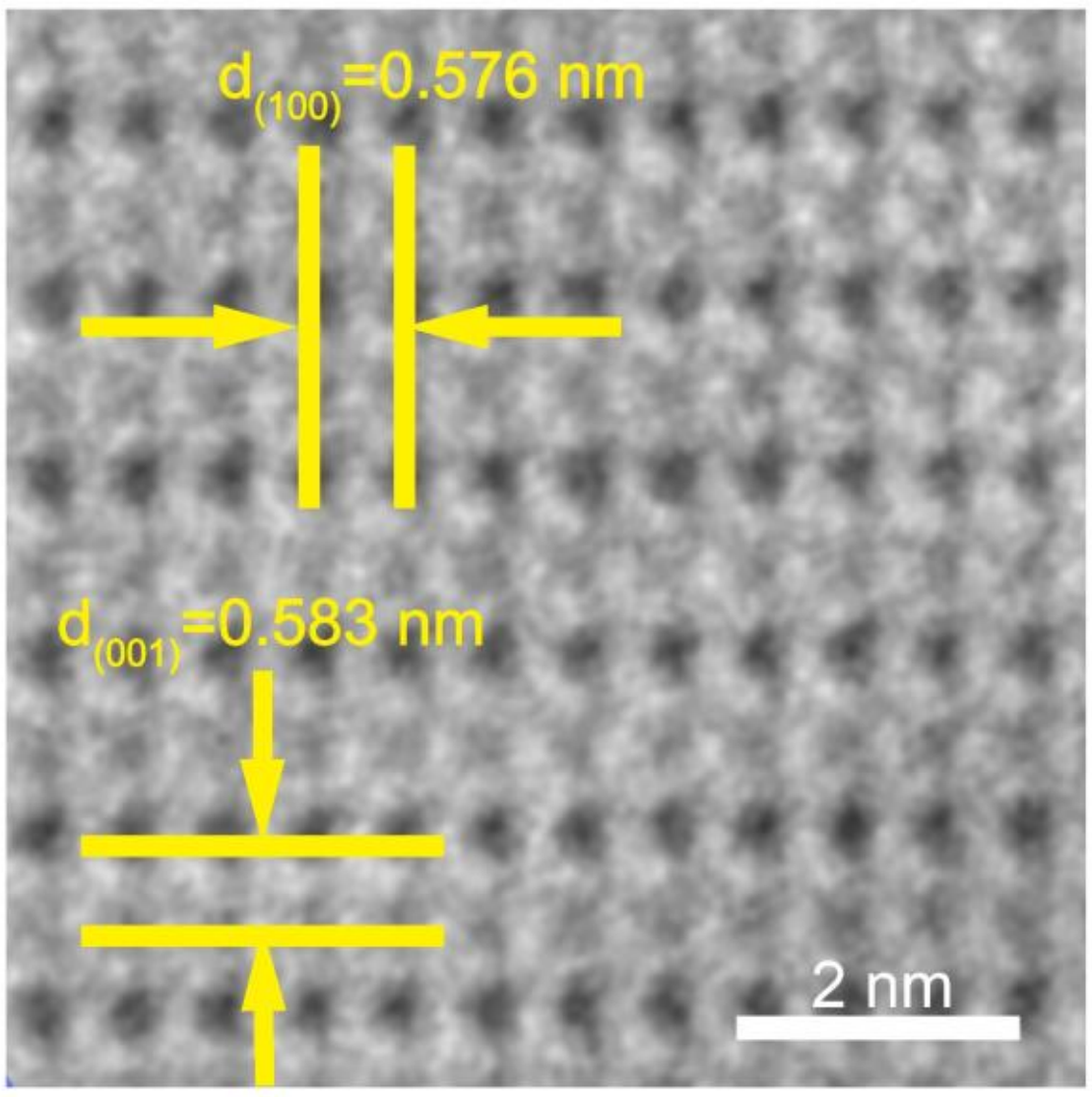

Figure S6. HRTEM images of the $Cs_{0.45}Rb_{0.55}PbBr_3$ nanosheet.

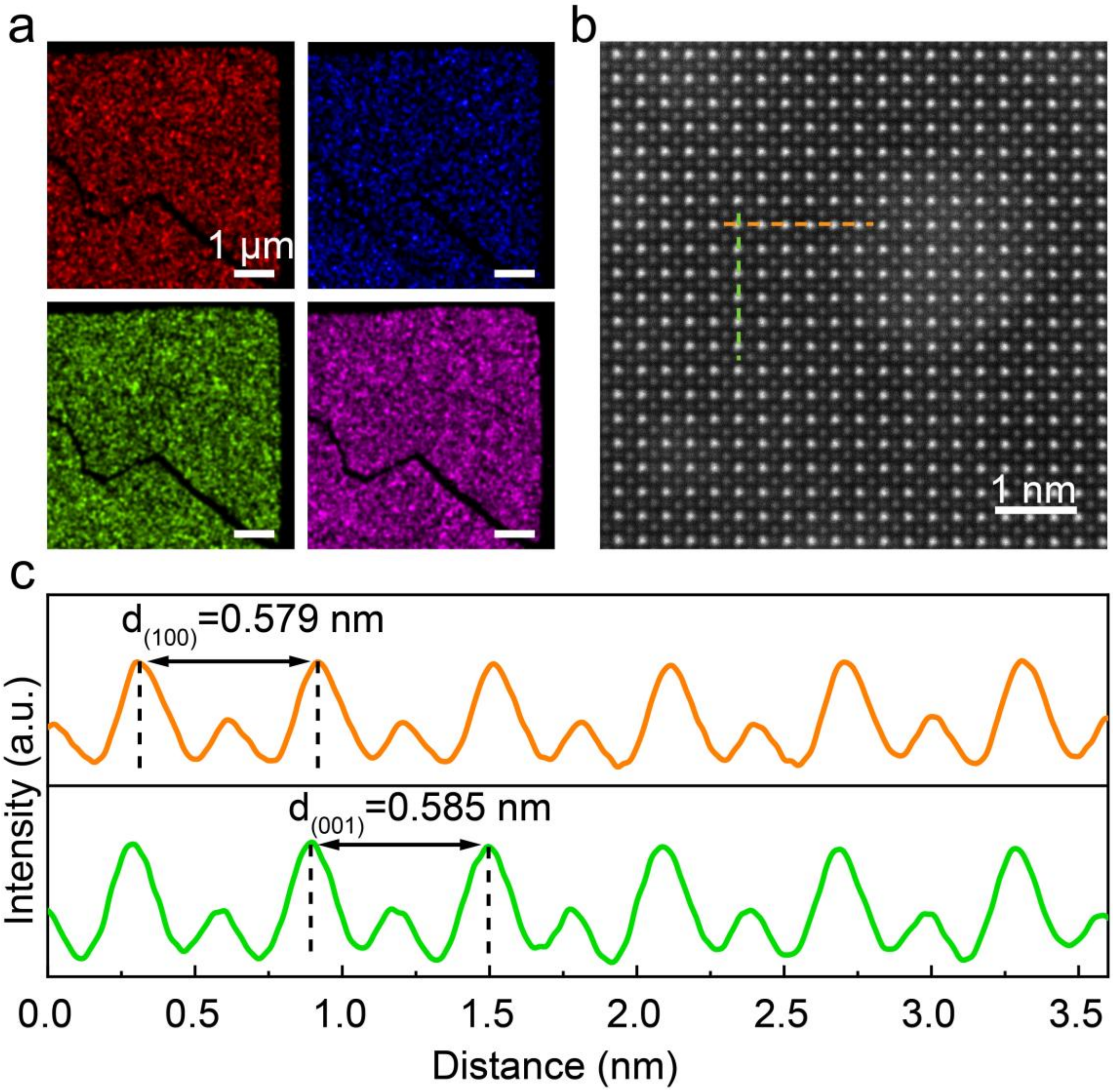

Figure S7. (a) EDS elemental mapping of $Cs_{0.72}Rb_{0.28}PbBr_3$ nanosheet. (b) Atomic-resolution HAADF-STEM image of $Cs_{0.72}Rb_{0.28}PbBr_3$ nanosheet. (c) Intensity profile extracted from the STEM image along the orange and green dashed lines in (b).

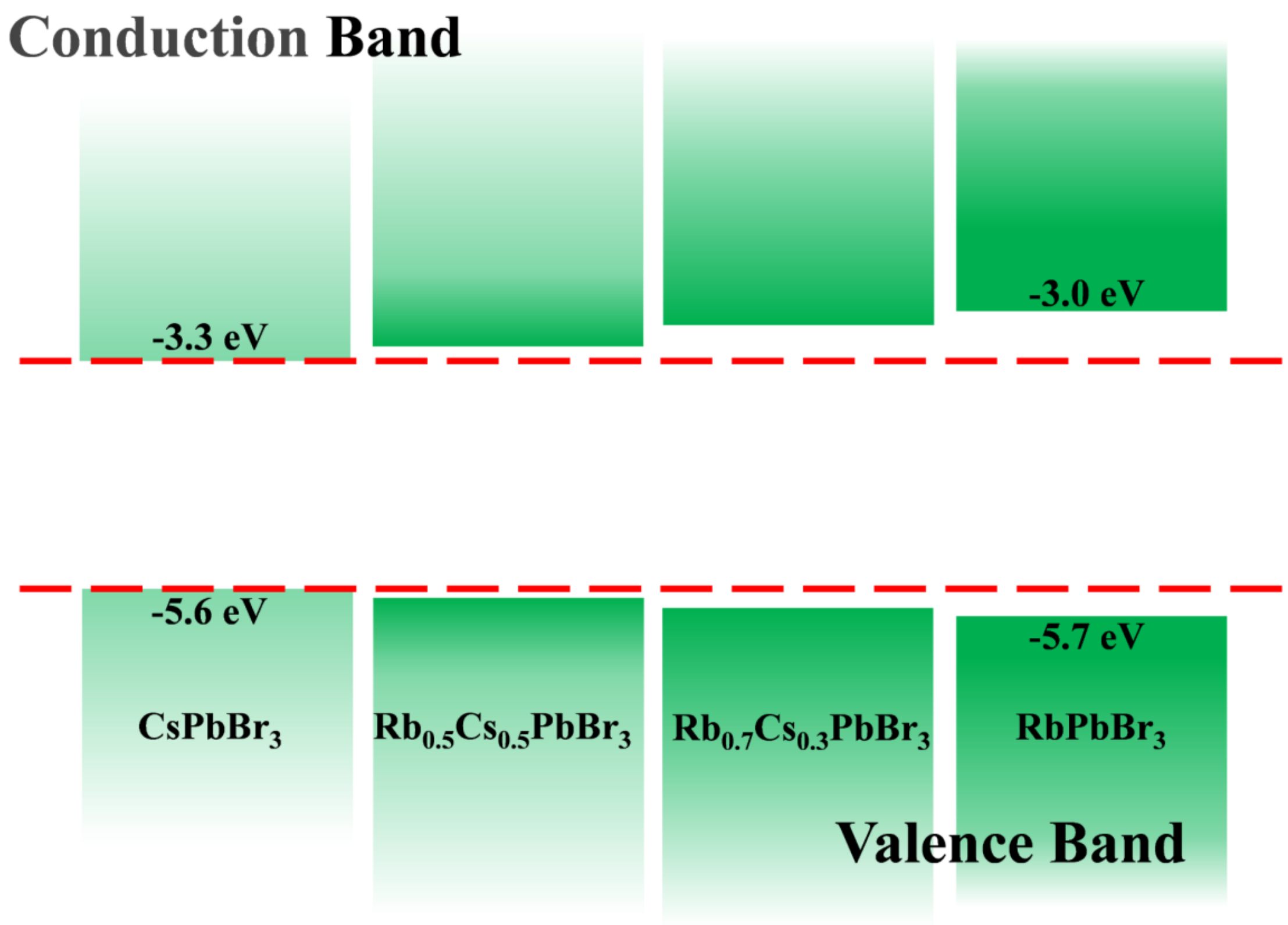

Figure S8. Absolute position of the band structure of $Cs_xRb_{1-x}PbBr_3$ ($0 \leq x \leq 1$) nanosheets.

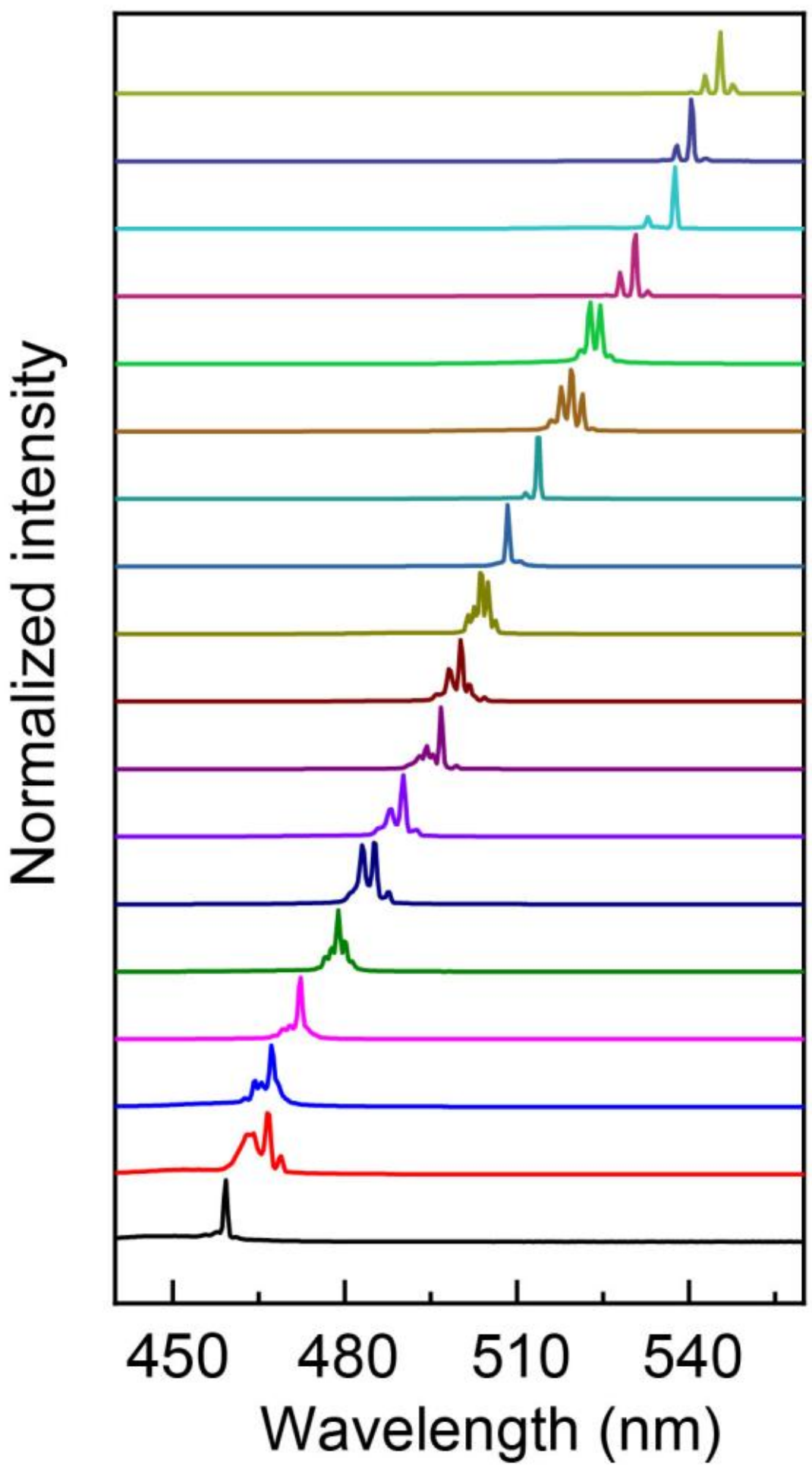

Figure S9 The emission spectra detected under different pump densities and wavelength of $Cs_xRb_{1-x}PbBr_3$ ($0 \leq x \leq 1$) nanosheets.

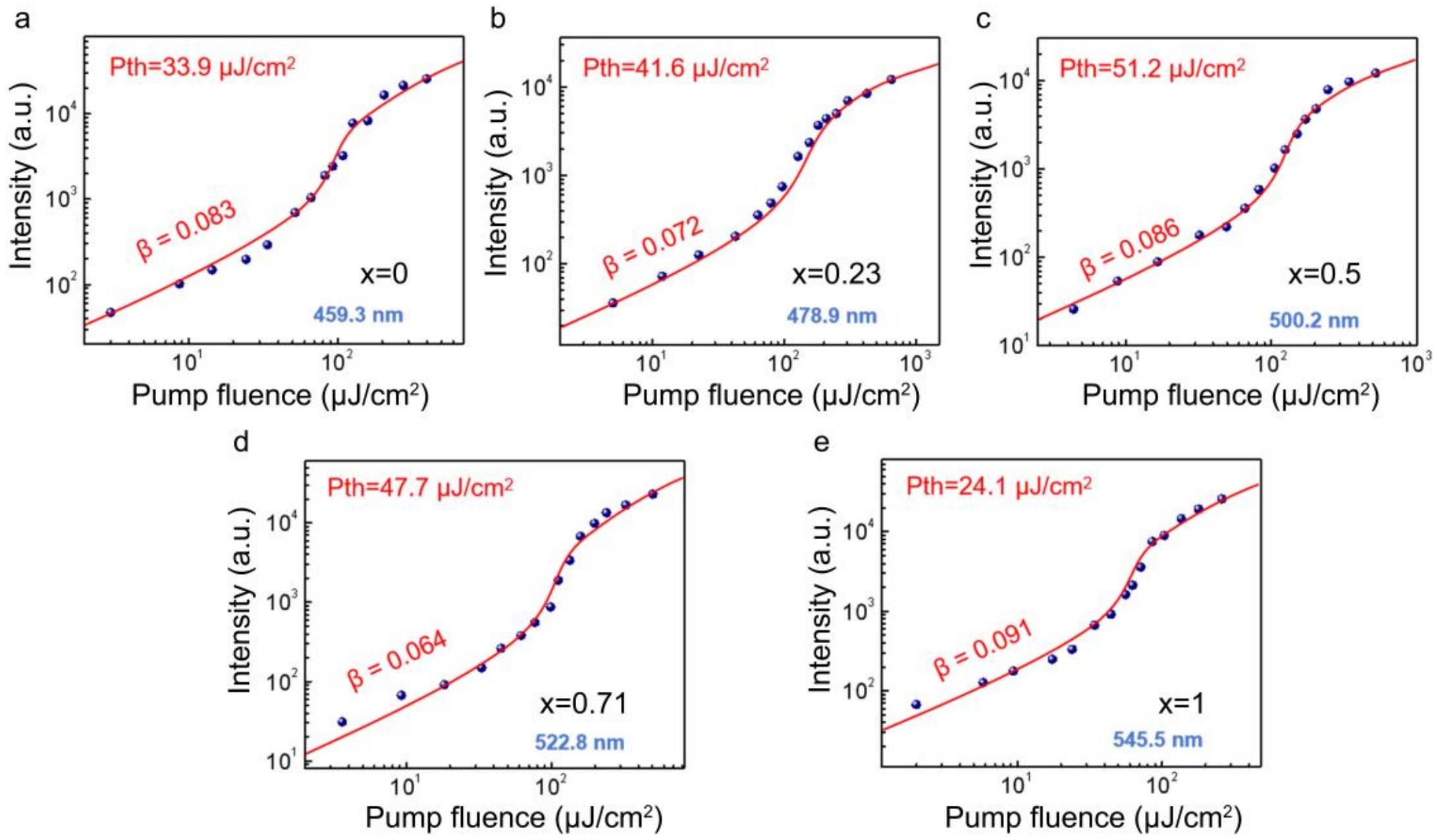

Figure S10. Numerical fitting of rate equations for $Cs_xRb_{1-x}PbBr_3$ ($0 \leq x \leq 1$) nanosheets lasing. The red lines denote the fitting results, while dark blue dots correspond to experimental data. The fitted spontaneous emission coupling coefficients β are provided, and the lines for $\beta = 1$ are shown in black for comparison.

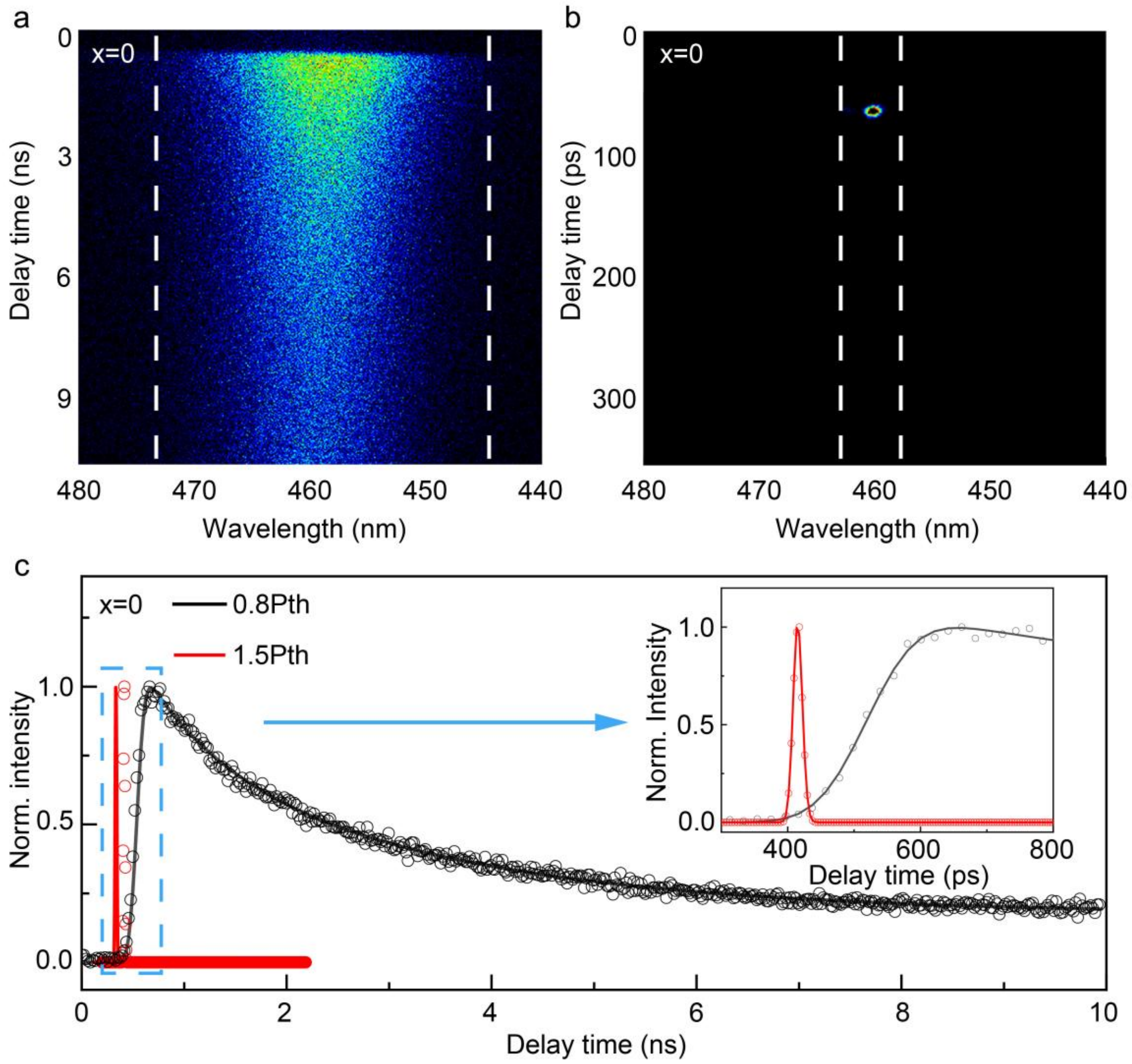

Figure S11. Streak-camera images of a $RbPbBr_3$ nanosheet recorded under excitation power of (a) 0.8 P_{th} (threshold excitation power) and (b) 1.5 P_{th}. (c) Typical PL-decay curve obtained at two different excitation powers (0.8, 1.5 P_{th}).

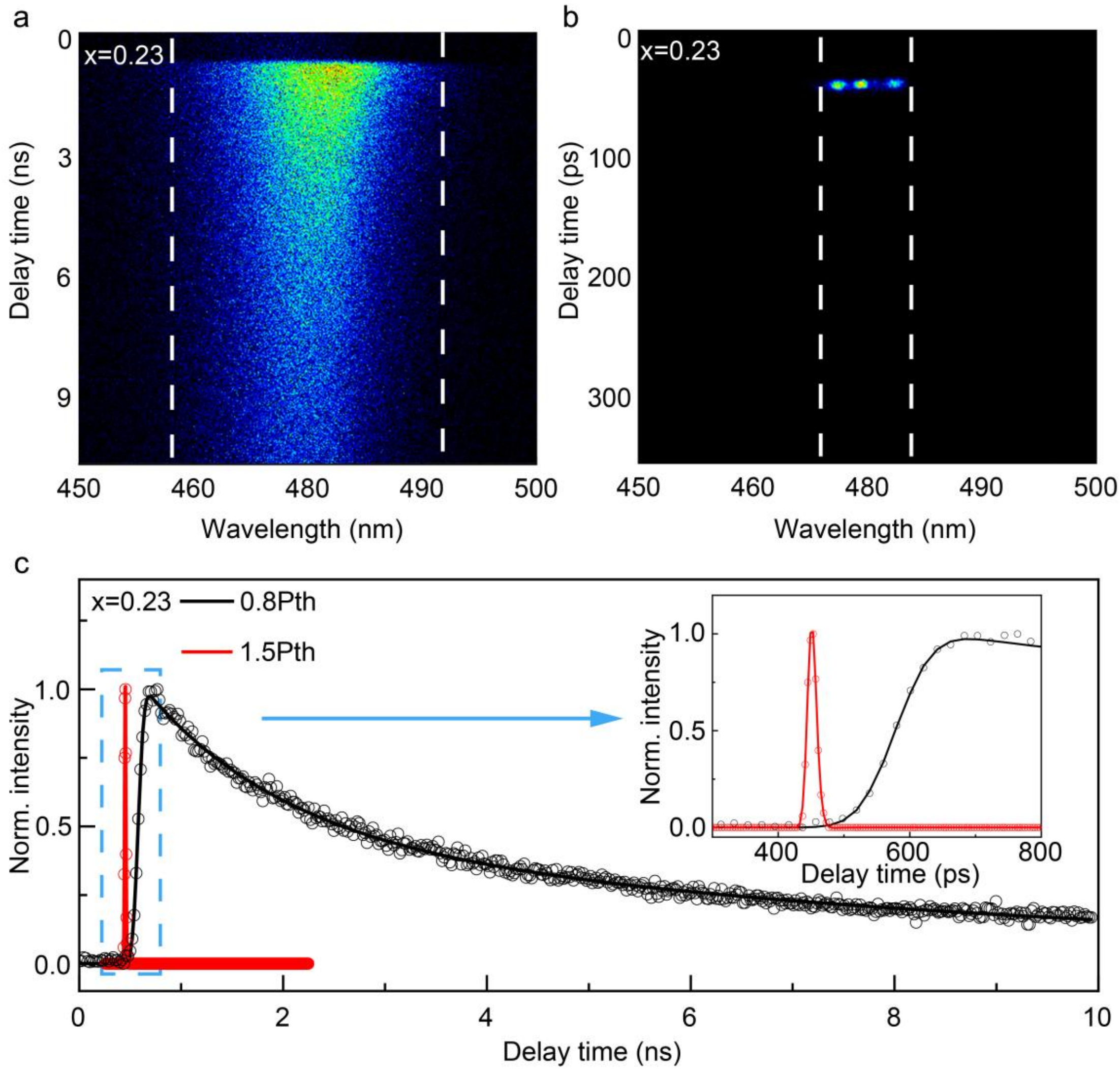

Figure S12. Streak-camera images of a $Cs_{0.23}Rb_{0.77}PbBr_3$ nanosheet recorded under excitation power of (a) 0.8 P_{th} (threshold excitation power) and (b) 1.5 P_{th}. (c) Typical PL-decay curve obtained at two different excitation powers (0.8, 1.5 P_{th}).

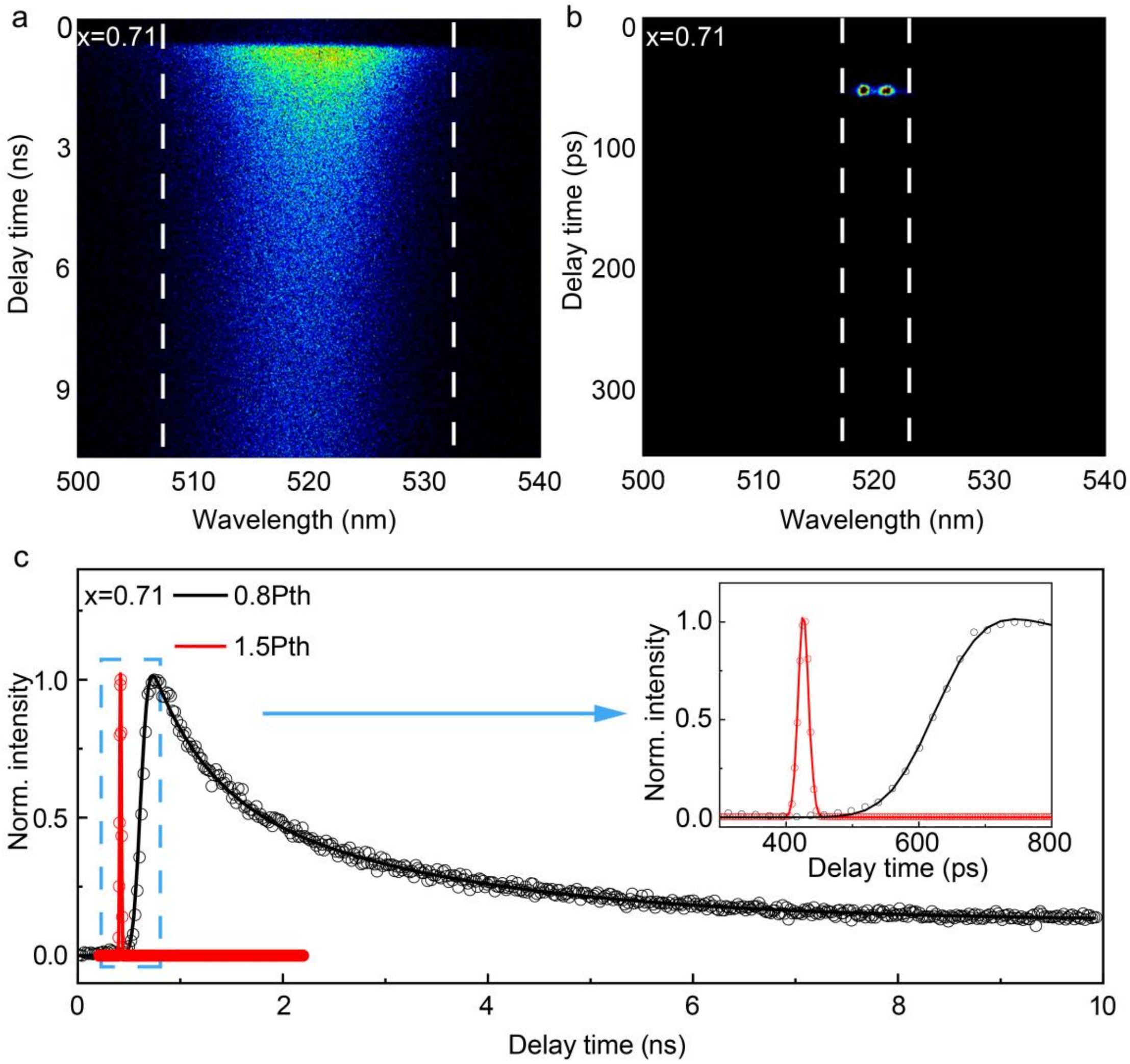

Figure S13. Streak-camera images of a $Cs_{0.71}Rb_{0.29}PbBr_3$ nanosheet recorded under excitation power of (a) 0.8 P_{th} (threshold excitation power) and (b) 1.5 P_{th}. (c) Typical PL-decay curve obtained at two different excitation powers (0.8, 1.5 P_{th}).

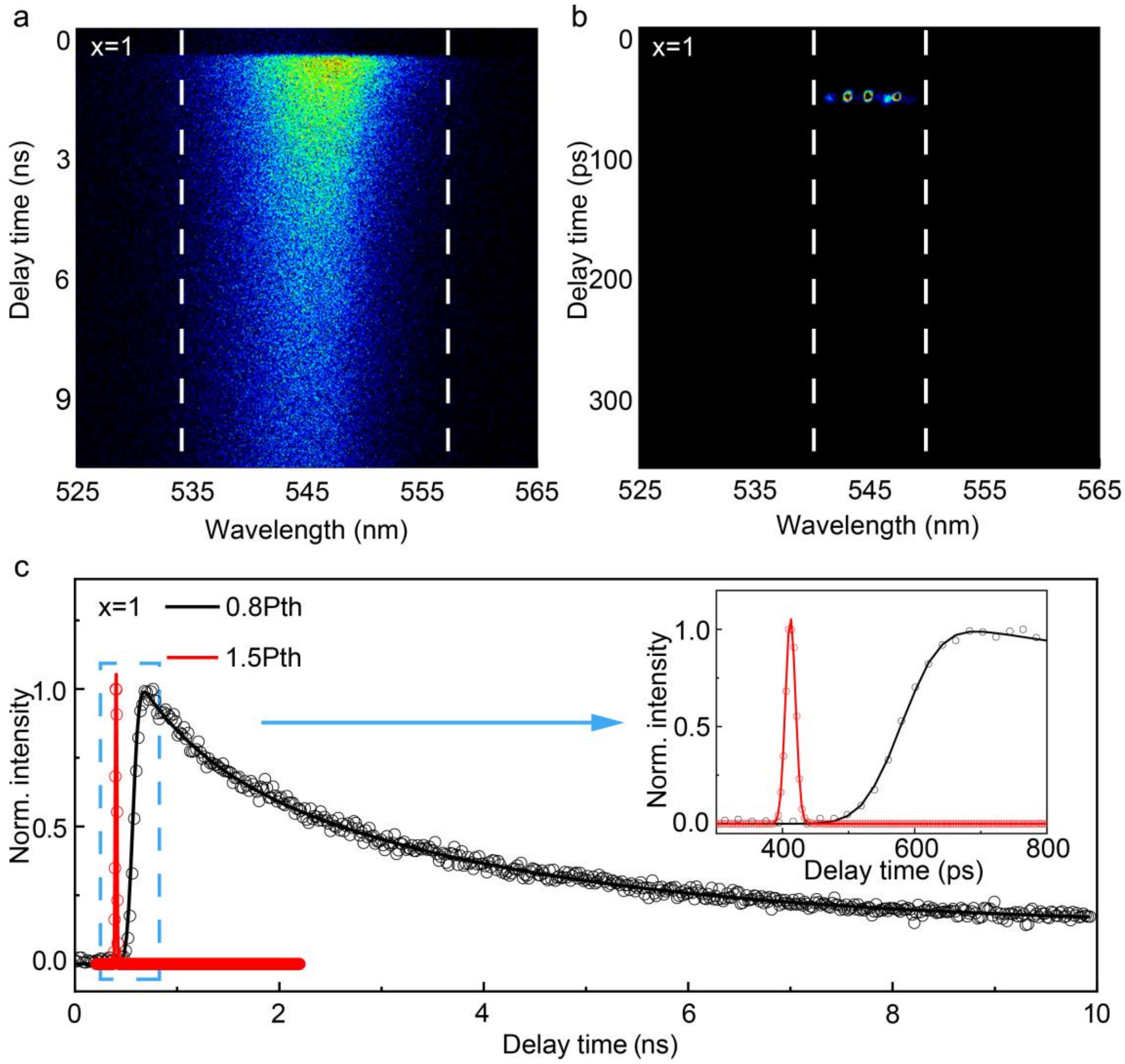

Figure S14. Streak-camera images of a $CsPbBr_3$ nanosheet recorded under excitation power of (a) 0.8 P_{th} (threshold excitation power) and (b) 1.5 P_{th}. (c) Typical PL-decay curve obtained at two different excitation powers (0.8, 1.5 P_{th}).

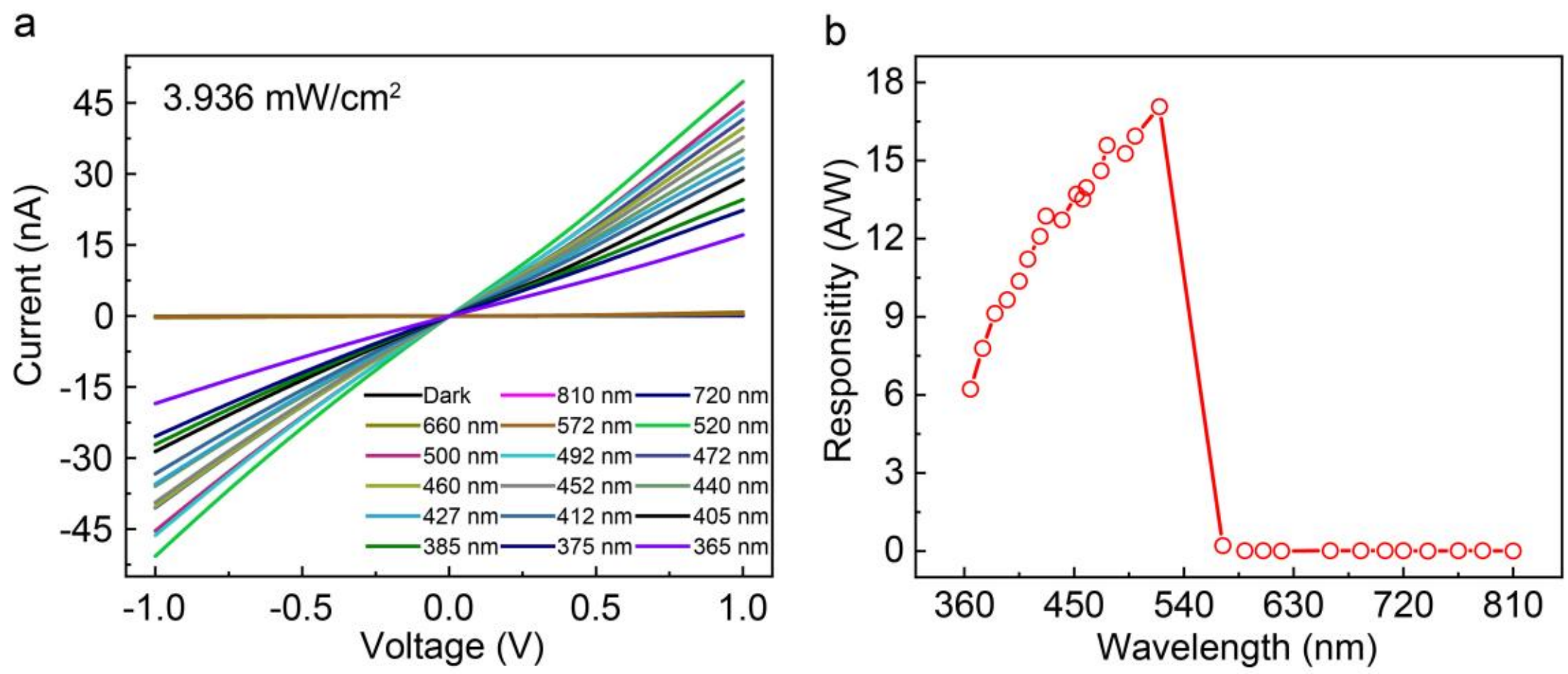

Figure S15. (a) Wavelength-dependent I-V curves of the $CsPbBr_3$ PD measured in the dark and at consistent illumination power densities. (b) Wavelength-dependent R at 1 V bias.

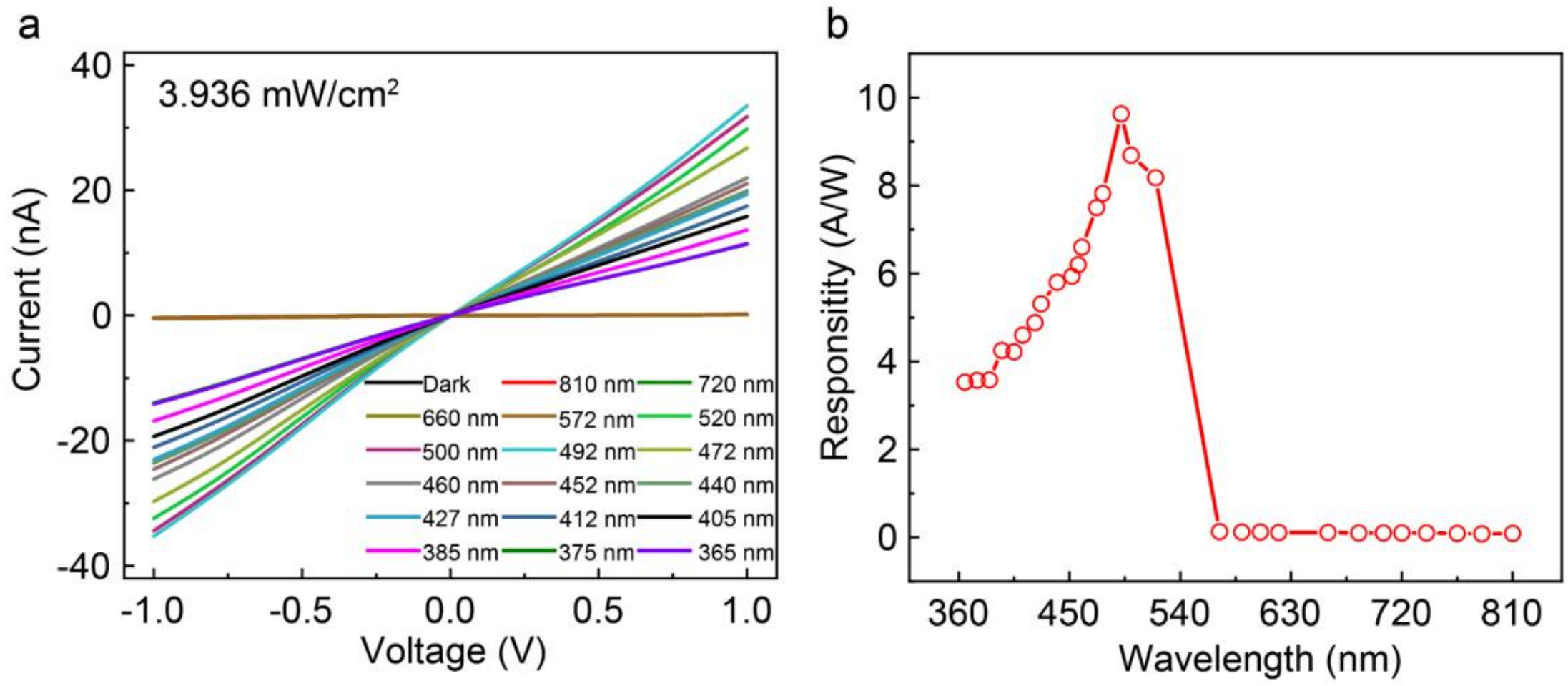

Figure S16. (a) Wavelength-dependent I-V curves of the $Cs_{0.81}Rb_{0.19}PbBr_3$ PD measured in the dark and at consistent illumination power densities. (b) Wavelength-dependent R at 1 V bias.

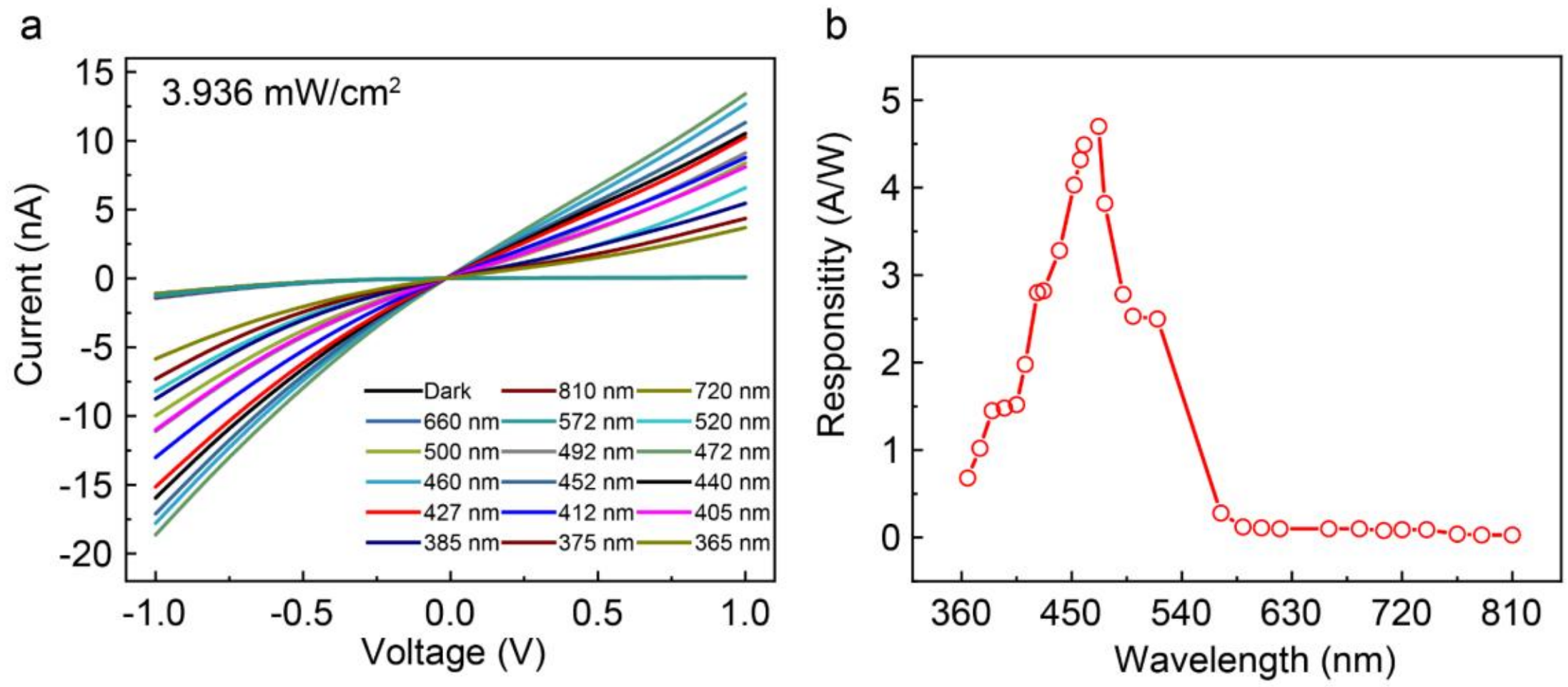

Figure S17. (a) Wavelength-dependent I-V curves of the $Cs_{0.62}Rb_{0.38}PbBr_3$ PD measured in the dark and at consistent illumination power densities. (b) Wavelength-dependent R at 1 V bias.

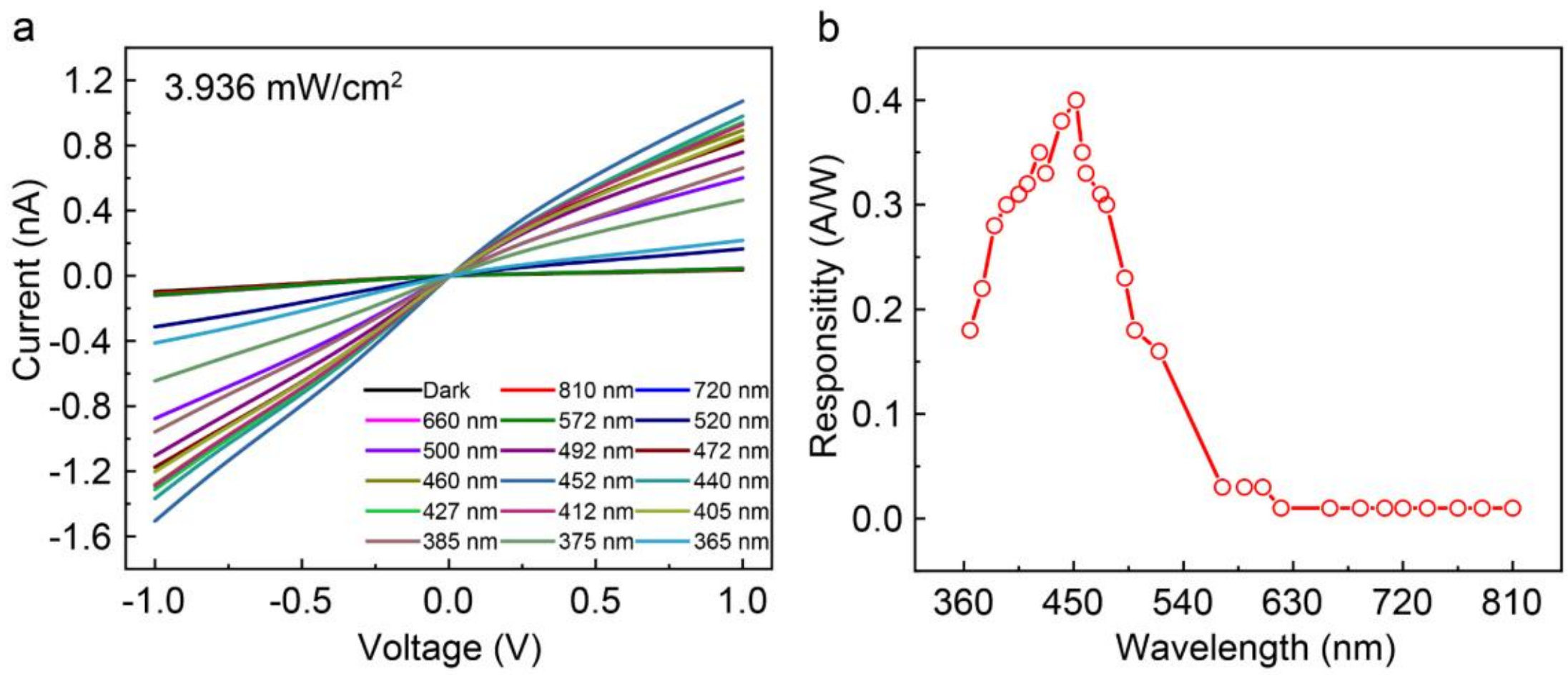

Figure S18. (a) Wavelength-dependent I-V curves of the $Cs_{0.24}Rb_{0.76}PbBr_3$ PD measured in the dark and at consistent illumination power densities. (b) Wavelength-dependent R at 1 V bias.

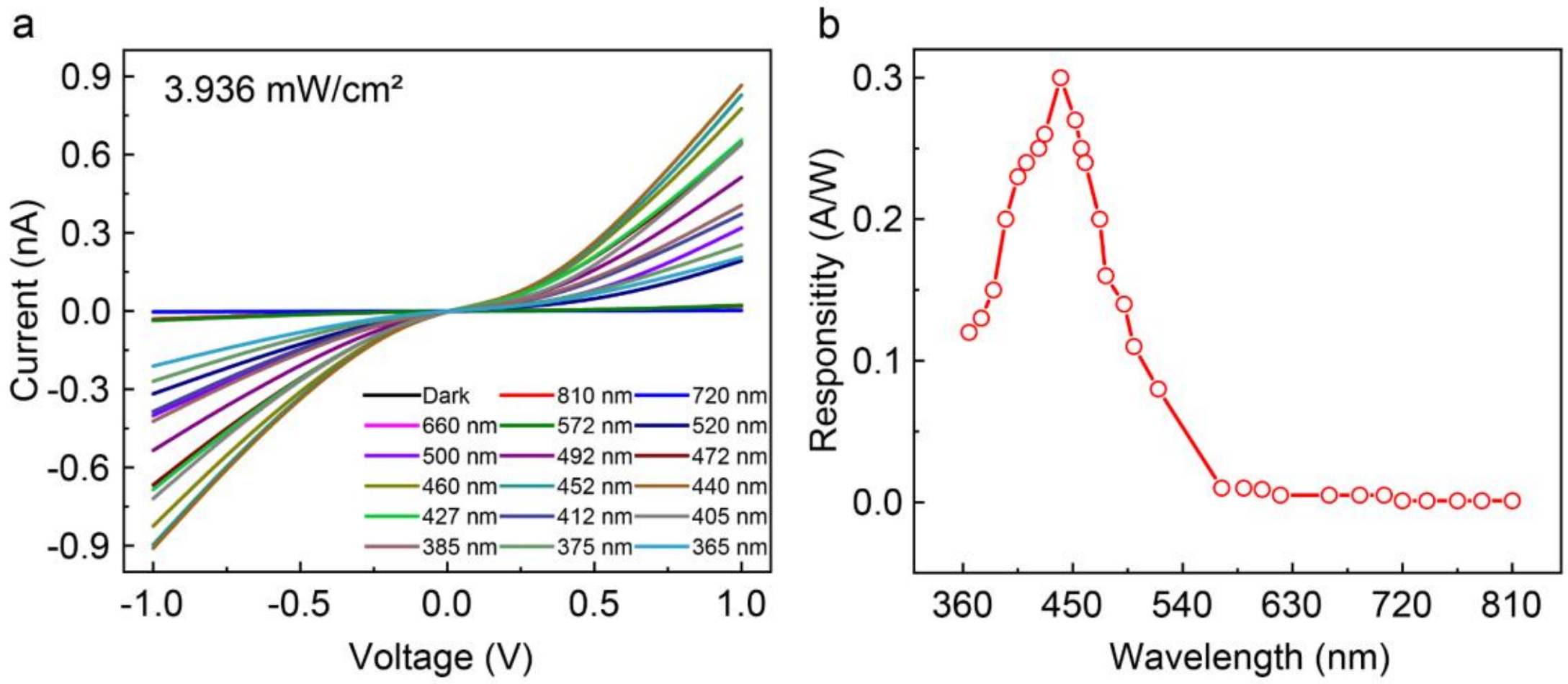

Figure S19. (a) Wavelength-dependent I-V curves of the $RbPbBr_3$ PD measured in the dark and at consistent illumination power densities. (b) Wavelength-dependent R at 1 V bias.

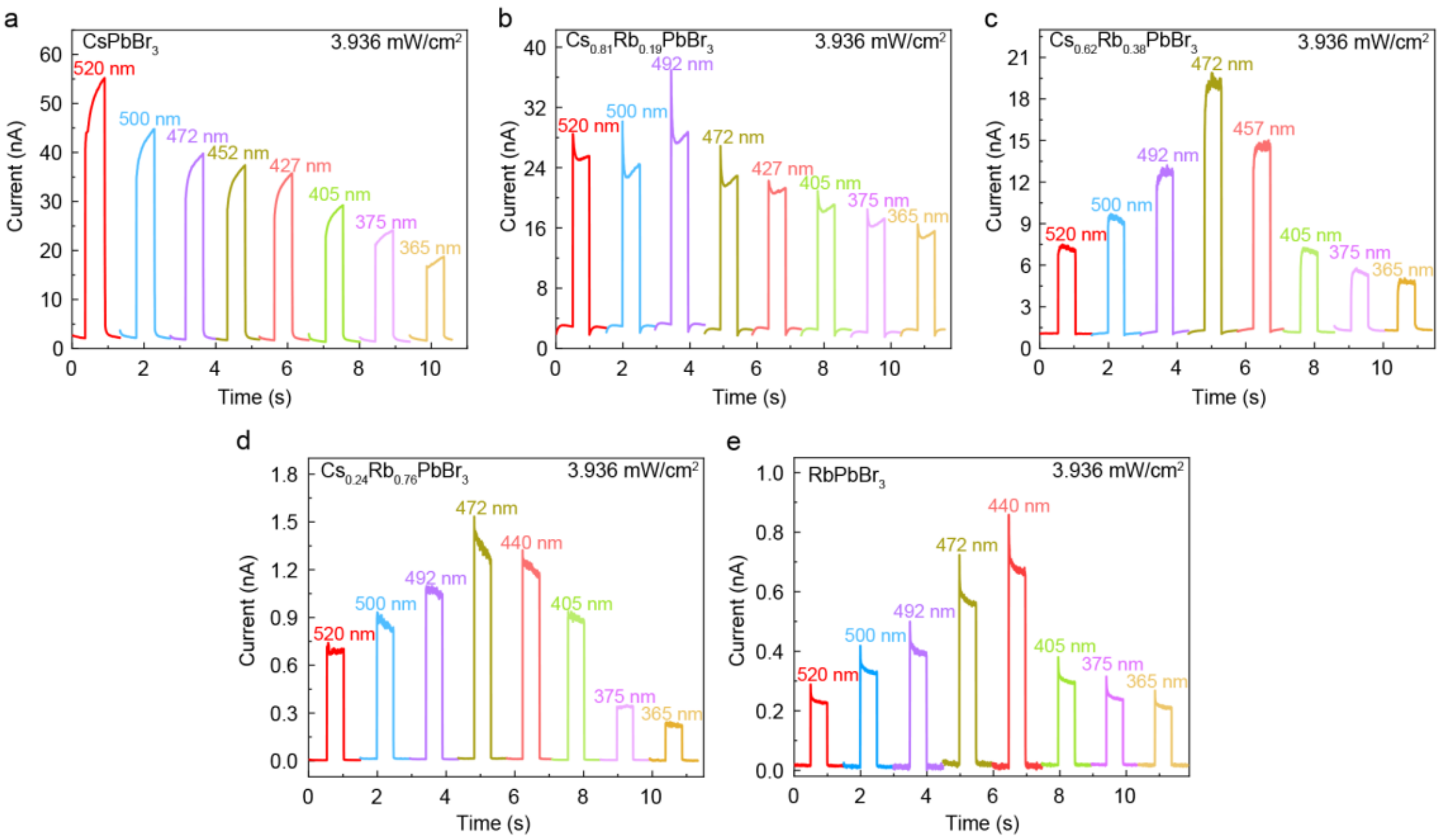

Figure S20. I-T curves of five component PDs under periodic illumination at different wavelengths.

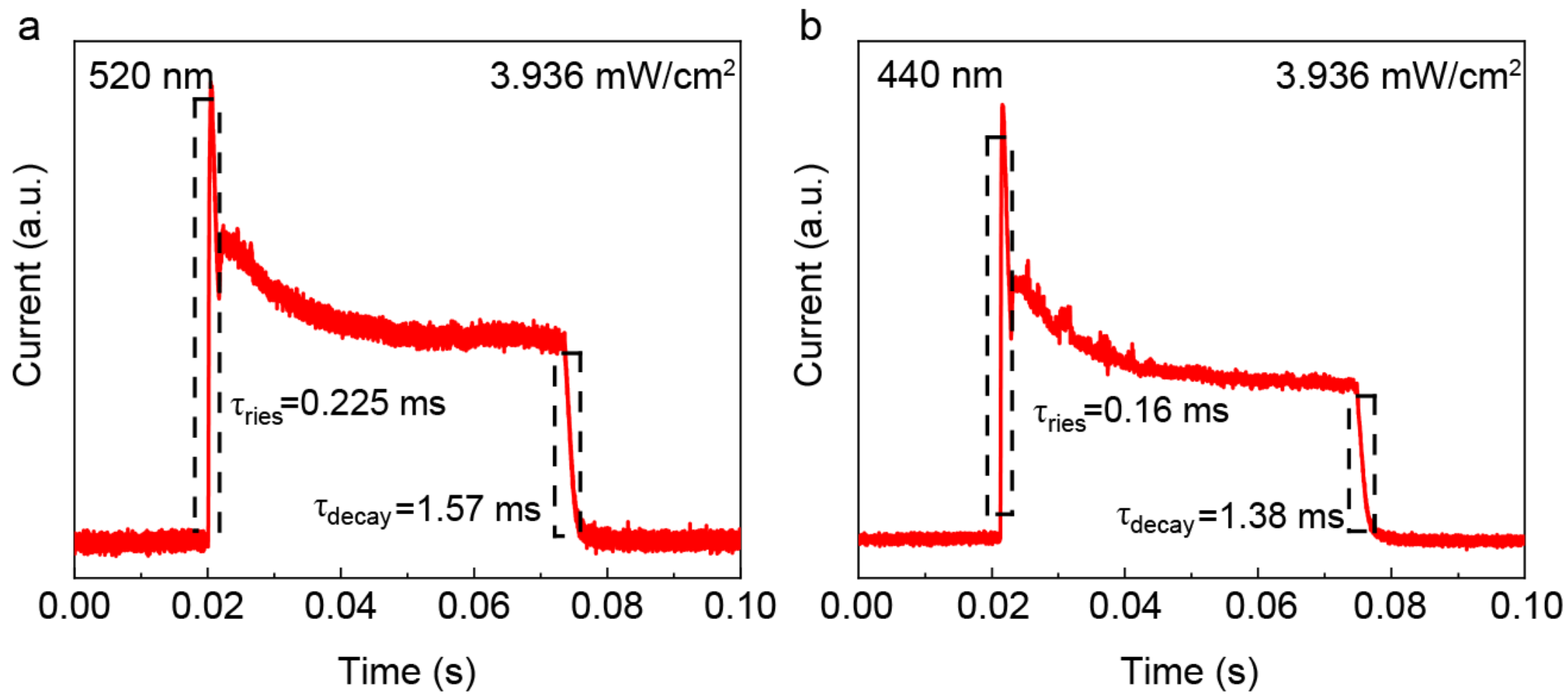

Figure S21. (a) Rise and fall time of the $CsPbBr_3$ PD measured under 1 V. (b) Rise and fall time of the $RbPbBr_3$ PD measured under 1 V.

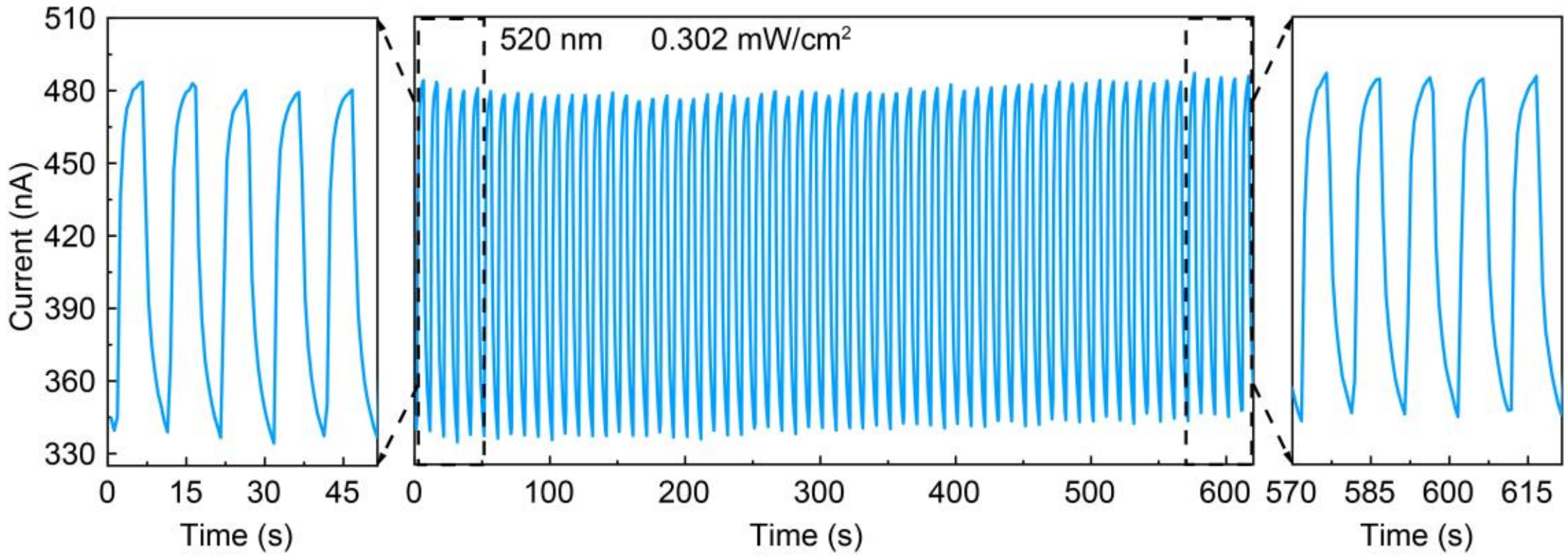

Figure S22. I-T curve of the $CsPbBr_3$ PD for 620 s.

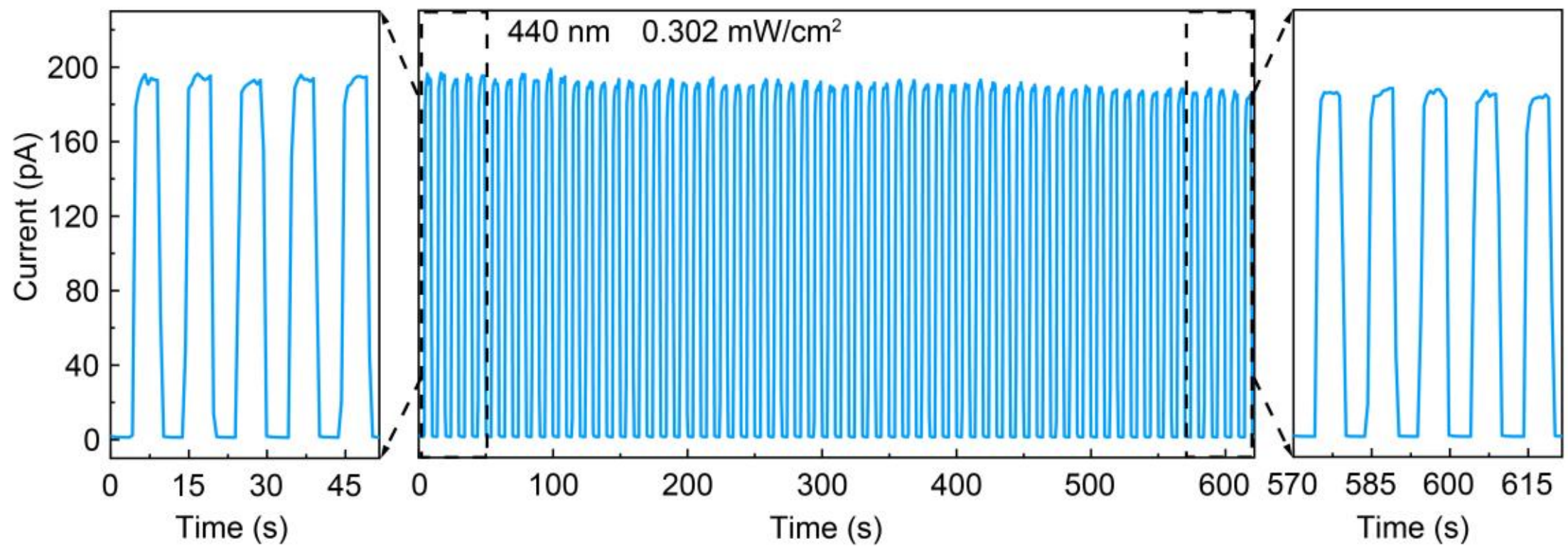

Figure S23. I-T curve of the $RbPbBr_3$ PD for 620 s.

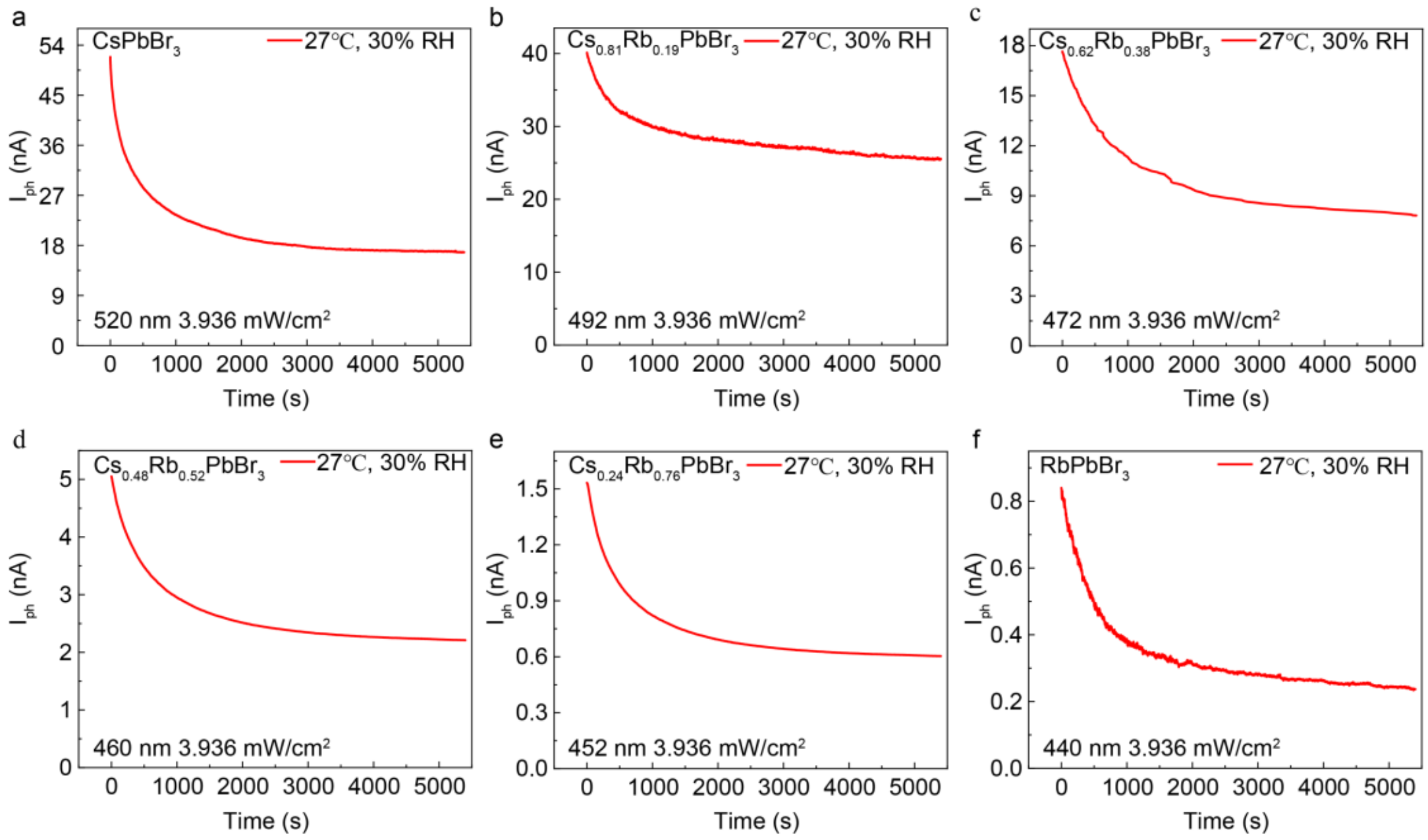

Figure S24. Time stability of devices at same humidity levels of $CsPbBr_3$, $Cs_{0.81}Rb_{0.19}PbBr_3$, $Cs_{0.62}Rb_{0.38}PbBr_3$, $Cs_{0.48}Rb_{0.52}PbBr_3$, $Cs_{0.24}Rb_{0.76}PbBr_3$, and $RbPbBr_3$ PDs.

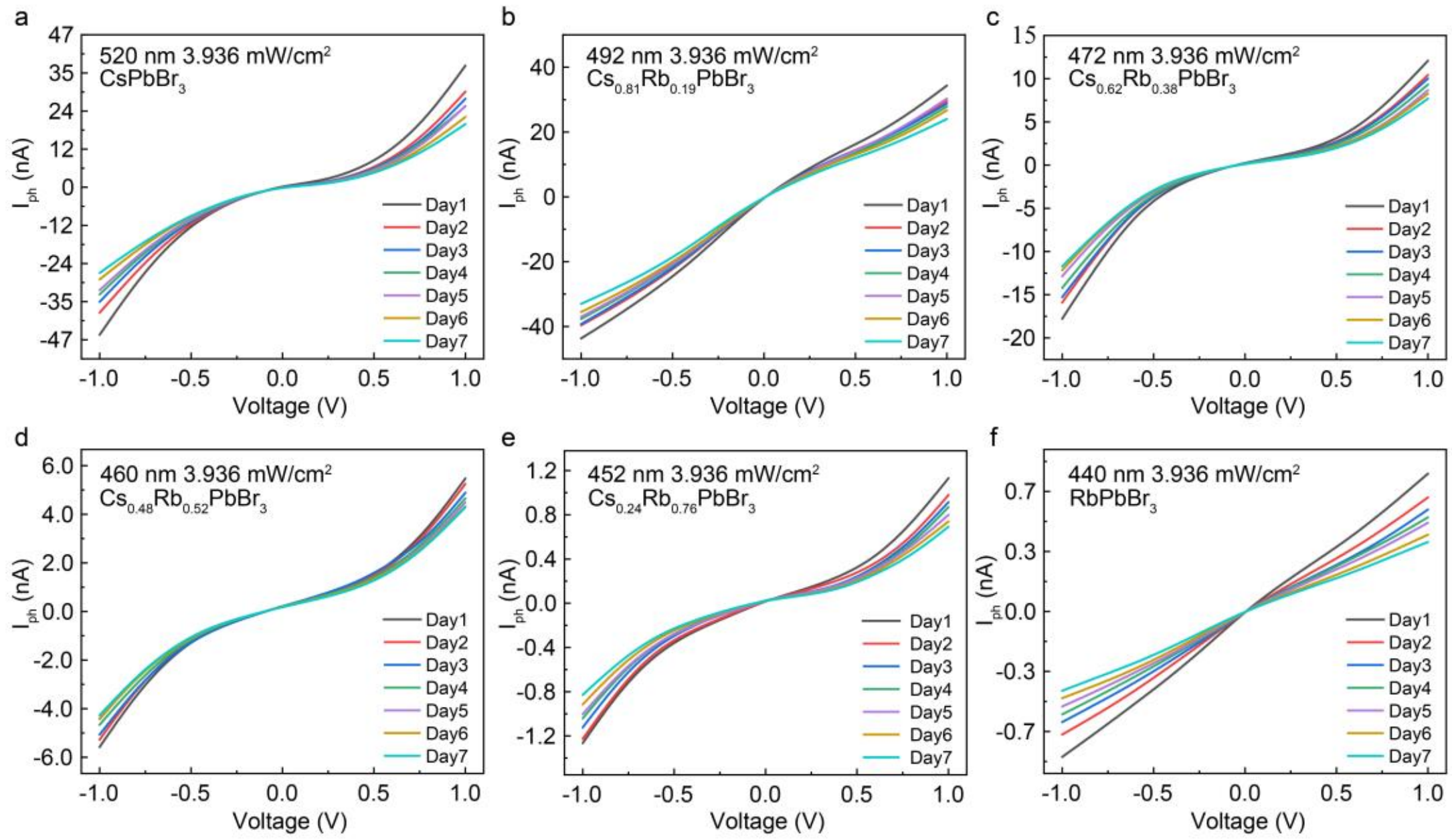

Figure S25. I-V curves as a function of light irradiance for alloy devices with different compositions, measured after various days of air exposure.

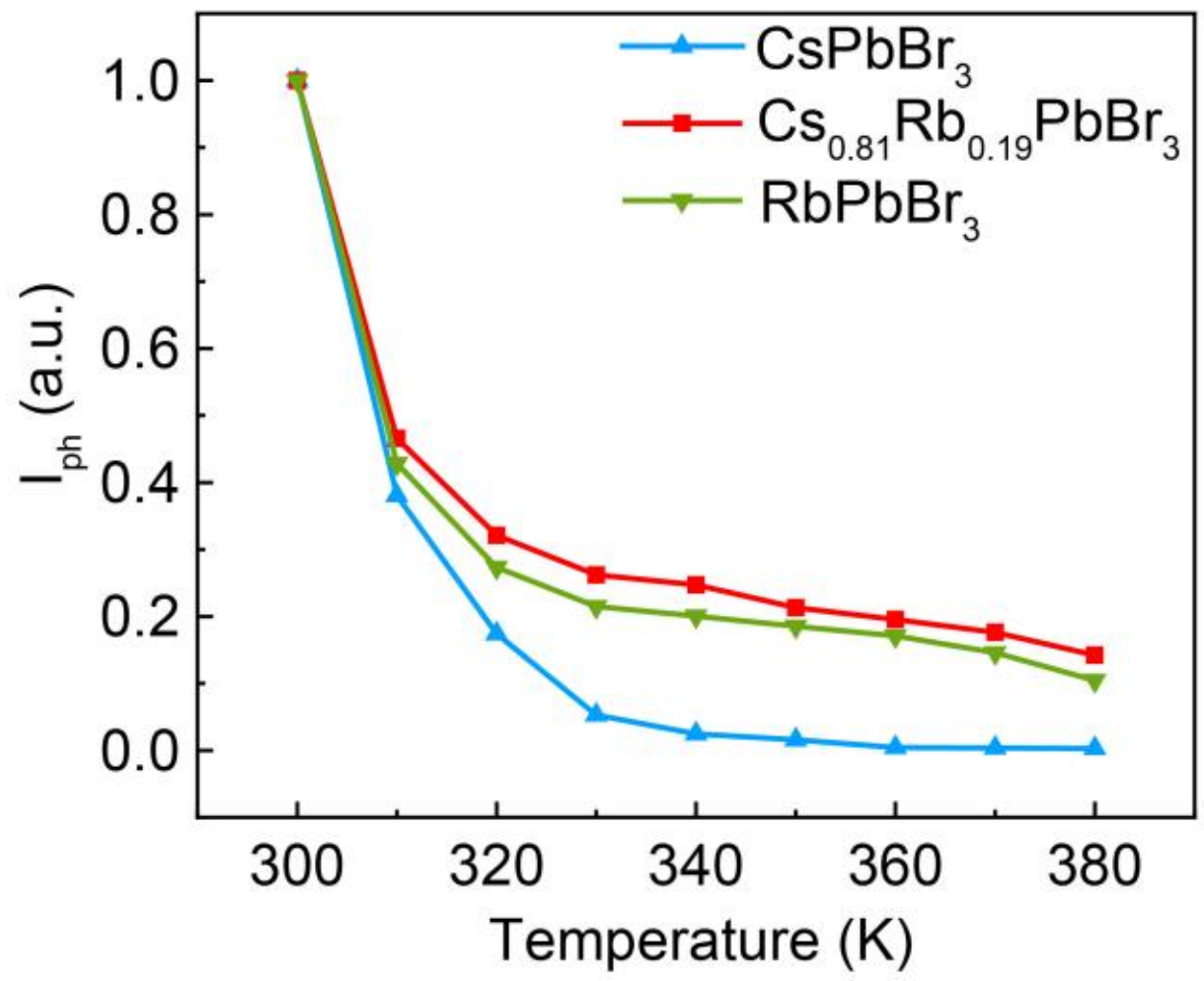

Figure S26. The effect of temperature on the performance of $CsPbBr_3$, $Cs_{0.81}Rb_{0.19}PbBr_3$ and $RbPbBr_3$ PDs.

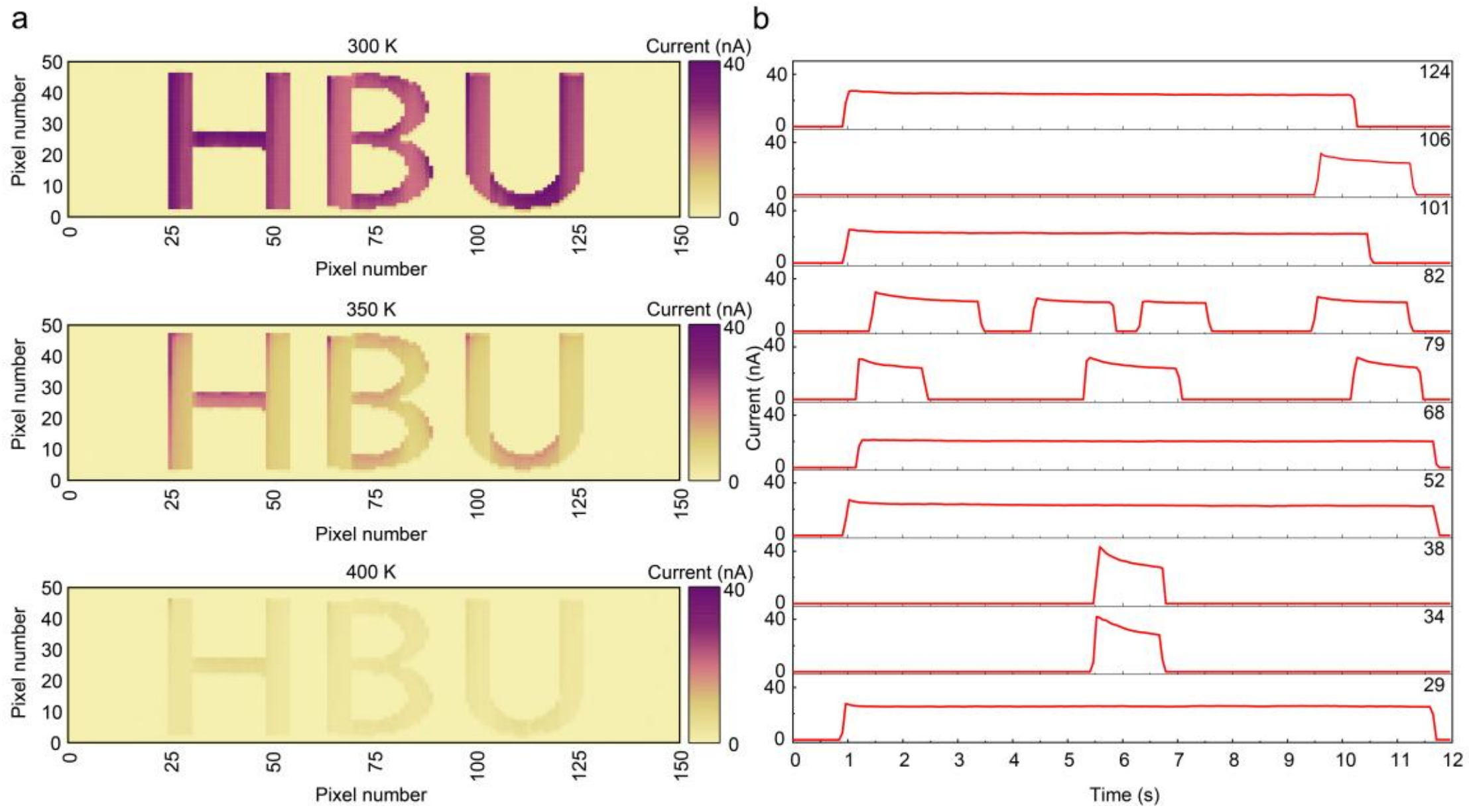

Figure S27. (a) Current mappings of the "HBU" signal pattern under 300, 350 and 400 K light irradiation of the $Cs_{0.81}Rb_{0.19}PbBr_3$ PDs. (b) Time-dependent current curves of "HBU" under a 492 nm laser scans along the Y direction with different X pixel points (numbers on the right side).

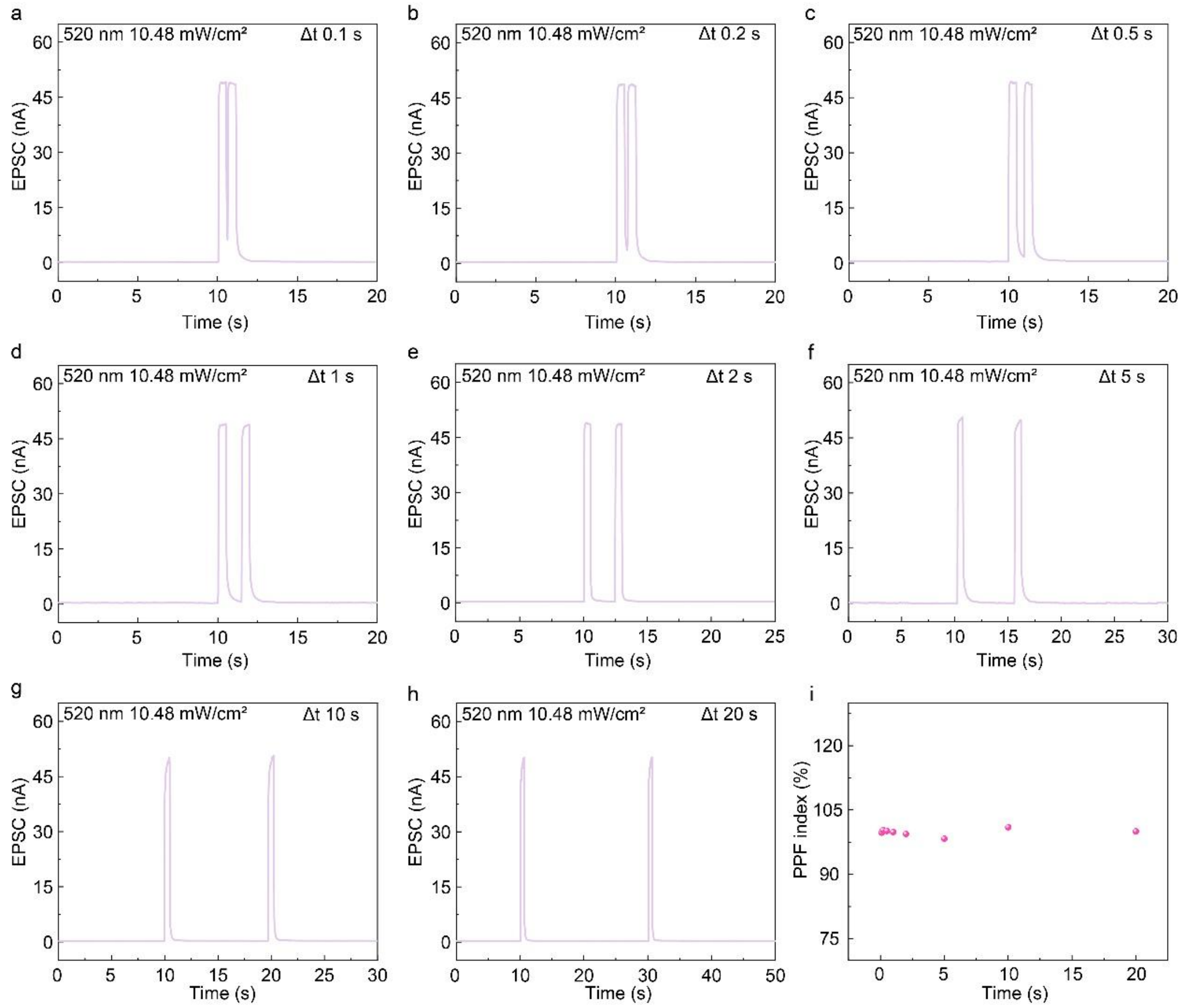

Figure S28. Optical response and PPF fitting curve at 520 nm under different pulse (Without HfO_2).

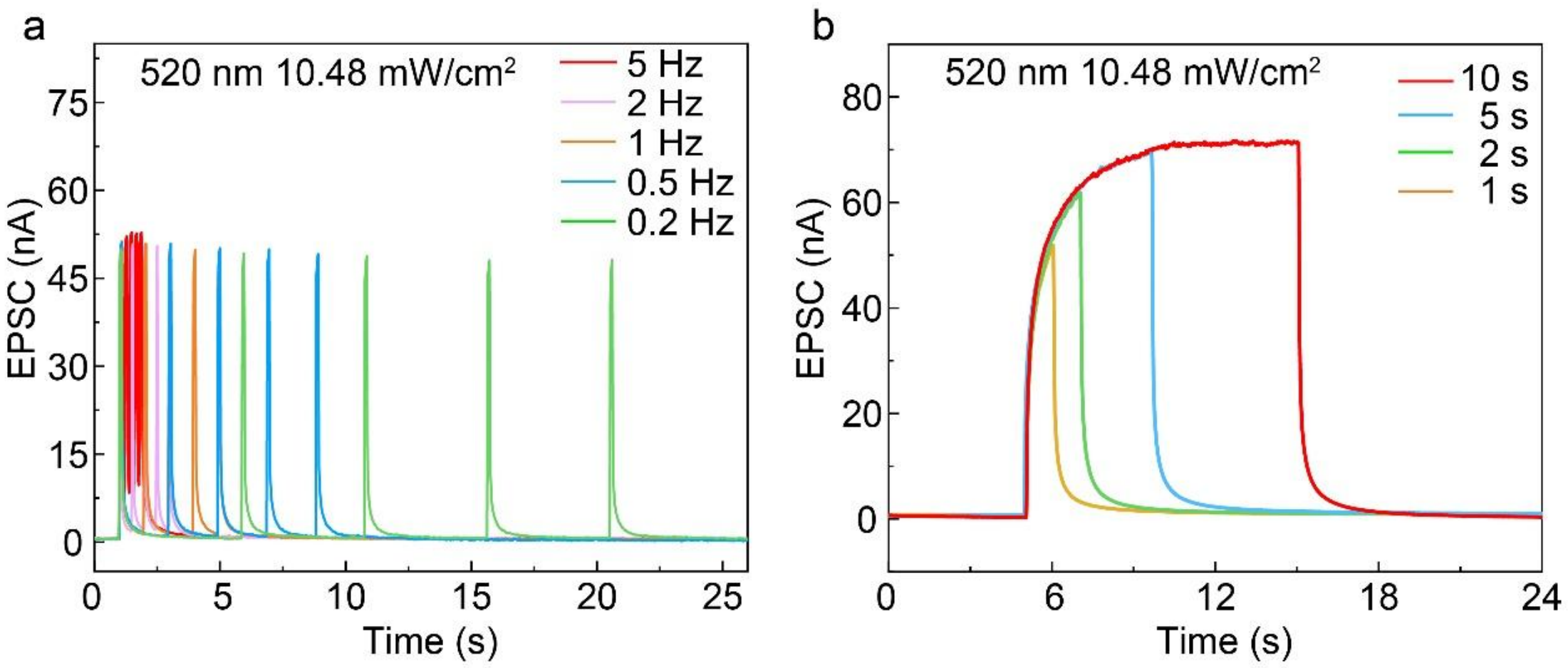

Figure S29. Synaptic responses under 520 nm illumination with varying (c) pulse width and (d) pulse frequency (Without HfO_2).

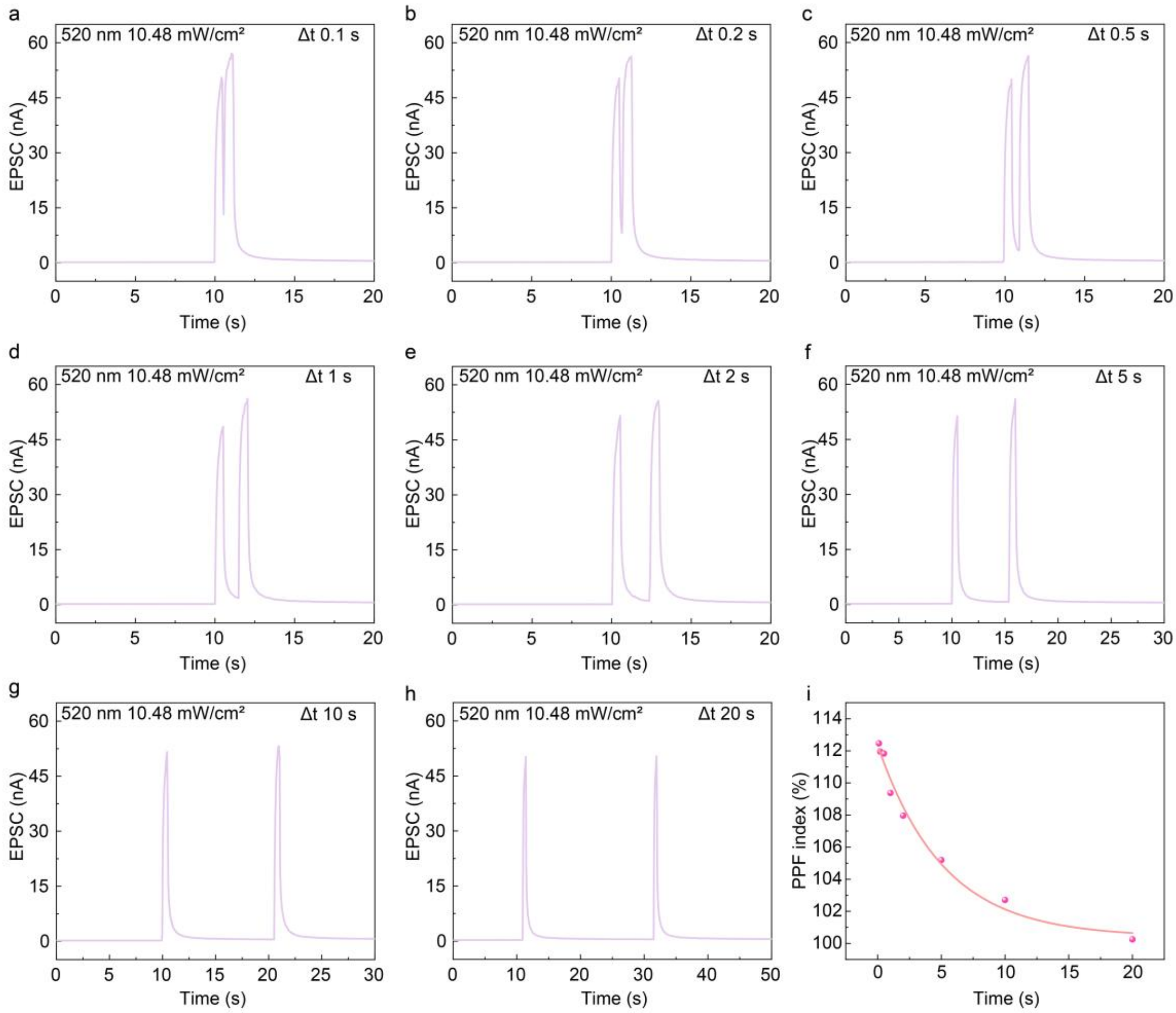

Figure S30. Optical response and PPF fitting curve at 520 nm under different pulse durations.

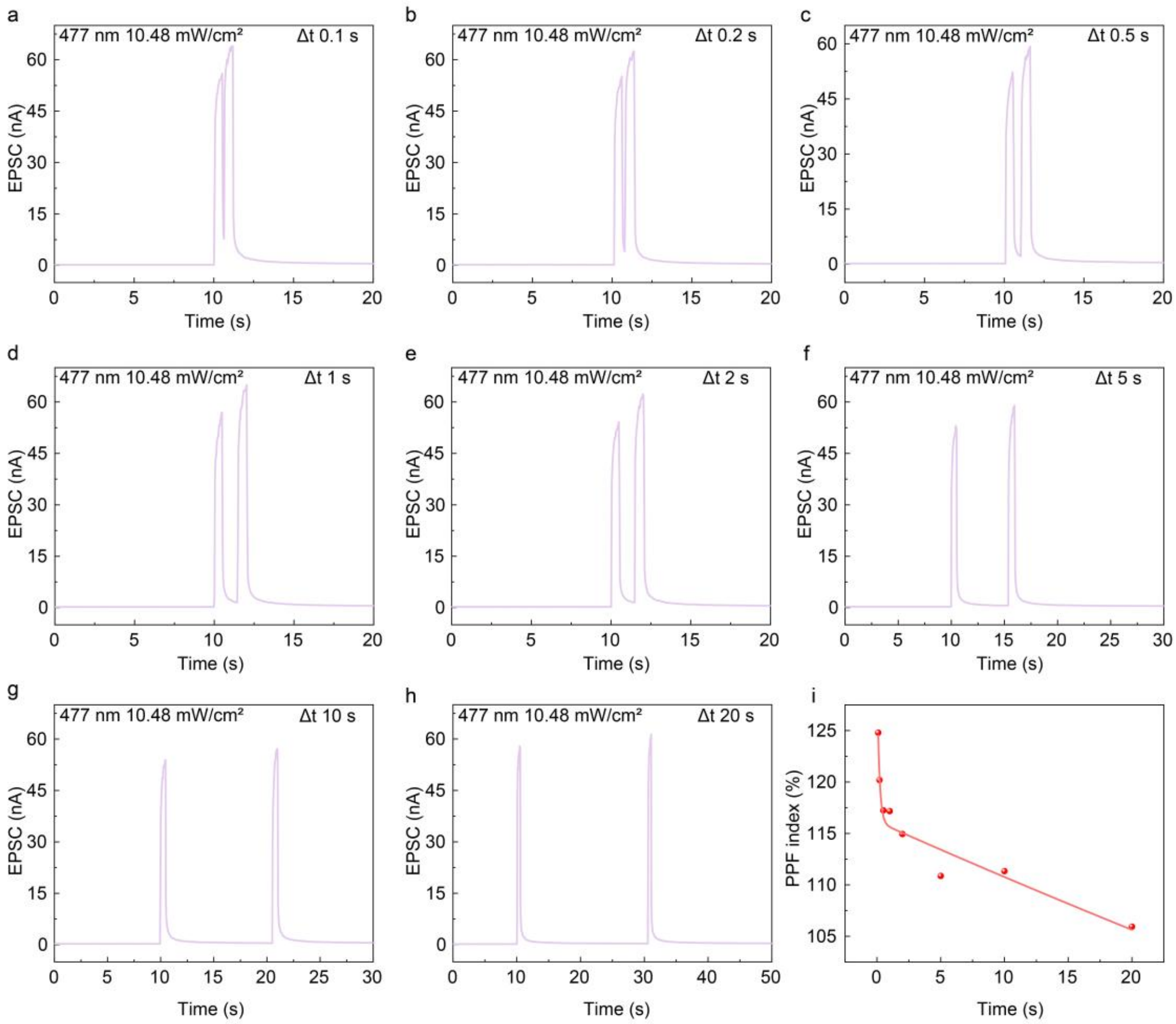

Figure S31. Optical response and PPF fitting curve at 477 nm under different pulse durations.

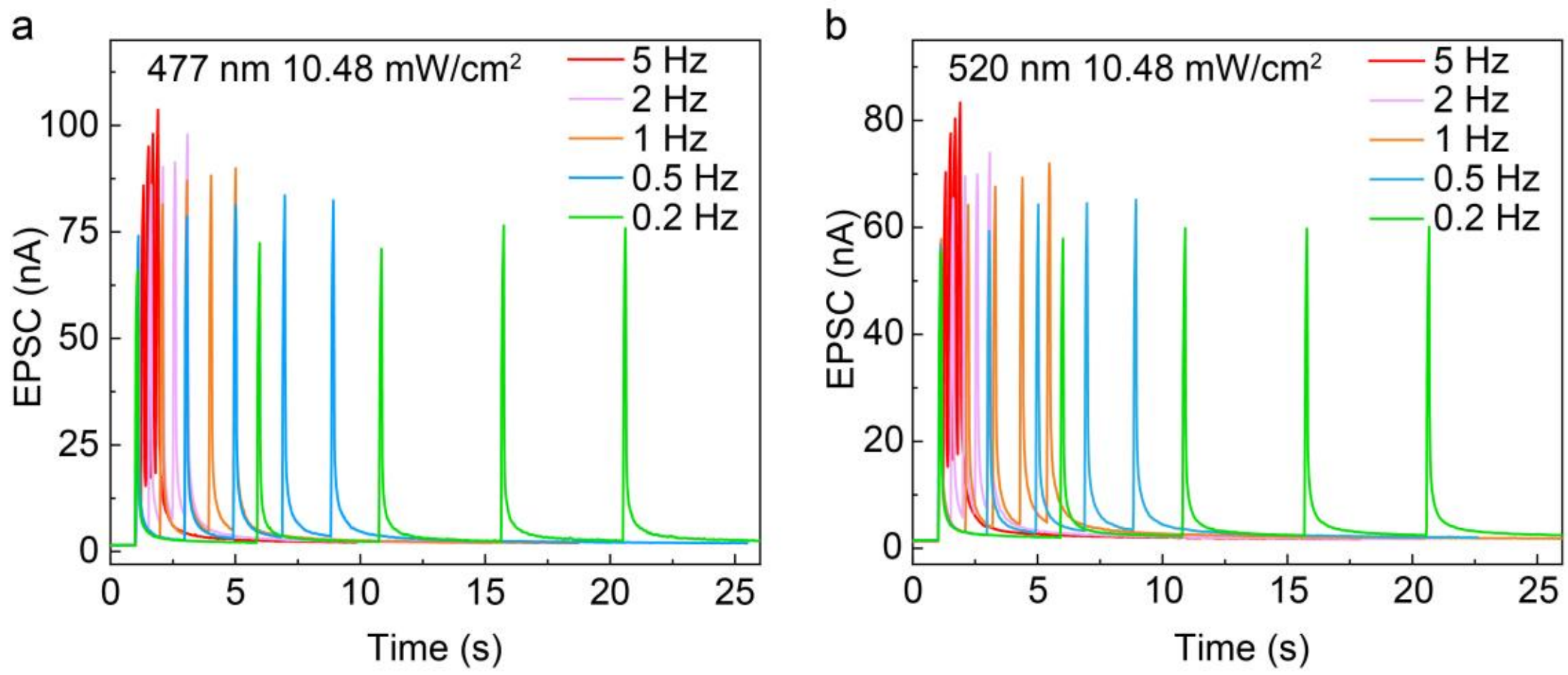

Figure S32. EPSC of the $Cs_{0.81}Rb_{0.19}PbBr_3$ synaptic device as a function of light-pulse frequency under (a) 477nm and (b) 520 nm illumination.

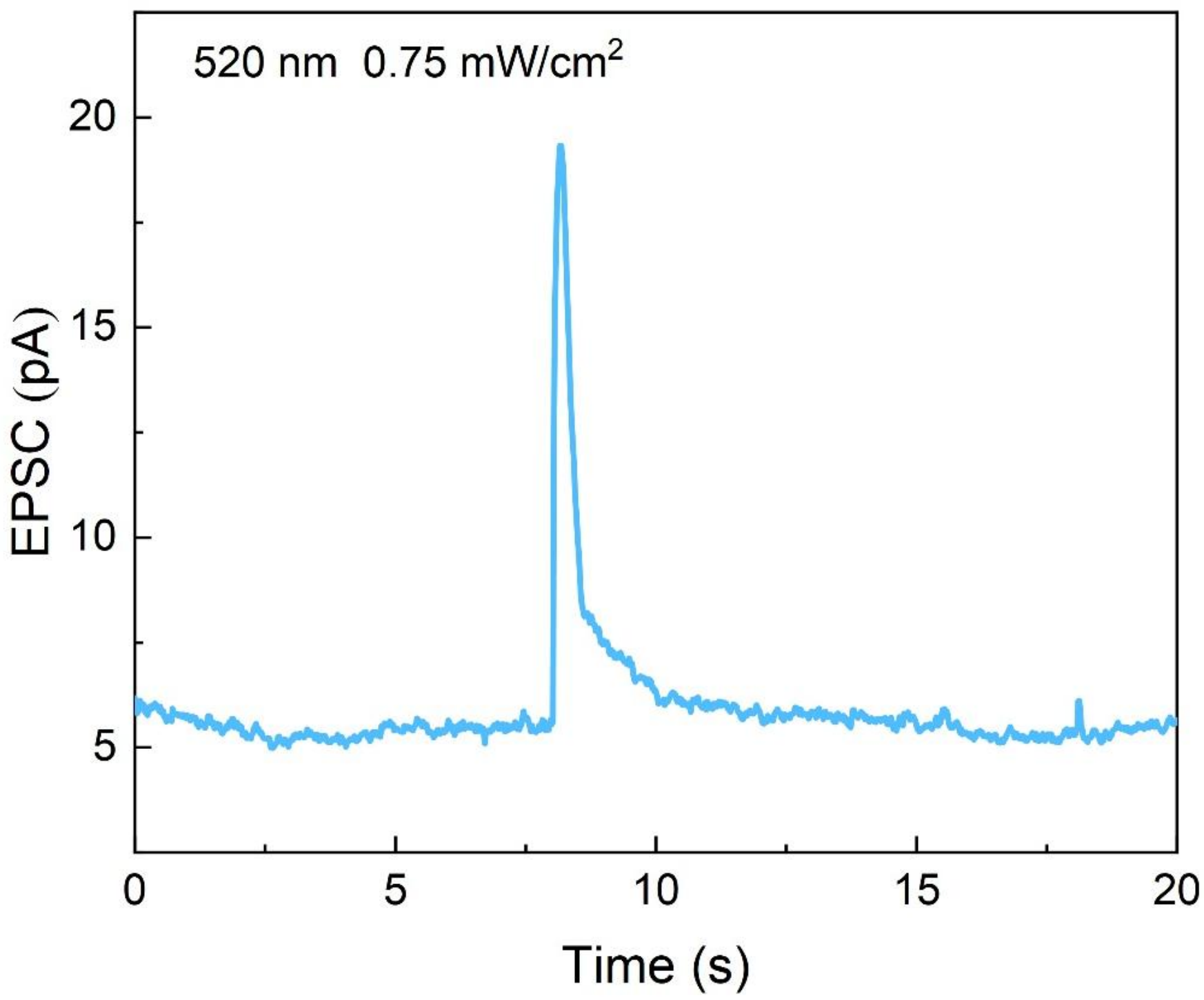

Figure S33. Energy consumption per synaptic event.

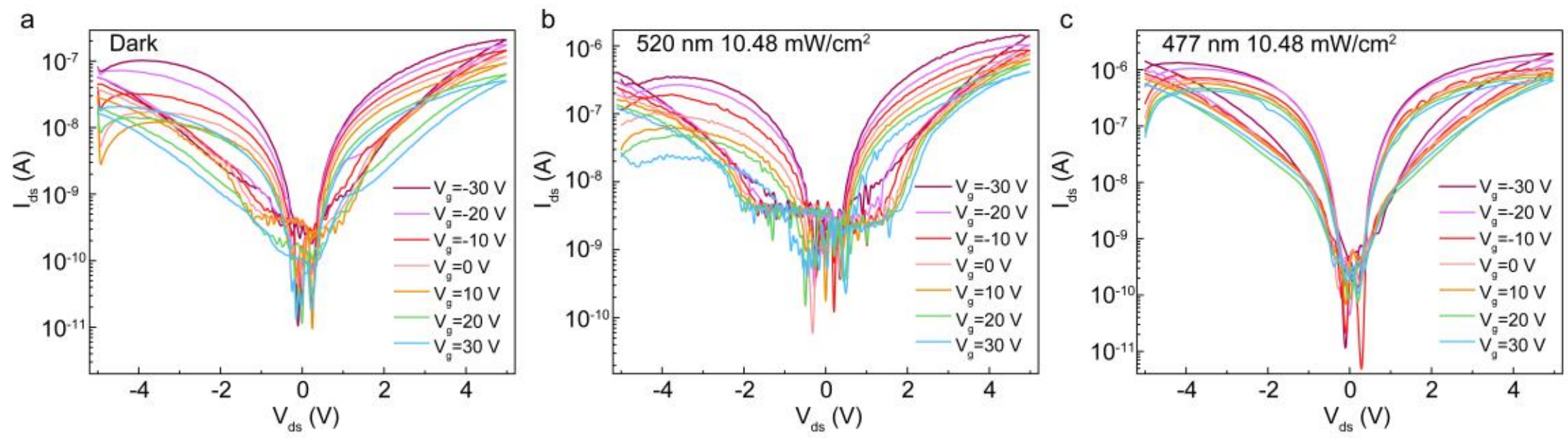

Figure S34. (a) Switching behavior curves of the device under dark conditions. (b) Switching behavior curves of the device under different back-gate conditions at 520 nm. (c) Switching behavior curves of the device under different back-gate conditions at 477 nm.

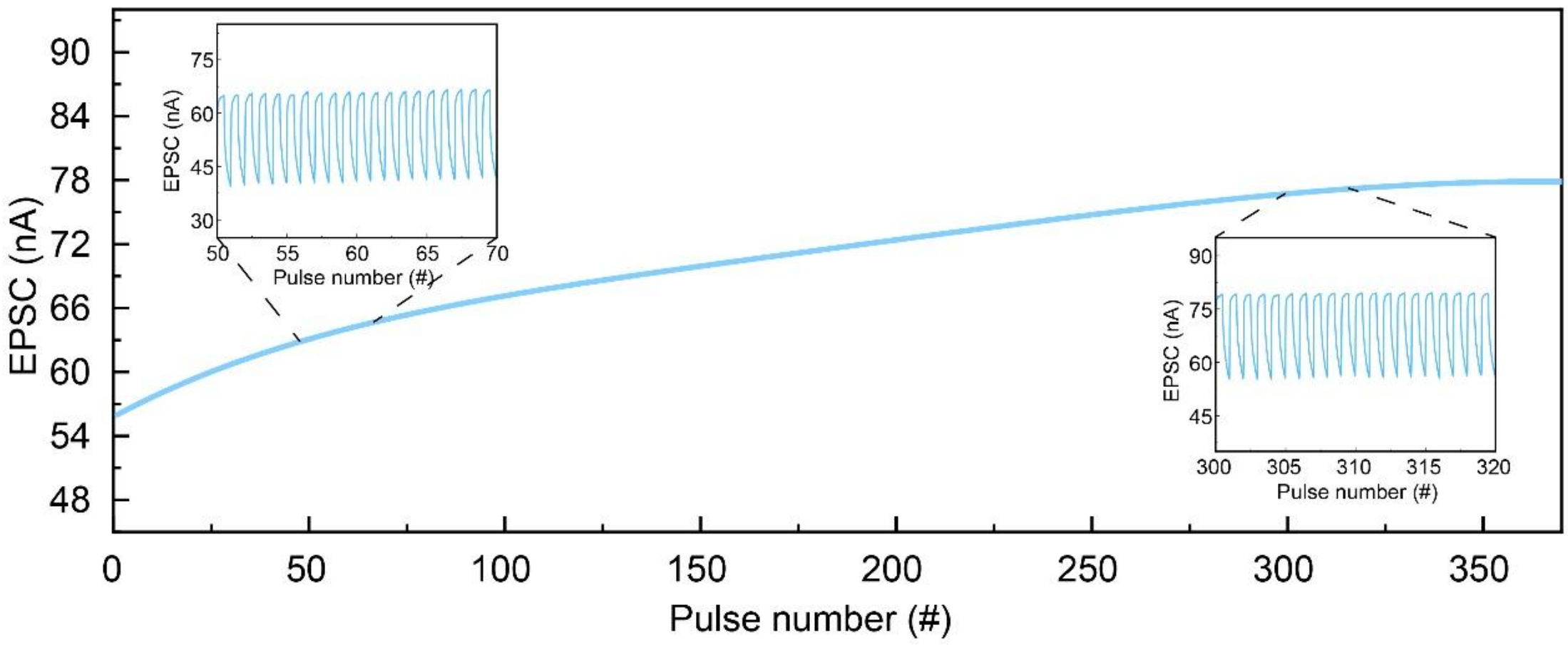

Figure S35. Conductance states under 375 times of input optical pulses. Insets show the randomly enlarged graphs of response curve.

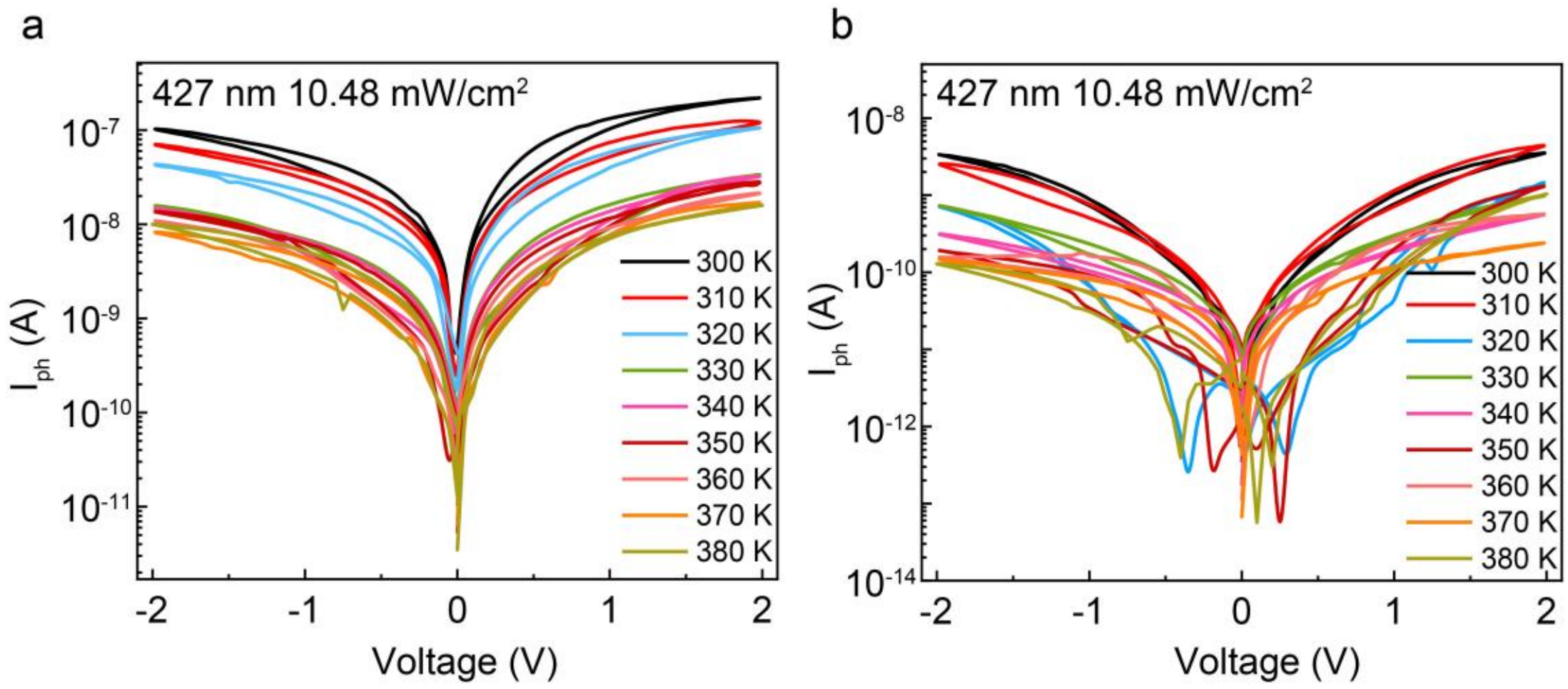

Figure S36. Switching behavior of $CsPbBr_3$ and $RbPbBr_3$ photosynaptic at elevated temperatures.

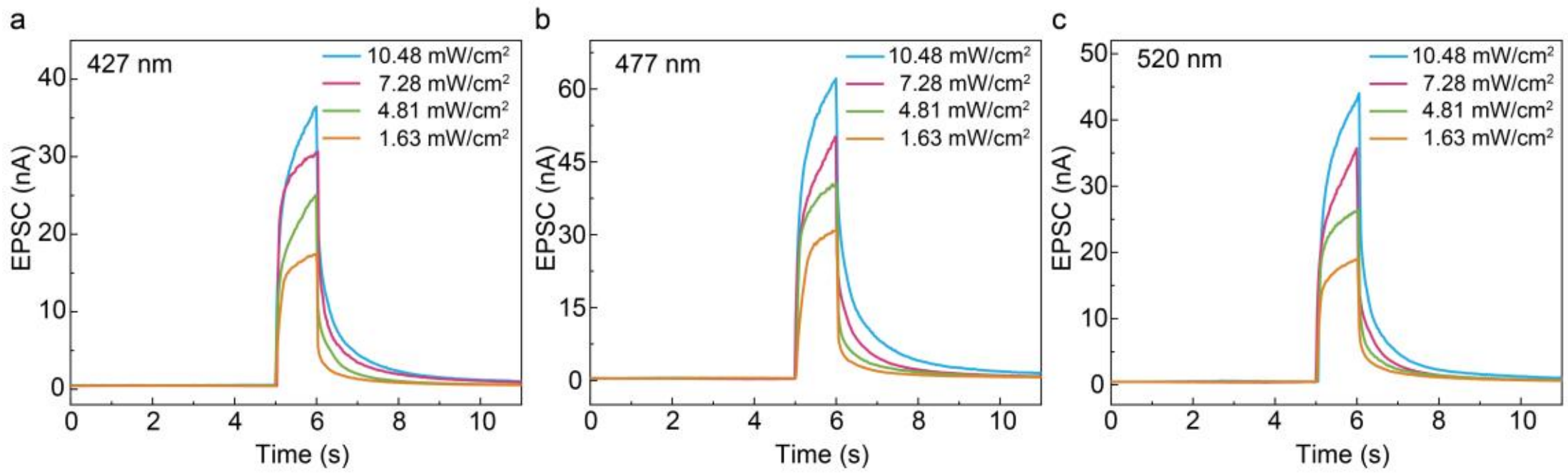

Figure S37. EPSC of the $Cs_{0.81}Rb_{0.19}PbBr_3$ synaptic device as a function of light-pulse power under (a) 427 nm and (b) 477 nm and (c)520 nm illumination.

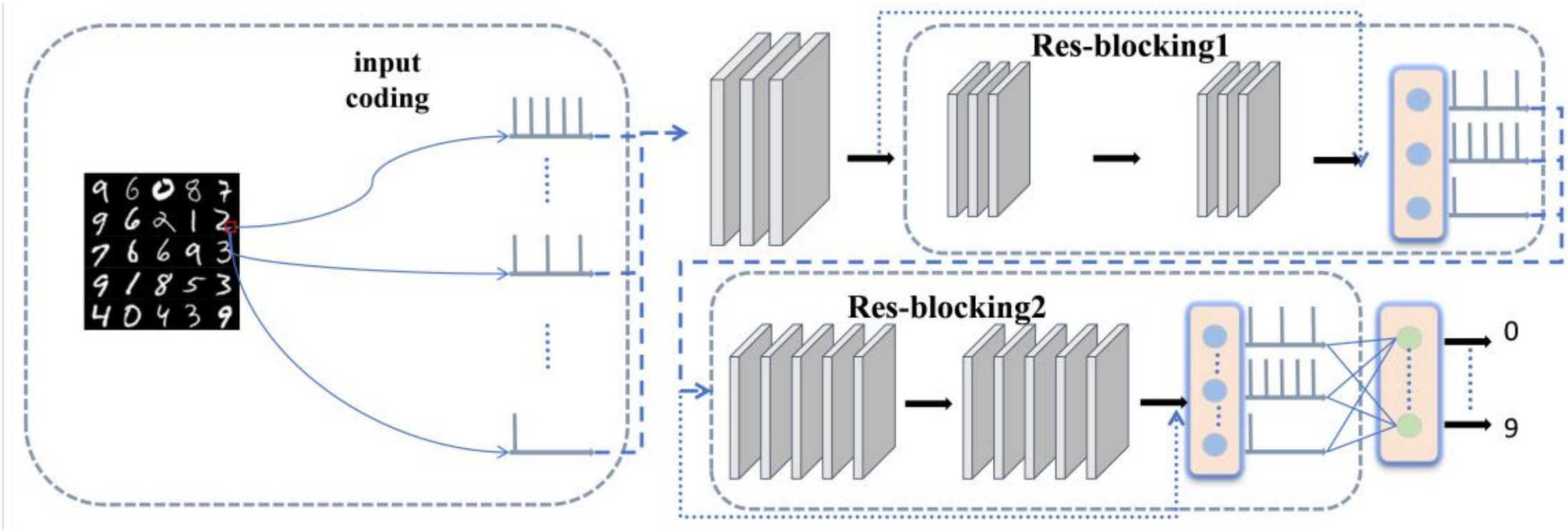

Figure S38. Network diagram for grayscale datasets such as the MNIST series.

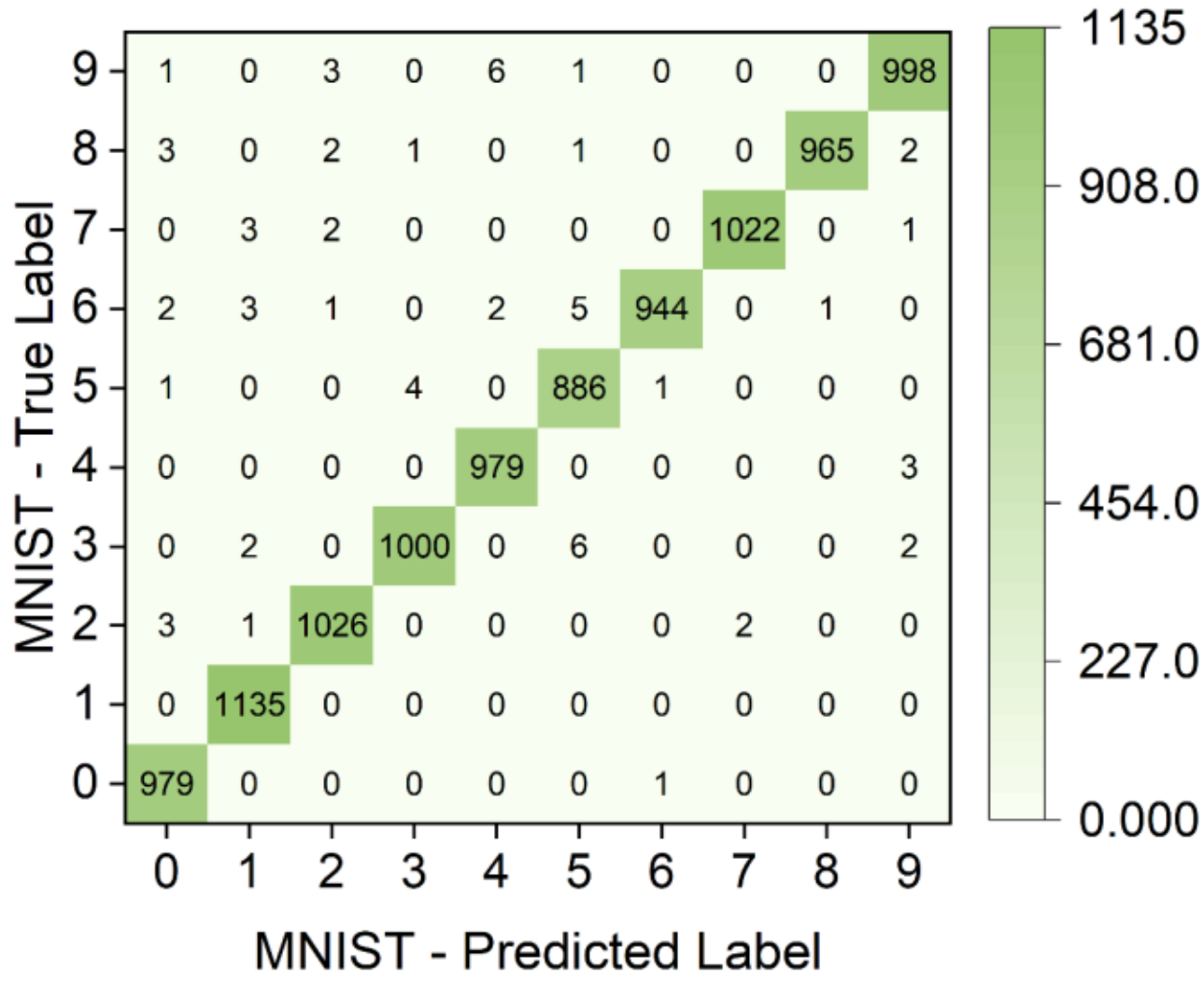

Figure S39. Confusion Matrix for the MNIST Dataset.

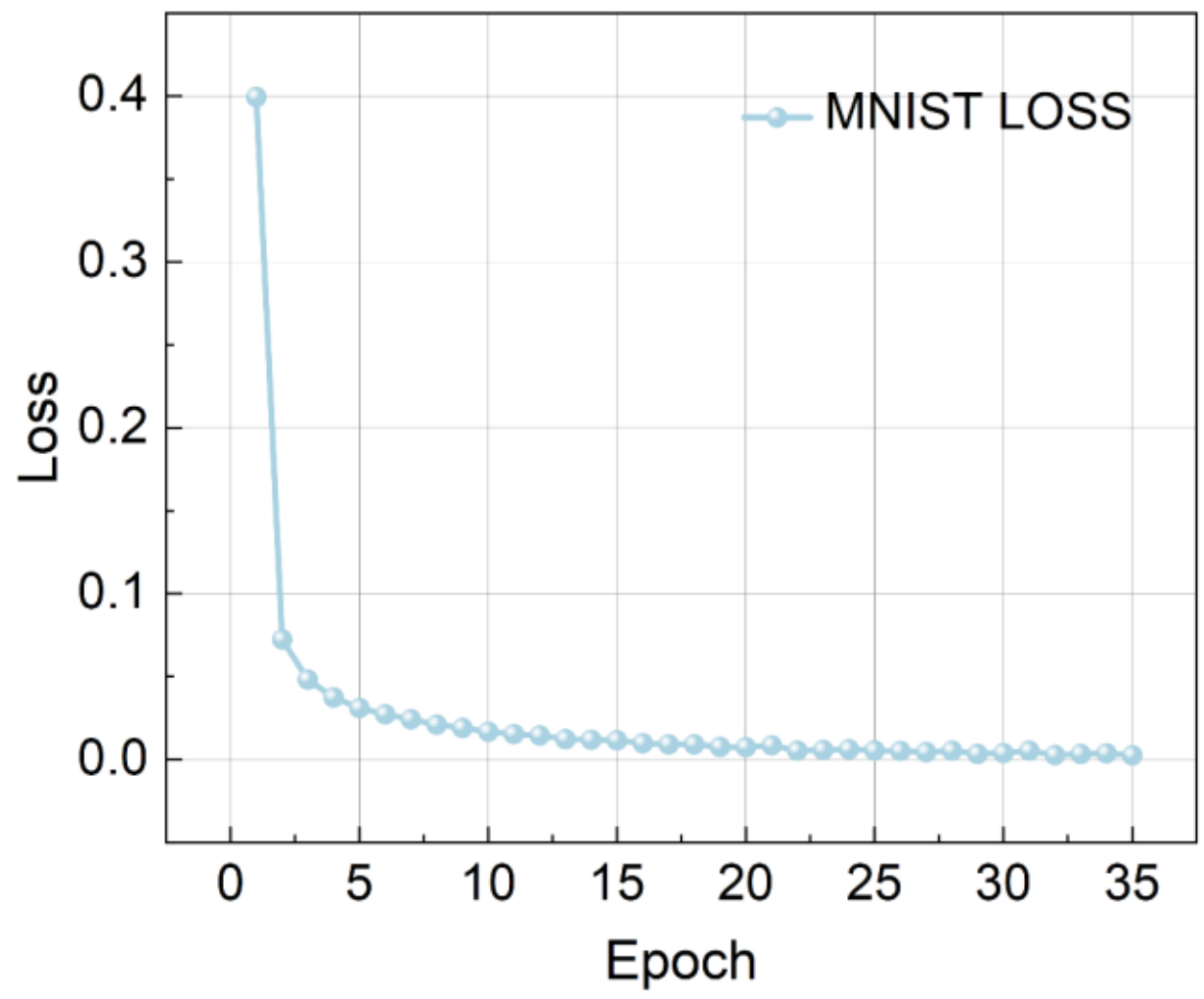

Figure S40. Loss Function for the MNIST Dataset.

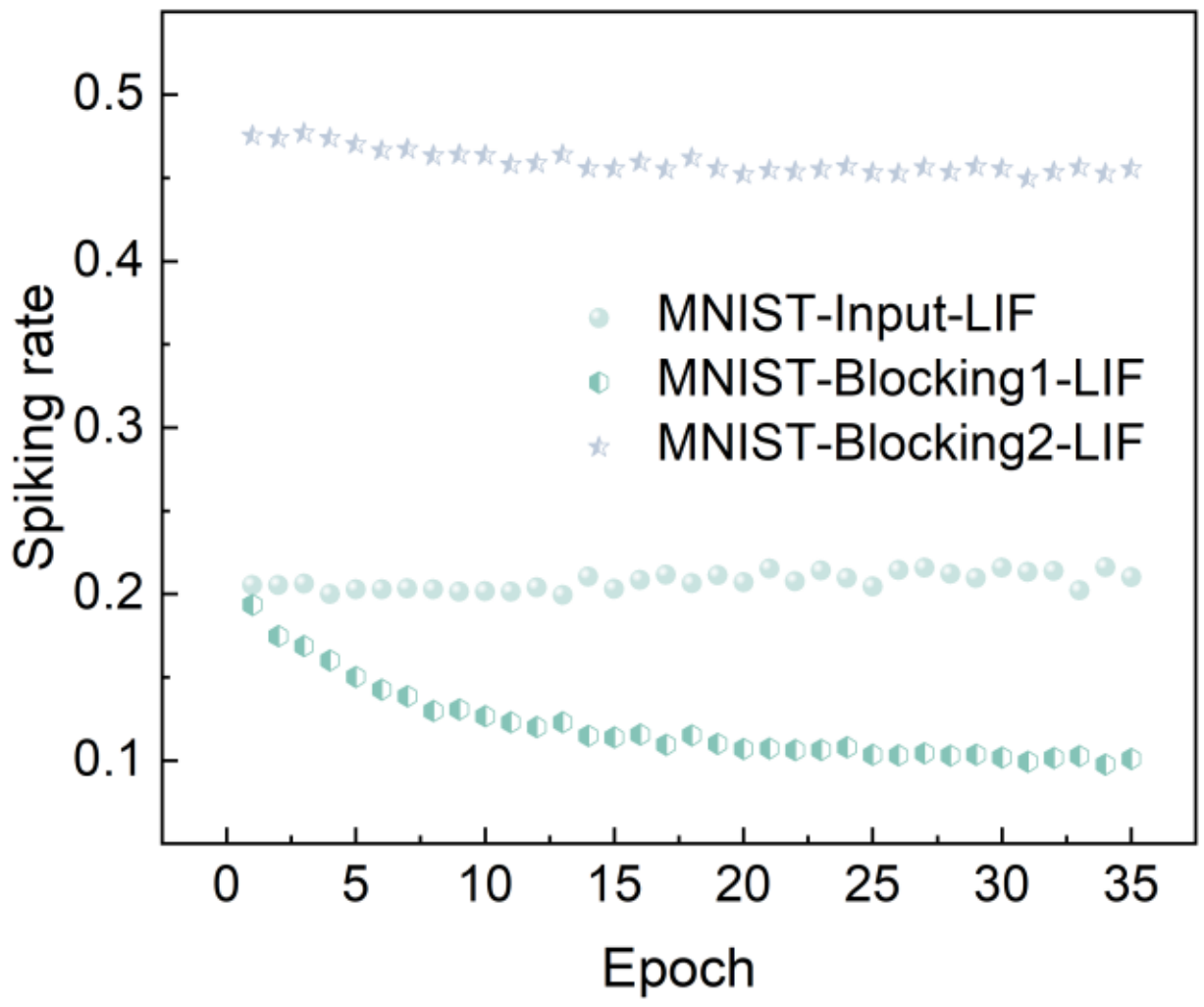

Figure S41. Variations in LIF Neuron Firing Frequency in the MNIST Dataset.

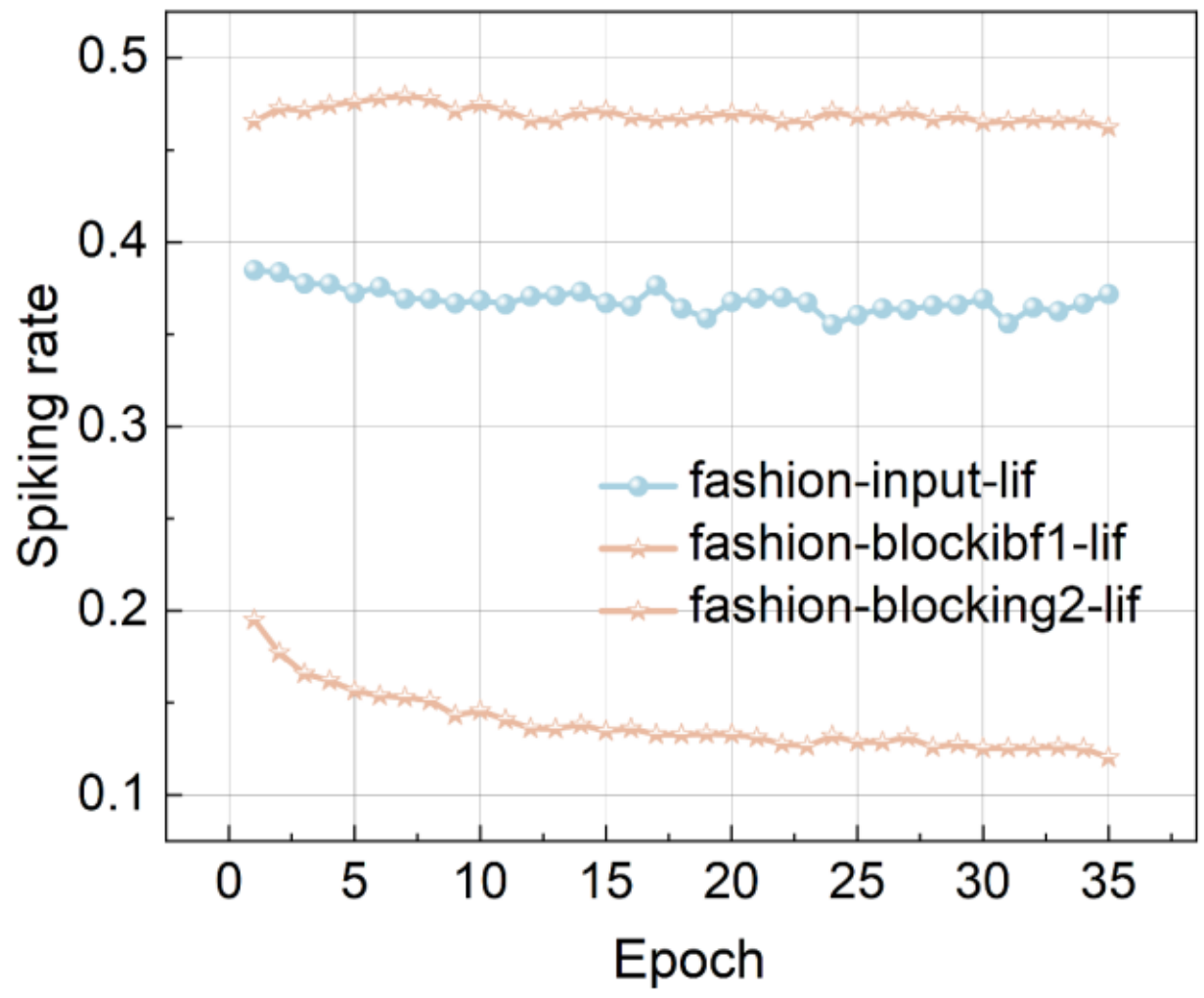

Figure S42. Changes in LIF Neuron Firing Frequency in the Fashionmnist Dataset.

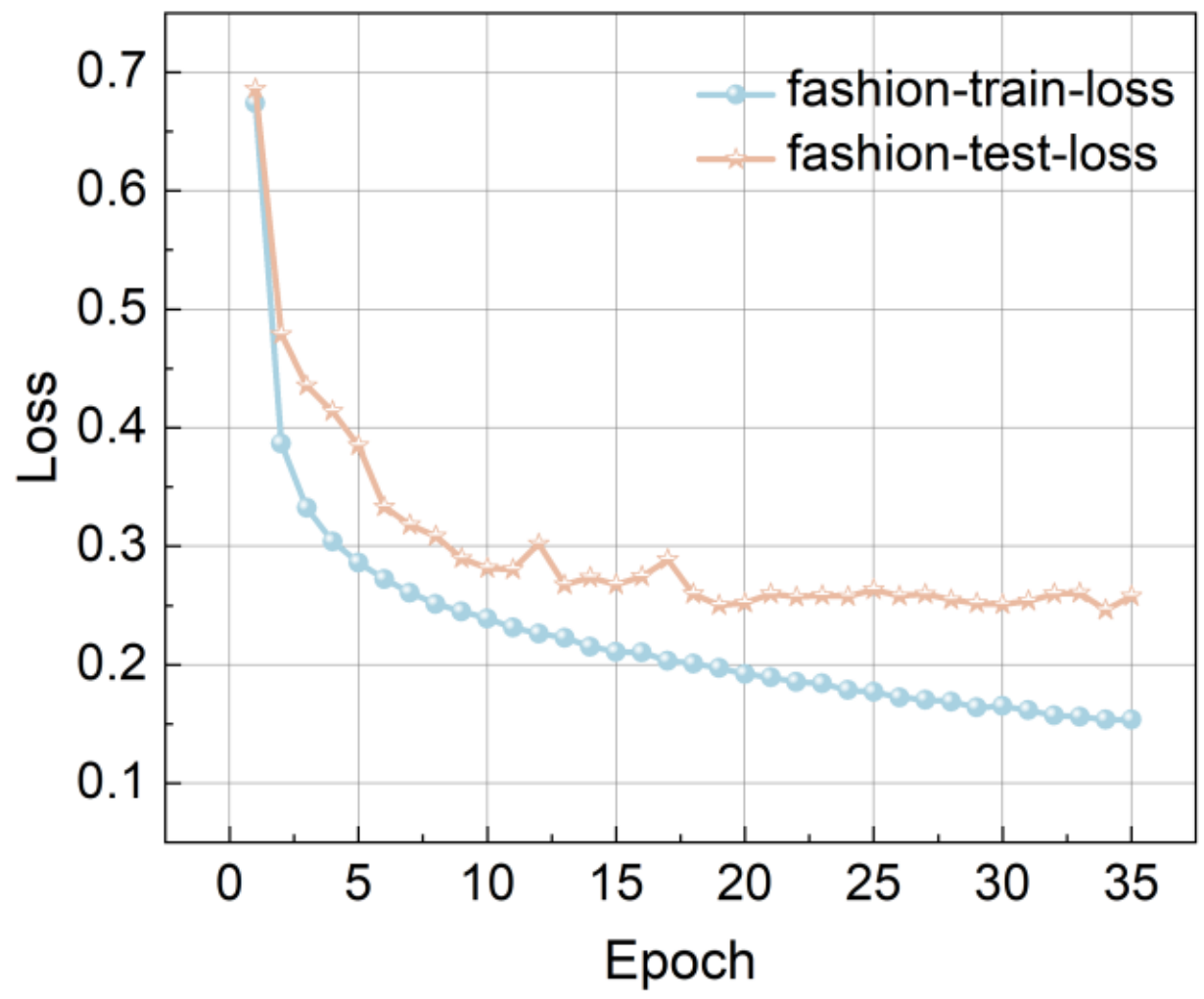

Figure S43. Changes in the Loss Function of the Fashionmnist Dataset.

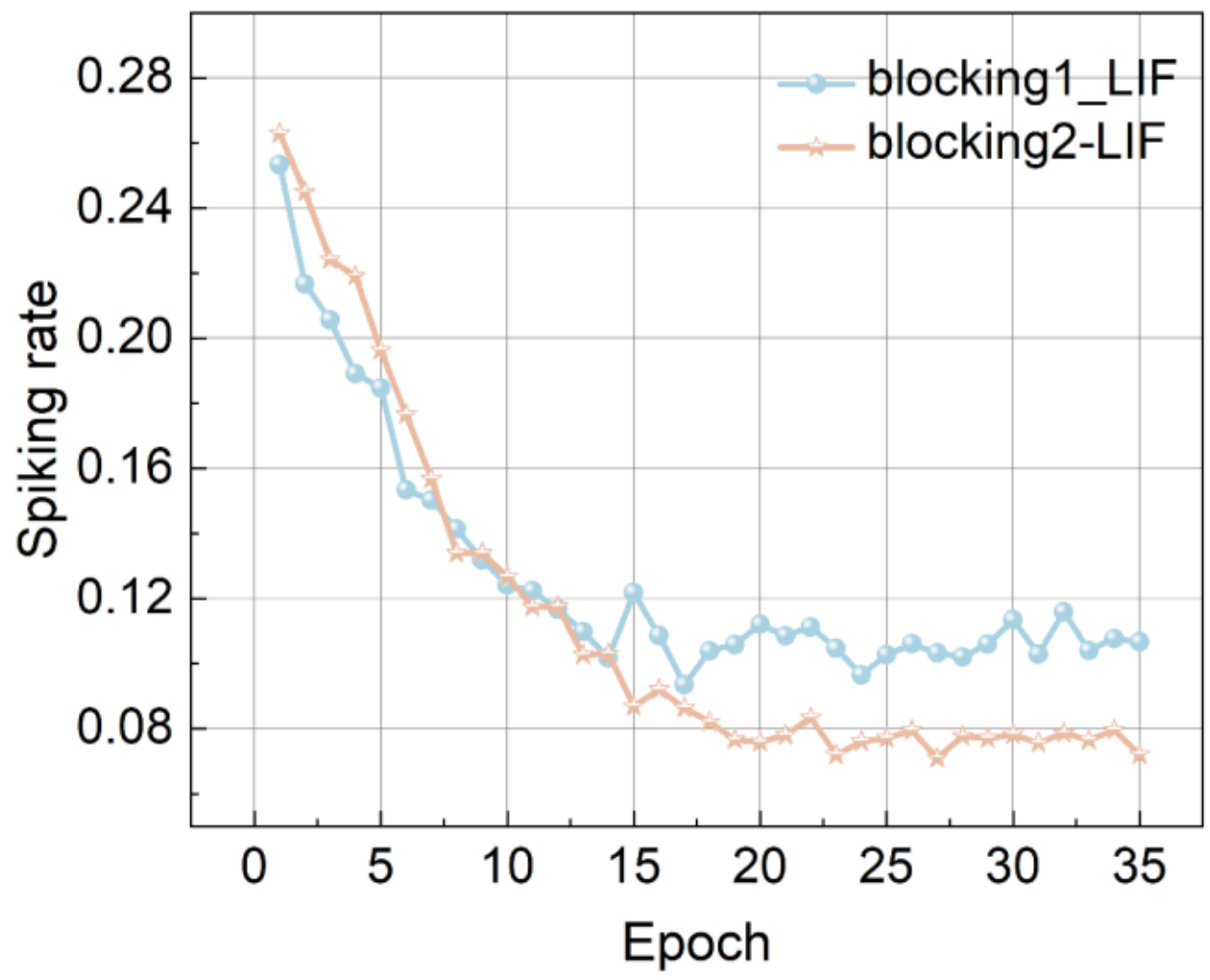

Figure S44. Variations in LIF Neuron Discharge Frequency in the Fire Dataset.

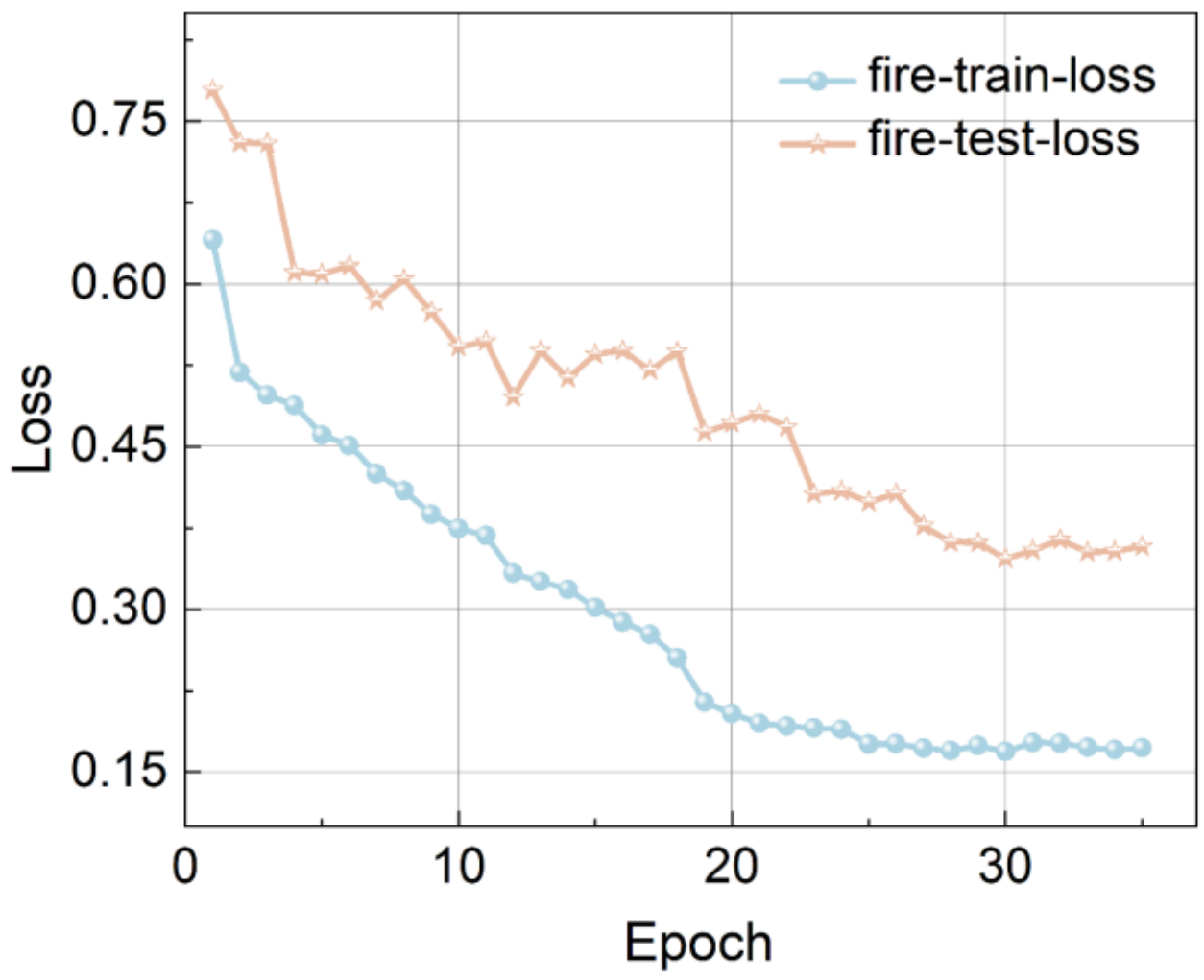

Figure S45. Changes in the Loss Function for the Fire Dataset.

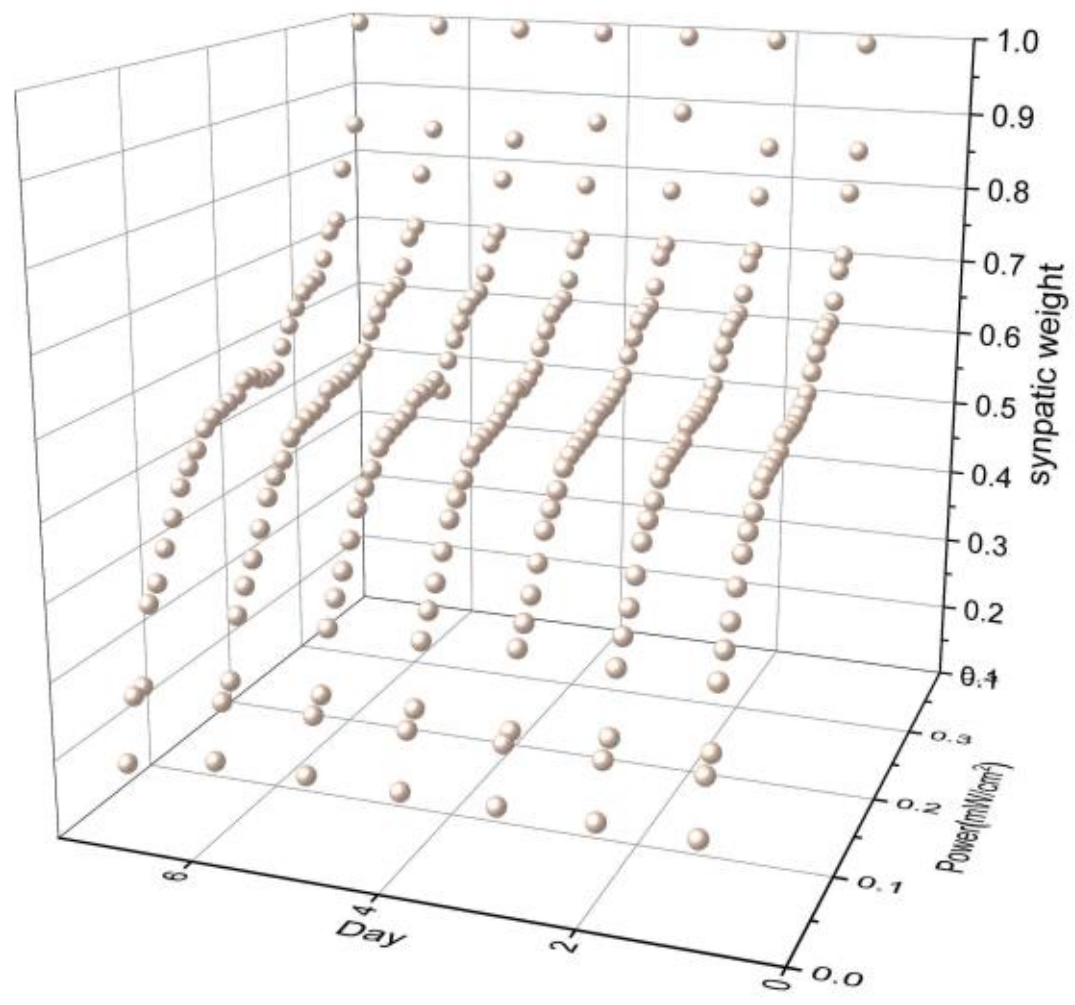

Figure S46. Conductance in different days.

Table S1. Deconvolution fitting results of time-resolved fluorescence curves of five components.

Component	PL: τ_1 (ns)	A_1 (%)	PL: τ_2 (ns)	A_2 (%)	Lasing: τ_3 (ps)
$RbPbBr_3$	1.7	64.2	7.7	35.8	5.4
$Cs_{0.23}Rb_{0.77}PbBr_3$	1.4	68.7	6.2	31.3	6.3
$Cs_{0.5}Rb_{0.5}PbBr_3$	1.1	62.1	5.3	37.9	6.7
$Cs_{0.71}Rb_{0.29}PbBr_3$	1.28	66.6	4.7	33.4	6.7
$CsPbBr_3$	1.87	71.4	6.7	28.6	5.4

Supporting Information Note S1: Rate Equation Analysis of Lasing

To further explore the coupling mechanism between the gain medium and the cavity, we conducted a standard rate equation analysis consistent with prior reports to derive the corresponding spontaneous emission coupling factor β. The carrier density N and photon density S can be described by the following rate equations:

$$\frac{dN}{dt} = \frac{\eta p_{pump}}{\hbar\omega V} - \frac{N}{\tau_{sp}} - v_g g S \quad (S1)$$

$$\frac{dS}{dt} = \Gamma v_g \ (g - vg_{th}) \ S + \frac{\Gamma\beta N}{\tau_{sp}} \quad (S2)$$

Where p_{pump} is the pump intensity, η the absorption efficiency of materials, and $\hbar\omega$ the photon energy of the pump laser. V denotes the active volume of the gain sample cavity, τ_{sp} the spontaneous emission lifetime of materials, g the net material gain, and g_{th} the threshold gain. Γ represents the optical confinement factor of the cavity and v_g the group velocity. Given that a 400 nm pulsed laser was used for material pumping, the time-dependent power P is expressed as:

$$P(t) = P_{peak} sech^2 \ (1.76 \frac{t}{\Delta t}) \quad (S3)$$

Where P_{peak} is the peak pump power, obtained from the measured average pump power P_{avg}, pulse duration Δt (120 fs), and repetition rate f_{pump} (1 kHz) using the following equation:

$$P_{peak} = P_{avg}\frac{f_{pump}}{\Delta t} \quad (S4)$$

The material gain g is a function of the carrier density N, and can be approximately expressed as:

$$g(N) = a(N - N_{tr}) \quad (S5)$$

Where a is the absorption cross-section or linear gain coefficient of materials, and N_{tr} is the transparency carrier density, which we approximate to 0 for simplicity. At steady state, the carrier density N and photon density S satisfy the relations $\frac{dN}{dt} = 0$ and $\frac{dS}{dt} = 0$, and N_{tr} is set to 0 for simplicity. We thus derive the steady-state form of the rate equations as follows:

$$\frac{\eta p_{pump}}{\hbar\omega V} - \frac{N}{\tau_{sp}} - v_g g S = 0 \quad (S6)$$

$$\Gamma v_g \; (g - vg_{th}) \; S + \frac{\Gamma\beta N}{\tau_{sp}} = 0 \quad (S7)$$

After eliminating N, we derived the steady-state solution for S as a function of the spontaneous emission factor β. We then adjusted this parameter to match the lasing threshold power obtained in our experiments. Plots of pump fluence versus emission intensity with different β values are presented on a logarithmic scale, as shown in Figure X of the main text and Figure X of the Supporting Information. We find that β yields values of 0.064-0.091 for the best fit to the experimental data of $Cs_xRb_{1-x}PbBr_3$ ($0 \leq x \leq 1$) nanosheets.

Table S2. Detailed Growth Parameters for 2D $Cs_xRb_{1-x}PbBr_3$ ($0 \leq x \leq 1$) Nanosheets with Different Compositions.

**Component**	**Precursor Ratio (Cs:Rb)**	**T_{source} (°C)**	**$T_{substrate}$ (°C)**	**Ar Flow (SCCM)**	**Growth Time (min)**
$CsPbBr_3$	1:0	520	325	30	15
$Cs_{0.81}Rb_{0.19}PbBr_3$	4:1	470	315	40	20
$Cs_{0.72}Rb_{0.28}PbBr_3$	7:3	450	305	45	20
$Cs_{0.58}Rb_{0.42}PbBr_3$	2:1	425	285	50	25
$Cs_{0.48}Rb_{0.52}PbBr_3$	1:1	410	275	55	30
$Cs_{0.31}Rb_{0.69}PbBr_3$	3:7	380	245	65	45
$Cs_{0.24}Rb_{0.76}PbBr_3$	1:4	365	230	75	50
$RbPbBr_3$	0:1	350	215	80	60

Table S3. Comparison of event energy in perovskite memristor.

Reference No.	Device Type	Core Material	signal type	Energy consumption (fJ)
This work	memristor	$HfO_2/Cs_{0.81}Rb_{0.19}PbBr_3/$ PMMA	light	19
[1]	memristor	$A_{1-y}MA_y$-$PbI_{3-x}Cl_x/(PEA)_2PbI_4$	electricity	35000
[2]	memristor	$SnO_2/CsPbBr_3$/PMMA	electricity	5330
[3]	memristor	$Cs_2AgBiBr_6$	electricity	575,000
[4]	memristor	$AgNPs/(PEA)_2MA_4Pb_5I_{16}/PtNPs$	Light	100,000,000
[5]	memristor	$CsPbBr_3$	Light	393,000,000
[6]	memristor	$Na:MAPbBr_3$	Light	280,000
[7]	memristor	$PMMA/Cs_2AgBiBr_6T_2$	Light	5,800,000
[8]	memristor	$CsPbBr_3$	electricity	4,450,000
[9]	memristor	PEDOT:PSS/Active layer/PMMA	electricity	26,000,000
[10]	memristor	2D-$Sr_2Nb_3O_{10}$	electricity	140
[11]	memristor	CBB/MoS_2	Electricity	300,000
[12]	memristor	MoO_3-MoS_2/RbCsMAFA	electricity	1,800,000,000
[13]	memristor	$Cs_3Bi_{2-x}Li_xI_{9-2x}$	electricity	70,000
[14]	memristor	Al_2O_3/PECI	electricity	4,900
[15]	memristor	$CH_3NH_3PbBr_3$ (MAP-bBr_3)/ZnO	light	1,800,000,000

References

[1] Sun K, Wang Q, Zhou L, et al. 2D-3D perovskite memristor with low energy consumption and high stability for neural morphology calculation. *Sci. China Mater.* 2023; **66:** 2013-2022.

[2] Xie Z, Wu J, Tian J, et al. Additive-Engineered $CsPbBr_3$-Based Perovskite Memristors for Neuromorphic Computing and Associative Learning Applications. *ACS Appl. Mater. Interfaces*. 2025; **17:** 53704-53715.

[3] Tang J, Pan X, Chen X, et al. Flexible memristor based on lead-free $Cs_2AgBiBr_6$ perovskite for artificial nociceptors and information security. *Adv. Funct. Mater*. 2025; **35:** 2412375.

[4] Bousoulas P, Orfanoudakis S, Tsioustas C, et al. Interfacial Engineering of Two-Dimensional Perovskite Memristors toward Reliable and Flexible Optoelectronic Memory Arrays. *ACS Appl. Mater. Interfaces*. 2026; **18:** 19315-19326.

[5] Liu Z, Cheng P, Kang R, et al. All-inorganic $CsPbBr_3$ perovskite planar-type memristors as optoelectronic synapses. *ACS Appl. Mater. Interfaces*. 2024; **16:** 51065-51079.

[6] Wang Y, Cheng X, Chen S, et al. Sodium-enhanced perovskite reservoir for photonic in-sensor computing. *Nano Energy*. 2025; **138:** 110830.

[7] Gao K, Cao Z, Sun B, et al. An Intelligence Programmable Intrathecal Injection System Based on Tween 80-Confined Halide Perovskite Optoelectronic Memristor. *Adv. Mater*. 2026; **38:** e13992.

[8] Liu S, Guan J, Yin L, et al. Solution-processed synaptic memristors based on halide perovskite nanocrystals. *J. Phys. Chem. Lett.* 2022; **13:** 10994-11000.

[9] Khemnani M, Tripathi B, Thakkar P, et al. Investigating the role of interfacial layer for resistive switching in a hybrid Dion-Jacobson perovskite-based memristor. *ACS Appl. Electron. Mater*. 2023; **5:** 5249-5256.

[10] Kim S, Yoon C, Yim H, et al. Dual Bipolar Resistive Switching in Wafer-Scalable 2D Perovskite Oxide Nanosheets-Based Memristor. *Adv. Sci*. 2026; **13:** e17588.

[11] Yu Y, Yang Y, Tian Y, et al. Interfacial Engineering of $Cs_3Bi_2Br_9/MoS_2$ Heterostructure-based Memristors for Self-rectifying and Nociceptor Applications. *Adv. Funct.*

Mater. 2026; **38:** e32198.

[12] Kalemai G, Verykios A, Chatzigiannakis G, et al. Highly Robust Double Memristive Device Based on Perovskite/Molybdenum Oxide-Sulfide Compound Heterojunction System. *Adv. Electron. Mater.* 2025; **11:** 2400433.

[13] Gao J, Gao Q, Huang J, et al. Influence of lithium doping on volcanic-like perovskite memristors and artificial synaptic simulation for neurocomputing. *ACS Appl. Nano Mater*. 2023; **6:** 7975-7983.

[14] Panchanan S, Dutta S, Dastgeer G, et al. Mixed-dimensional cu-based perovskites for stable and energy-efficient neuromorphic memristors. *Adv. Funct. Mater*. 2026; **36:** e20665.

[15] Ge S, Huang F, He J, et al. Bidirectional photoresponse in perovskite-ZnO heterostructure for fully optical-controlled artificial synapse. *Adv. Opt. Mater*. 2022; **10:** 2200409.
